# De novo genome assembly of a foxtail millet cultivar Huagu11 uncovered the genetic difference to the cultivar Yugu1, and the genetic mechanism of imazethapyr tolerance

**DOI:** 10.1186/s12870-021-03003-8

**Published:** 2021-06-12

**Authors:** Jie Wang, Shiming Li, Lei Lan, Mushan Xie, Shu Cheng, Xiaolong Gan, Gang Huang, Guohua Du, Kang Yu, Xuemei Ni, Baolong Liu, Guoxiong Peng

**Affiliations:** 1 Genetic, Engineering Research Center, School of Life Sciences, Chongqing University, Chongqing, 401331 China; 2 BGI Institute of Applied Agriculture, BGI-Shenzhen, Shenzhen, 518120 China; 3 BGI‐Agro Seed Service (Wuhan) Co., Ltd, BGI-Shenzhen, Wuhan, 430090 China; 4 Key Laboratory of Adaptation and Evolution of Plateau Biota, Northwest Institute of Plateau Biology, Chinese Academy of Sciences, Xining, 810008 Qinghai China; 5 Key Laboratory of Crop Molecular Breeding, Xining, 810008 Qinghai China

**Keywords:** *Setaria italica*, Genome, Comparative genomic analysis, *Acetohydroxy acid synthase*, Imazethapyr tolerance

## Abstract

**Background:**

*Setaria italica* is the second-most widely planted species of millets in the world and an important model grain crop for the research of C4 photosynthesis and abiotic stress tolerance. Through three genomes assembly and annotation efforts, all genomes were based on next generation sequencing technology, which limited the genome continuity.

**Results:**

Here we report a high-quality whole-genome of new cultivar Huagu11, using single-molecule real-time sequencing and High-throughput chromosome conformation capture (Hi-C) mapping technologies. The total assembly size of the Huagu11 genome was 408.37 Mb with a scaffold N50 size of 45.89 Mb. Compared with the other three reported millet genomes based on the next generation sequencing technology, the Huagu11 genome had the highest genomic continuity. Intraspecies comparison showed about 94.97 and 94.66% of the Yugu1 and Huagu11 genomes, respectively, were able to be aligned as one-to-one blocks with four chromosome inversion. The Huagu11 genome contained approximately 19.43 Mb Presence/absence Variation (PAV) with 627 protein-coding transcripts, while Yugu1 genomes had 20.53 Mb PAV sequences encoding 737 proteins. Overall, 969,596 Single-nucleotide polymorphism (SNPs) and 156,282 insertion-deletion (InDels) were identified between these two genomes. The genome comparison between Huagu11 and Yugu1 should reflect the genetic identity and variation between the cultivars of foxtail millet to a certain extent. The Ser-626-Aln substitution in acetohydroxy acid synthase (*AHAS*) was found to be relative to the imazethapyr tolerance in Huagu11.

**Conclusions:**

A new improved high-quality reference genome sequence of *Setaria italica* was assembled, and intraspecies genome comparison determined the genetic identity and variation between the cultivars of foxtail millet. Based on the genome sequence, it was inferred that the Ser-626-Aln substitution in *AHAS* was responsible for the imazethapyr tolerance in Huagu11. The new improved reference genome of *Setaria italica* will promote the genic and genomic studies of this species and be beneficial for cultivar improvement.

**Supplementary Information:**

The online version contains supplementary material available at 10.1186/s12870-021-03003-8.

## Background

Foxtail millet (*Setaria italic*) is a climate-resilient cereal crop domesticated in northern China more than 8000 years ago [1] and is mainly cultivated in arid and semi-arid regions. It also serves as a model crop for the study of C4 photosynthesis, stress tolerance and bioenergy traits, due to its small genome size and short life-cycle [2–6]. The genome assemblies of two foxtail millet strains, ‘Yugu1’ and ‘zhang gu’, were published in 2012, which have accelerated the research of biology and genetics of this species [7–9]. The ‘Yugu1’ genome assembly based on Sanger sequencing, has higher contiguity than the short-read genome assembly of ‘Zhang gu’ (contig N50: 126.3 kb versus 25.8 kb) and is currently used as a reference genome of foxtail millet. Although the ‘Yugu1’ reference genome is well-annotated and has been used in many studies, its total length of 401 Mb only covers ~ 80% of the estimated genome size (~ 510 Mb,) based on k-mer analysis and is distributed in 6,778 contigs [9]. The missing sequences are mainly long repeats with lengths of 5–10 kb, which are difficult to assemble at that time using Sanger or next generation sequencing strategy. Although the function of repetitive sequences is seldom researched in foxtail millet, they have proved to play important roles in the regulation of gene expression and genome evolution in other cereal crops [9]. The hard-to-sequence gaps in the scaffolds reside in the intergenic regions and may also contribute to the regulation of gene expression. As quantitative genetic analyses have revealed that many causal variations underlying phenotype changes are from regulatory regions, a complete and high-quality genome assembly is crucial to understand of the genetic mechanisms of climate-resilient features, genome evolution and important agronomic traits in foxtail millet.

The herbicide-resistance trait is an important trait for foxtail millet. Foxtail millet had the relatively small grain, which made the weed problem worse. The weed seeds were similar to the foxtail millet, which was hard to clean the weed seed in seed production. Moreover, the small seed produce the small plant in the germination stage. It is easy to be covered by the weed. The production reduction will be very serious without weed controlling [10]. The use of herbicides is a major measure to control weeds effectively in modern agriculture. Through the original millet cultivars did not have the ability to resist the herbicides, several herbicide-resistance genes had been imported into the modern millet cultivars through the hybridization with wild related species, or chemical mutagenesis. The different herbicide-resistance genes had the resistance to the different herbicide, and the different resistance to the same herbicide [11]. It will be very helpful of understanding the molecular mechanism of the herbicide-resistance for agriculture production. In the breeding process, the breeder usually used several parents to obtain one cultivar with the herbicide-resistance, which lead to trace the resource of the herbicide-resistance gene hardly. The genome assembly of the cultivar will provide an easy way to uncover the mechanism of the herbicide-resistance.

Here, we provide the assembly of a high-quality Huagu11 reference genome through three technologies: single-molecule real-time sequencing (SMRT) chromosome conformation capture sequencing (Hi-C) and next generation sequencing (NGS). Compared with former cultivars, Huagu11 was planted widely in western China, which should present a special ecotype of Foxtail millet. This new reference genome is helpful for further analysis and comparison of genomic diversity of intraspecific genomes in foxtail millet. By intensively aligning the Yugu1 and Huagu11 genomes, we identified 969,596 SNPs, 156,282 small insertions/deletions (indels, length shorter than 100 bp) and more than 19 Mb of presence/absence-variation (PAV, length longer than 500 bp) sequences between these two millet genomes. Interestingly, our comparative genomics analysis revealed a wide range of intraspecific gene-order variations: about 7.54% of genes were non-syntenic between these two genomes. The two cultivars had different phenotype in the grouting days, plant height, glume color, thousand grain weight, and the response to the imazethapyr (Table S1). Considering the amino acid substitution in AHAS could produce the resistance against the herbicide imazethapyr in several plants [12], the sequences of *AHAS* were aligned to understand its functional diversity in the two cultivars.

## Results

### Genome sequencing and assembly

Three technologies were combined to sequence and assembly the Huagu 11 genome: SMRT, Hi-C and NGS. In total, we generated 64.43 Gb (~ 155 ×) SMRT sequences, 63.31 Gb high quality clean paired HiSeq reads (PE150) and 34.34 Gb (~ 83 ×) effective Hi-C reads (Table S2). The K-mer analysis estimated that the genome size of Huagu11 was 456 Mb (Figure S1; Table S3). We assembled the genome as the pipeline previously reported. The initial assembly was performed using PacBio SMRT data alone and resulted in 379 contigs with a N50 length of 5.39 Mb (Table 1). The contigs were corrected with the NGS reads and then scaffolded using Hi-C data (Table S4; Figure S2). The total assembly size of the Huagu11 genome is 408.37 Mb (Table 1), which is similar to the recently updated Yugu1 genome (405.73 Mb). The assembled genome of Huagu11 contains nine scaffolds. The scaffolds number was corresponded to the chromosome number, which meant we got 9 pseudochromosomes from Hi-C. The scaffold N50 size is 45.89 Mb (Table 1), which constituted approximately 98% of the whole genome. Compared with the other three reported millet genomes, the Huagu11 genome has the minimum number of contigs and the longest contig N50 length, which indicates the highest genomic continuity (Table 1; Table S5).

**Table 1:** Summary of genome assembly and annotation

Assembly				
		N50 (size/number)	N90 (size/number)	Total length
Genome assembly	Contig	5.4 Mb/25	1.4 Mb/86	408 Mb
	Scaffold	45.9 Mb/5	36.3 Mb/9	408 Mb
	Chromosomes	9 chromosomes (from 173 contig)		408 Mb
Annotation				
Transposable elements	Type		Total length	
	Total		192 Mb (46.9%)	
	Retroelements		141 Mb (34.57%)	
	DNA transposons		47.1 Mb (11.5%)	
Noncoding RNAs	Type	Copies	Total length (kb)	
	miRNA	161	21.4	
	tRNA	976	73.3	
	rRNA	91	10.8	
	snRNA	503	59.2	
Protein coding genes	Total number	Supported by transcriptome data	Function assigned	High confidence
	41,984	23,861	35,403	36,652

Three methods were used to evaluate the quality and completeness of the assembled Huagu11 genome. First, approximately 98.3% (1,351 of 1,375) of embryophyta genes were detected in our assembly using Benchmarking Universal Single-Copy Orthologs (BUSCO) analysis, a percentage similar to that for the Yugu1 genome (97.8%) (Table S6). Second, while the Hiseq short reads was mapped back to the assembly using Burrows-Wheeler-Alignment (BWA) tool with default parameters, about 98.30% of the total reads could be well mapped, which covered over 99.12% of the assembly (Table S7). We also found that about 97.75% of the assembly was covered by more than 20 folds of short NGS reads, which ensured high accuracy of assembly at the single nucleotide level (Table S7). Third, Expressed Sequence Tag (EST) sequences from the NCBI was aligned to the Huagu11 genome assembly by the BLAT software [13] with default parameters. For the 19,441 ESTs longer than 500 bp, about 98.61% of the ESTs could be mapped to the assembled genome, and 95.86% of the ESTs were credited as complete sequences because at least 90% of the EST nucleotide could be mapped to one continuous scaffold (Table S8). These results indicated the assembled genome of Huagu11 had a high quality.

### Genome annotation

We used the methods of ab initio structure analysis and homology comparison to search the genome sequences to analyze the repetitive sequences. A total of 182 Mb (44.63% of the assembly genome) were repetitive sequences (Table S9). The most abundant subtypes were Gypsy-like and Copia-like elements, representing 21.55% and 10.33% of the assembly genome, respectively. The remaining transposable elements were DNA transposons (11.53%), long interspersed nuclear elements (LINEs; 2.46%), short interspersed nuclear elements (SINEs, 0.13%), and uncharacterized repeats (0.83%) (Table S9).

**Fig. 1:**
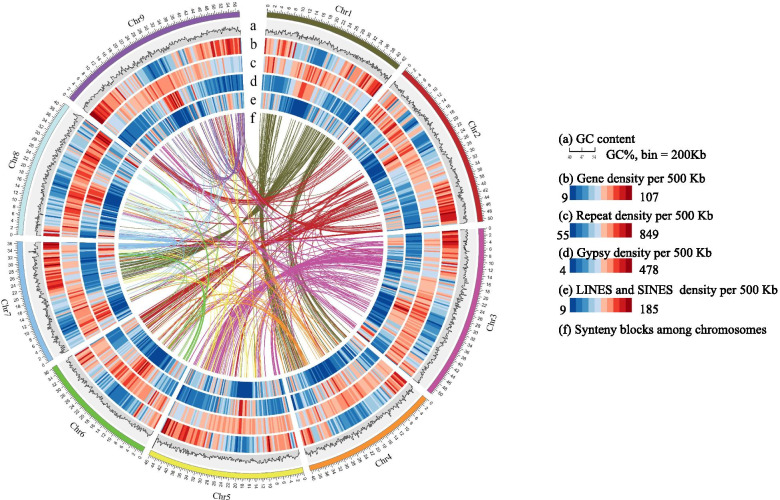
Synteny and distribution of features in the foxtail millet genome. **a** GC content in 200 Kb window. **b** Gene density in 500 Kb window. **c** Repeat density in 500 Kb window. **d** Gyspy density in 500 Kb window. **e** LINEs and SINES density in 500 Kb window. **f** Synteny blocks among homologous foxtail millet chromosomes

We annotated the protein coding genes in huagu11 genome by combining the results of protein homology prediction, RNA sequence prediction and ab initio prediction. A total of 42,932 protein coding genes were predicted in the Huagu11 genome, among which 36,652 genes were characterized as high confidence and 65.1% of the genes could be supported by transcriptome data from four different tissues (Table 1). We annotated the genome and found that 82.5% of the genes had known functions (Table S10). About 41,646 (99.19%) of the Huagu11 predicted genes were found in nine pseudochromosomes. The protein coding genes were mainly located in the chromosome arms and negatively correlated with density of transposable elements (Fig. 1). The predicted noncoding RNA genes included 91 ribosomal RNA genes, 976 tRNA genes, 161 microRNA (miRNA) genes and 503 small nuclear RNA (snRNA) genes (Table S11).

### Intraspecies comparison of the genomes of Yugu1 and Huagu11

**Fig. 2:**
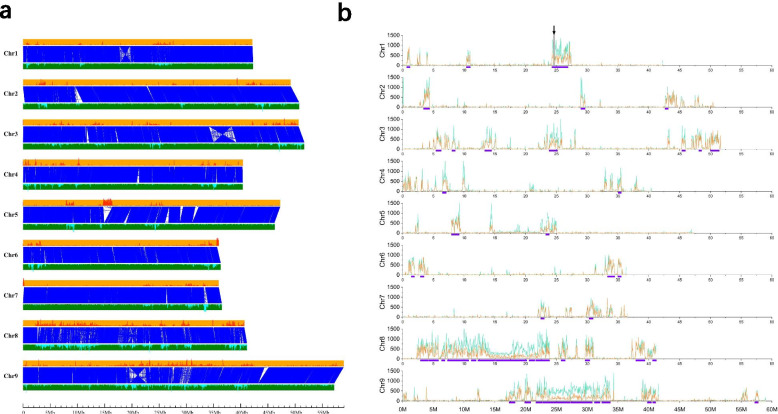
Whole-genome comparison of Yugu1 and Huagu11 genomes. **a** One-to-one syntenic blocks between Yugu1 and Huagu11 genomes. Orange lines and green lines represent Yugu1 chromosomes and Huagu11 chromosomes, respectively. The red plots in the orange lines and the cyan plots in the green lines represent the identified PAV sequences in these two genomes. **b** The SNP and indel distributions were aligned the Huagu11 genome to Yugu1 genome by MUMmer software to identify genetic variants. 50 K bp per window, and cyan lines represent SNP numbers per window and orange lines represent total indels length (bp) per window (including insertions and deletions). The purple lines below the x-axis represent SNP-enriched regions greater than 450 Kb. The dark arrow indicated the gene locus of AHAS, which accounted for the herbicide resistant trait of Huagu11

Interspecies comparison with other species had been made in each published foxtail millet genome. Though, the intraspecies comparison was still absent. Considering the quality of the Yugu1 genome sequence is very close to the Huagu11, it would be a good opportunity to carry out the interspecies comparison. When we aligned the Huagu11 genome to the Yugu1 genome in the chromosome scale MUMmer software [14], approximately 94.66% of the Huagu11 genome sequence (381,136,172 bp) matched with 94.97% of theYugu1 genome sequence (381,111,204 bp) in one-to-one syntenic block patterns (Fig. 2a).This indicated that most regions of the genomes of these two species are stable. We identified 1858 inversions, 462 intra-translocations, and 631 inter-translocations between these two genomes which account for ~ 17.61 Mb genome regions in total (Table S12). Four chromosome inversion were observed in the one-to-one syntenic blocks (Fig. 2a). The non-syntenic sequences between the two genomes were mostly transposable elements, and the rest were dispersed genes and intraspecies-specific low-copy sequences.

By comparing the Yugu1 and Huagu11 genome, while only DNA fragments larger than 500 bp were counted, we also identified 18,584 Huagu11-specific genomic segments (19.43 Mb in total) and 18,437 Yugu1-specific genomic segments (20.53 Mb in total). Most of the PAV fragments (99.04% of Huagu11-specific genomic segments and 98.43% of Yugu1-specific genomic segments) were shorter than 5 kb (Figure S3). Only 178 PAV sequences longer than 5 kb were found in Huagu11, and 294 PAV sequences longer than 5 kb were found in Yugu1. These PAV sequences were nonuniformly distributed throughout the genome, and some of them were located in clusters (Fig. 2a). Interestingly, there were only 1.12 Mb Yugu1 specific PAV sequences distributed in clusters, while 3.73 Mb Huagu11 specific PAV sequences were distributed in clusters. We identified 627 protein-coding transcripts in Yugu1-specific genomic segments and 737 protein-coding transcripts in Huagu11-specific genomic segments in these PAV regions with the parameters of at least 75% of coding sequences were overlap with PAV sequences. The annotation and classification of the specific genes showed that the enriched networks of the specific genes of Huagu11 were Plant − pathogen interaction, Indole alkaloid biosynthesis, Betalain biosynthesis, Tyrosine metabolism, Phenylalanine metabolism, Histidine metabolism, Isoquinoline alkaloid biosynthesis, Anthocyanin biosynthesis, Alpha − Linolenic acid metabolism (Figure S4; Table S13). The enriched networks of Yugu1 were Oxidative phosphorylation, Ribosome, Thiamine metabolism, Mismatch repair, Pantothenate and CoA biosynthesis, Base excision repair, Sulfur metabolism, Benzoxazinoid biosynthesis, Endocytosis and Ether lipid metabolism (Figure S5; Table S14). Interestingly, the enriched networks of the specific genes of Huagu11 contained the anthocyanin biosynthesis, which was possibly responsible for the purple glume traits.

OrthoFinder (v2.5.1) was used to identify homologous genes and investigate gene duplication events between Huagu11 and Yugu1 based on an all-verse-all blastp alignment with default parameters [15, 16]. Four thousand five hundred eighty-three genes were clustered into 917 Huagu11 specific orthologue groups and 1781 genes were clustered into 418 Yug11 specific orthologue groups. One thousand and thirty-seven of single copy gene in Huagu11 were duplicated in Yugu1 with 2,798 duplicated genes (2.70 per gene) and 1,785 of single copy gene in Yugu1 were duplicated in Huagu11 with 5,750 duplicated genes (3.22 per gene). Eight thousand, six hundred six genes of Huagu11 and 8829 genes of Yugu1 were clustered into 3,031 orthologue groups with more than one gene of both cultivars in the same orthologue group (Table S15).

When we aligned the Huagu11 genome with the Yugu1 pseudochromosomes genome to identify genetic variations between the Yugu1 and Huagu11 genomes, a total of 969,596 SNPs and 156,282 indels (totally 617,674 bp) were identified with an average of 2.42 SNPs and 0.39 indels (1.54 bp) per kb. There was a positive correlation of the distributions between SNPs and indels (Pearson’s correlation *R* = 0.9859, *P* < 0.001; Fig. 2b). We found these SNPs were not evenly distributed on the chromosomes, but clustered together (Figure S6). The windows with SNP density more than 1.5 folds of the average density of the genome (≥ 180 SNPs per 50 Kb) was defined as the SNP enrichment regions. About 1593 windows were identified as SNP-enriched regions which accounted for 19.78% of the whole genome and contained 89.55% of SNPs. The windows adjacent in 100 Kb were merged while the regions more than 450 kb were illustrated (Fig. 2b). It was a high possibility that these SNP-enriched regions in Huagu11 and Yugu1 were derived from the different genetic resources and contributed to the phenotype diversity in genetics.

### The genetic mechanism of the resistance against the herbicide imazethapyr in Huagu11

**Fig. 3:**
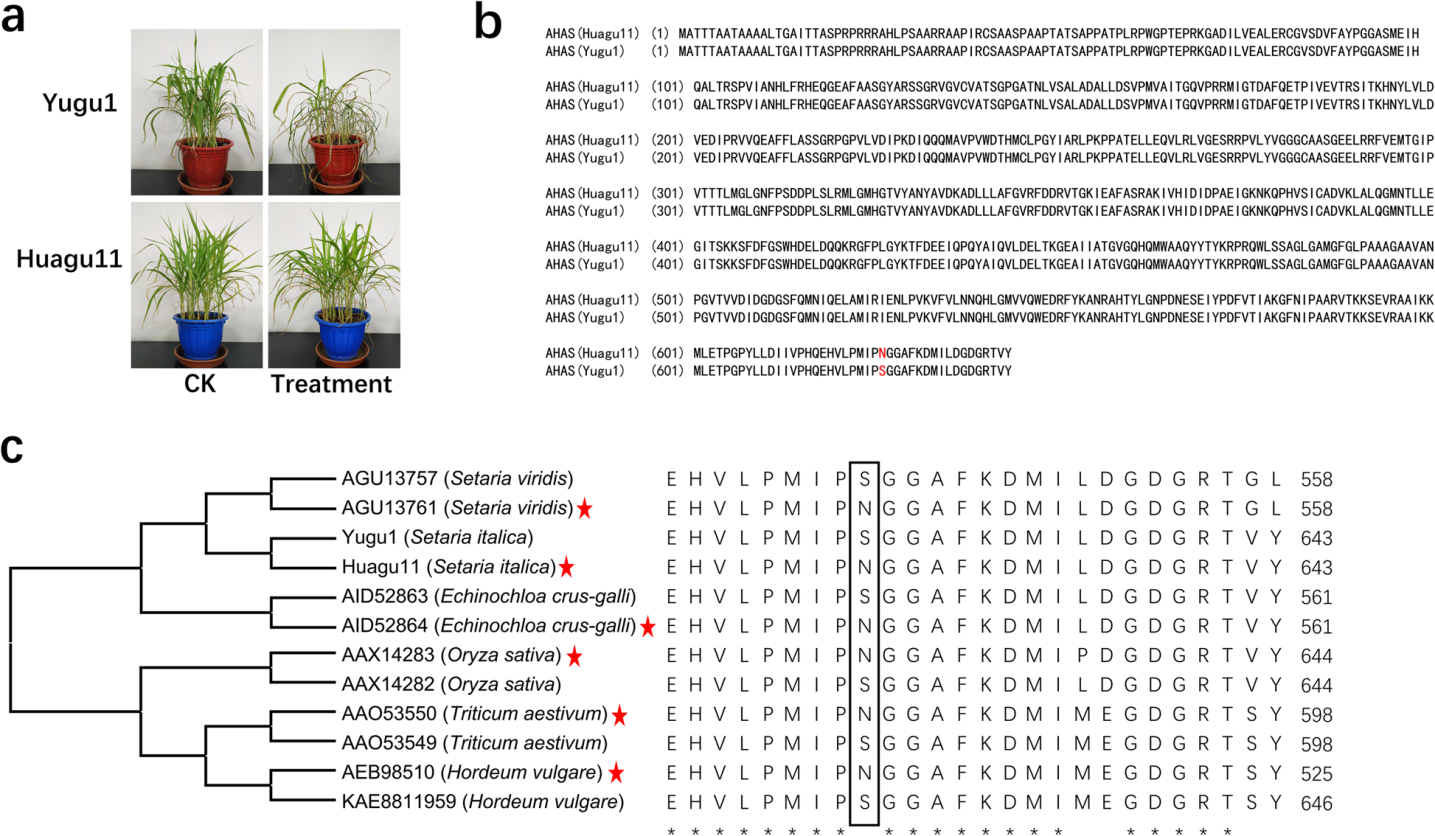
The phenotype and genetic differences of Huagu11 and Yugu1 in the resistance to the herbicide imazethapyr. **a** The phenotypes of Huagu11 and Yugu1 after treatment with the herbicide imazethapyr. The Yugu1 leaves wilted, withered, and died 7 days later after the treatment of 0.25% Imazethapyr, whereas Huagu11 grew normally. **b** The protein alignment of AHAS from Huagu11 and Yugu1. The red letters indicate different amino acids. **c** Comparative sequence alignment of AHAS showing phylogenetic relationships among members of the grass lineage. The rectangular box indicates the position of the amino acid substitution providing resistance against imidazolinone herbicides. The red * after the cultivars indicates that the cultivar has resistance against imidazolinone herbicides. The * under the sequences indicates the identical position of amino acids in all cultivars

In Yugu1 and Huagu11, there is an obvious difference in the resistance against herbicide imazethapyr (Fig. 3a). The resistance to the herbicide imazethapyr was mainly relative to the *AHAS* gene in plants [17]. *AHAS* was found to locate at 24,623,024–24,624,955 bp of chromosome 1 of Huagu11, which was coincidental with the SNP-enriched regions (Fig. 2b). The length of the contig containing the *AHAS* gene was 6,760,944 bp with 3,606,289 bp before the translation initiation codon and 3,152,723 bp behind the termination codon, which means the genome sequence of Huagu11 could give the all-round information about *AHAS*. The sequences of *AHAS* were picked up from the other three genomes Yugu1, Zhuanggu, and TT8 of *Setaria italica* [18]. The contig of Yugu 1 was 207,997 bp in length, which was far shorter than Huagu11. The *AHAS* sequence was incomplete in the Zhanggu genome and was missed in the gene set of the TT8 genome. The phenomenon clearly showed that the quality of the Huagu11 genome was better and more useful than other genomes. Compared with Yugu1, four single nucleotide mutations existed in the *AHAS* gene of Huagu11 (Figure S6), which caused only one amino acid change in the encoded proteins (Fig. 3b). The amino acid Ser in site 626 of the AHAS protein of Yugu1 was changed into Asn in Huagu11 (Fig. 3b). The alleles variation s626n could be found in *Setaria viridis*, *Oryza sativa*, *Echinochloa crus-galli*, *Hordeum vulgare*, and *Triticum aestivum* [19–23]. All cultivars carrying Asn had a resistance to imazethapyr in these species, while the cultivars with Ser did not (Fig. 3c). To verify the single nucleotide mutation, the nucleotide sequences of *AHAS* were amplified and sequenced from the four cultivars and the other two cultivars. The single nucleotide mutation existed exactly in Huagu11 and Y6492 with the herbicide resistance. The remaining cultivars had the same alleles as Yugu1 and were sensitive to the herbicide imazethapyr (Figure S8). The whole coding of AHAS and the SNP of s626n could be found in the transcriptome data of the leaf of Huagu11. All results suggested that s626n was responsible for the resistance to imazethapyr in Huagu11.

### Discussion

The assembly genome of Huagu11 had the highest continuity than previous genomes, which should be attributed to the use of the SMRT sequencing and Hi-C [24]. The genome of Yugu1 was based on the sanger sequencing technology [9], while Zhanggu and TT8 were based on the whole genome shotgun–next-generation sequencing strategy. The SMRT sequencing technology produced high reading length, which made it easy to obtain the longer contigs. The HiC technology could help to produces the longer scaffolds with longer contigs and anchor the longer scaffolds to chromosomes easily. The advanced technologies made the genome assembly economical and convenient. The better reference genome will assist in madding the foxtail millet the model plant for the research of C4 photosynthesis and abiotic stress tolerance.

The availability of high-quality assembled genomes for both Huagu11 and Yugu1 will provide a distinctive opportunity for in-depth comparison of intraspecific genomes. In one-to-one syntenic blocks, approximately 96% of the Huagu11 and Yugu1 were mutually syntenic, which was far higher than the value of the B73 and Mo17 maize genomes. Only 60% of the B73 and Mo17 genomes were able to be aligned as one-to-one blocks [25]. Through the genome sequences of Huagu11 and Yugu1 had the high syntenic value, extensive PVA, SNP, indel variation, as well as structural variations could be found in the intraspecific comparison. The Yugu1 and Huagu11 genomes each contained approximately 20 Mb of PAV sequence, including 1364 (627 in Yugu1 and 737 in huagu11) high-stringency PAV genes. There were 1858 inversions, 462 intra-translocations, and 631 inter-translocations between these two genomes, which account for ~ 17.61 Mb genome regions in total. A total of 969,596 SNPs and 156,282 indels (totally 617,674 bp) were identified between the two cultivars. All these variations could cause the phenotype difference in the two cultivars. For example, a SNP, causing one amino acid change in the *AHAS* gene, was responsible for the resistance to imazethapyr in Huagu11. Further evaluating the contribution of these differences to the phenotypic variations of agronomic traits would be an interesting future pursuit.

## Conclusions

A new improved high-quality reference genome sequence of *Setaria italica* was assembled based on SMRT sequencing and Hi-C mapping technologies. The total assembly size of the Huagu11 genome was 408.37 Mb with a scaffold N50 size of 45.89 Mb, which was the highest genomic continuity of *Setaria italica* to date. Intraspecies comparison showed about 96% of the Yugu1 and Huagu11 genomes could be identified as one-to-one blocks with four apparent chromosome inversion. The Huagu11 genome contained approximately 19.43 Mb PAV with 627 protein-coding transcripts, while Yugu1 genomes had 20.53 Mb PAV sequences encoding 737 proteins. There were also 969,596 SNPs and 156,282 indels. Based on the genome sequence of Huagu11, it was found that the Ser-626-Aln substitution in AHAS was responsible for resistance to imazethapyr in Huagu11. The new improved reference genome of *Setaria italica* will promote the genic and genomic studies of this species and be beneficial for cultivar improvement.

## Methods

### Materials

The foxtail millet cultivar Huagu11 was bred by the BGI Institute of Applied Agriculture (ShenZhen, China). Huagu11 was derived from the cross of Yugu18 and Y6492 and carried the resistance to the herbicide imazethapyr.

### Sampling and sequencing

The CTAB extraction method was employed for the genomic DNA extraction from the leaf of an individual of the foxtail millet line ‘Huagu11’ (NCBI Taxonomy ID: 4555) [26]. All sequencing libraries are constructed according to the quality requirements of sequencing equipment. The DNA library was prepared based on the PacBio platform, and the SMRT clock template preparation kit 1.0 (Pacific Biosciences Corporation, California, USA, 100–259-100) was used for the preparation of the PacBio library. The long-read DNA library was sequenced with PacBio RSII sequencer P6-C4 chemical method, and the movie length was 360 min. The Hi-C library was prepared according to the standard procedure [27]. RNA is isolated from different tissues (root, stem and leaf). The RNA-seq library was prepared using Illumina platform (Illumina, Santiago, CA, USA). We generated ~ 84.37 Gb of paired-end data, the raw genome reads were filtered using SOAPfilter software [28] and 63.31 Gb of high-quality reads were obtained for K-mer analysis and pilon analysis. PacBio sequencing obtained 64.43 Gb data, with an average read length of 8,657 bp (Table S1). About 132.57 Gb of raw data was sequenced from a Hi-C library and ∼34.34 Gb of valid reads were obtained after quality control. A total of 10.48 Gb, 15.43 Gb and 10.65 Gb of RNA-seq raw data were obtained for root, stem and leaf, respectively (Table S1). The raw reads were then filtered using SOAPfilter software, and finally, 10.25 Gb, 14.98 Gb, 10.37 Gb high-quality sequences were obtained for gene prediction (Table S1).

### Estimation of the genome size

K-mer analysis was employed to estimate the genome size. K-mers (K = 17) were counted by Jellyfish [29] using 14.5 Gb high-quality short reads. Genome size was estimated according to the formula: Genome Size = K-mer_num/Peak depth.

### Genome assembly

After removing the PacBio reads of less than 1 Kb in length and adapters, 64 Gb filtered reads, representing ~ 155 × sequencing coverage of the Huagu11 genome, were used for contig assembly with Falcon v0.3.0 [30]. Then, BLASR [31] was employed to map the readings obtained by PacBio SMRT back to contigs. SMRT was used to correct some sequencing errors. BWA mem was used to map the paired-end reads without Illumina PCR to the corrected contig [32], and further use high-quality reads for assembly [33].

### Chromosome assembly using Hi-C

The clean Hi-C reads were mapped to the genome assembly through BWA align. HiC-Pro was used for repetitive reading removal, classification and quality assessment [34]. The 25.11% of Hi-C data were valid reads. Raw counts of Hi-C links were aggregated and separately by using Juicer [35] and 3D-DNA [36]. The Juicebox [37] software was used to adjust the placement and orientation errors where the discrete chromatin interaction patterns were contradictory with the genetic map of Yugu1. According to the threshold of contact frequency, the sequence is divided into 9 groups. A total of 181 scaffolds (representing 98.60% total length) were anchored to chromosomes in *S. italica* (Table S3).

### Transposable elements annotation

Both of De novo prediction and homology-based alignment were used to identify transposable elements. The De novo repeat database was built by a combination of the results of LTR_Finder [38], PILER [39], and RepeatScout [40]. This De novo repeat database together with Repbase [41] were used to identify repeats by RepeatMasker [42] and to identify repeat related proteins by RepeatProteinMask (http://www.repeatmasker.org/). RepeatMasker and TRF [43] were used to annotate the tandem repeats. The results above were combined according to their physical positions and transposable elements were further classified by blast against Repbase.

### Gene annotation

Gene prediction was performed by homology-based prediction, de novo prediction and transcriptome-based prediction. For homology-based prediction, protein sequences from eight species (*Brachypodium distachyon*, *Hordeum vulgare*, *Oryza sativa*, *Sorghum bicolor*, *Setaria viridis*, *Zea mays*, *Setaria italica* (Zhang gu), and *Setaria italica* (Yugu1)) (Table S6) were mapped onto the Huagu11 genome by an E-value cutoff of 10^−5^, and then Genewise was used for gene structure annotation. AUGUSTUS [44] (Version 2.03) and FGENESH [45] (Version 1.3) were used for de novo prediction. RNA-seq data from three tissues were processed by HISAT2 [46] and StringTie [47] for transcriptome-based prediction. EvidenceModeler [48] software was used to combined the above results and get the final non-redundant reference gene set. InterproScan [49], Gene Ontology (GO) [50], Kyoto Encyclopedia of Genes and Genomes (KEGG), SwissProt [51], TrEMBL and Non-redundant protein NCBI databases were used to annotate the functions of the predicted genes by BLAST searches (E-value cutoff 1 × 10^−5^). tRNAscan-SE [52] was used to identify tRNA. Rfam database and Infernal [53] were used to identify noncoding RNAs (including rRNA, miRNA and snRNA) based on homologous alignment.

### Technical validation and assessment of the genome assembly and annotation

Using BWA to map short insert size reads back to assembly, mainly for evaluating the quality of genome assembly. In order to assess the integrity of the genome assembly, EST sequences from the NCBI was mapped to the Huagu11 genome assembly using BLAT with default parameters. To evaluate the completeness of the assembly, BUSCO [54] was executed with default parameters.

### Identification of PAV sequences, PAV clusters and PAV genes

We used an overlapped sliding-windows method to identify the PAV sequences in the Huagu11 and Yugu1 genomes. In order to identify the specific sequences of Huagu11, a series of 500 bp short reads with single end were generated by divided the Huagu11 genome into 500 bp overlapped windows with a step size of 100 bp. BWA was used to map these reads to Yugu1 genome with the parameter of “mem -w 500 –M”. If the sequences that could not be aligned to Yugu1, or the sequences that could be aligned to Yugu1 but the coverage was less than 20%, these sequences were merged according to their positions in the Huagu11 genome and were defined as Huagu11-specific sequences. Yugu1-specific sequences were identified in the same way. We compared the length distribution of PAV sequences between Huagu11 and Yugu1 genome and found most of the PAV sequences were less than 2 Kb (Figure S3). The adjacent PAV sequences (physical distance <  = 100 Kb) were merged together, and the PAV clusters were defined if more than 10% of the merged regions were covered by PAV sequences. The genes with more than 75% of the CDS regions covered by PAV sequences were defined as PAV genes. The function annotation of the PAV genes were obtained by BLAST examine (E-value cutoff 1 × 10^−5^) against the InterproScan, Gene Ontology (GO), Kyoto Encyclopedia of Genes and Genomes (KEGG), SwissProt, TrEMBL and Non-redundant protein NCBI databases. Significantly enriched networks were identified using the hypergeometric test [55].

### Investigation of homologue genes and duplicated genes between Huagu11 and Yugu1

Protein sequences from Huagu11 and Yugu1 were used to identify homologous genes and investigate gene duplication events by OrthoFinder v2.5.1 with default parameters based on an all-versus-all BLASTP alignment. The genes which could not be assigned into orthologue groups or were clustered into species-specific orthologue groups were considered as species-specific. The orthologue groups with one gene from Huagu11 and one gene from Yugu1 were considered as single copy homologous gene pairs. While the orthologue groups with one gene from Huagu11 and more than one gene from Yugu1, we regarded this gene was duplicated in Yugu1 and vice versa. If the gene numbers from one species was more than twice than those from another species, there was a gene duplication event between these two species in this orthologue group.

### Identification of chromosomal structural variations

We used MUMmer v3.23 (http://mummer.sourceforge.net/) to identify the SNPs and InDel between Huagu11 and Yugu1 using the following procedures: (1) Huagu11 genome was used as query genome to align with the reference genome Yugu1 by the nucmer utility under the parameters –mum. (2) the delta-filter utility was used to filter mapping noise and determine the one-to-one alignment blocks with parameters − 1 -r -q, SNPs were reported by the show-snps utility under the parameters -C -q -T, the adjacent single-base InDels were merged as one InDel.

The detection of inversions and translocations was completed by filtering the nucmer outputs using delta-filter utility through two sets of parameters settings: “-i 90 -1 -r -q” and “-i 90 -g -r -q”, respectively, where -1 was used to obtain one-to-one alignment blocks allowing for rearrangements and -g was used to obtain co-linear region which is the global alignment but not allowing rearrangements. These genomic rearrangement regions were finally defined as inversions or translocations depending on their locations and orientations adjacent to their neighboring blocks.

### The isolation of AHAS from foxtail millet cultivars

*AHAS* was identified from the foxtail millet genomes through BLAST searches (E-value cutoff 1 × 10^−5^). The amino acid sequences were aligned with clustalx software [56]. The primers AHAS-F (ATGGCCACGACGACCGCCGC) and AHAS-R (TCAATACACGGTCCTGCCAT) were designed to amplificated the whole coding sequences of *AHAS*, and the amplification products were 1932 length. Primers were synthesized by BGI Biological Technology Co., Ltd. The 50 μl reaction system included 10 μl 5 × GC Buffer, 4 μl 10 mmol dNTP, 0.5 μl 20 pmol primers, and 0.5 μl (100 ng) cDNAs (Thermo Fisher Science, Beijing, China) were supplemented with ddH2O. Using high-fidelity Phushion DNA polymerase (Thermo-Fisher Scientific) in the GeneAmp PCR System 9700 (Thermo-Fisher Scientific). The experimental conditions were as follows: one cycle at 98 °C for 2 min, 35 cycles at 98 °C for 10 s, 65 °C for 30 s and 72 °C for 2 min, followed by a cycle at 72 °C for 7 min.The PCR products were extracted from 1.0% agarose gels and cloned into the pGEM-T Easy Vector plasmid. The recombinant plasmid was then transformed into Escherichia coli DH5α cells and positive clones were sent to Shanghai Shenggong Biological Co., Ltd. for sequencing.

## Supplementary Information

**Additional file 1: Figure S1**. K-mer analysis to estimate the genome size of Huagu11.

**Additional file 2: Figure S2**. Hi C linkage density heat map of assembled contigs.

**Additional file 3: Figure S3**. Length distribution of PAV sequences between Huagu11 and Yugu1 genome. Most of the fragments were less than 5 Kb.

**Additional file 4: Figure S4**. The enriched networks of the specific genes of Huagu11.

**Additional file 5: Figure S5**. The enriched networks of the specific genes of Yugu1.

**Additional file 6: Figure S6**. The proportion of the large chromosome segments with genetics difference in the chromosome. Chr8, Chr9 and Chr3 had relatively high percentages.

**Additional file 7: Figure S7**. The nucleotide sequence alignment of AHAS from the genome of Huagu11 and Yugu1.

**Additional file 8: Figure S8**. The amino sequence alignment of AHASs from the cultivars with the different resistance to the imazethapyr.

**Additional file 9: Table S1**. The main different phenotypes of Yugu1 and Huagu11.

**Additional file 10: Table S2**. List of sequencing data generated.

**Additional file 11: Table S3**. The statistics of 17-mer analysis.

**Additional file 12: Table S4**. The statistics of Hi-C result.

**Additional file 13: Table S5**. Comparison of the assemblied genomes among the Huagu11, Yugu, Zhanggu and TT8.

**Additional file 14: Table S6**. Genome assembly completeness evaluation with BUSCO groups.

**Additional file 15: Table S7**. Mapping reads to the genome assembly.

**Additional file 16: Table S8**. Assessment of the Huagu11 genome assembly using 29399 EST sequences.

**Additional file 17: Table S9**. Repeat element in the foxtail millet genome.

**Additional file 18: Table S10**. Gene function annotation in the foxtail millet genome.

**Additional file 19: Table S11**. Non-coding RNA annotation in the foxtail millet genome.

**Additional file 20: Table S12**. Genomic rearrangement details between Huagu11 and Yugu1 genome.

**Additional file 21: Table S13**. Functional annotation of Huagu11 PAV spefific genes with Nr (20170924), KEGG (v89.1), Interpro (interproscan-5.30-69.0) and GO (gene_ontology.1_2) database.

**Additional file 22: Table S14**. Functional annotation of Yugu1 PAV spefific genes with Nr (20170924), KEGG (v89.1), Interpro (interproscan-5.30-69.0) and GO (gene_ontology.1_2) database.

**Additional file 23: Table S15**. Statistics of gene numbers in the different orthologue groups.

## Data Availability

The data that support the findings of this study have been deposited into CNGB Sequence Archive (CNSA) of China National GeneBank DataBase (CNGBdb) with accession number CNP0000993 (https://db.cngb.org/search/project/CNP0000993/).

## Abbreviations

Hi-C: High-throughput chromosome conformation capture
PAV: Presence/absence Variation
SNP: Single-nucleotide polymorphism
InDels: Insertion-deletion
AHAS: Acetohydroxy acid synthase
SMRT: Single-molecule real-time sequencing
BUSCO: Benchmarking Universal Single-Copy Orthologs
BWA: Burrows-Wheeler-Alignment
EST: Expressed Sequence Tag
LINEs: Long interspersed nuclear elements
SINEs: Short interspersed nuclear elements
miRNA: MicroRNA
snRNA: Small nuclear RNA
PCR: Polymerase chain reaction
GO: Gene Ontology
KEGG: Kyoto Encyclopedia of Genes and Genomes
