# Supplementary figures and images for: De novo genome assembly of a foxtail millet cultivar Huagu11 uncovered the genetic difference to the cultivar Yugu1, and the genetic mechanism of imazethapyr tolerance
Source: BMC Plant Biol. 2021 Jun 12;21:271. doi: 10.1186/s12870-021-03003-8 (PMC8196518; doi:10.1186/s12870-021-03003-8)

**
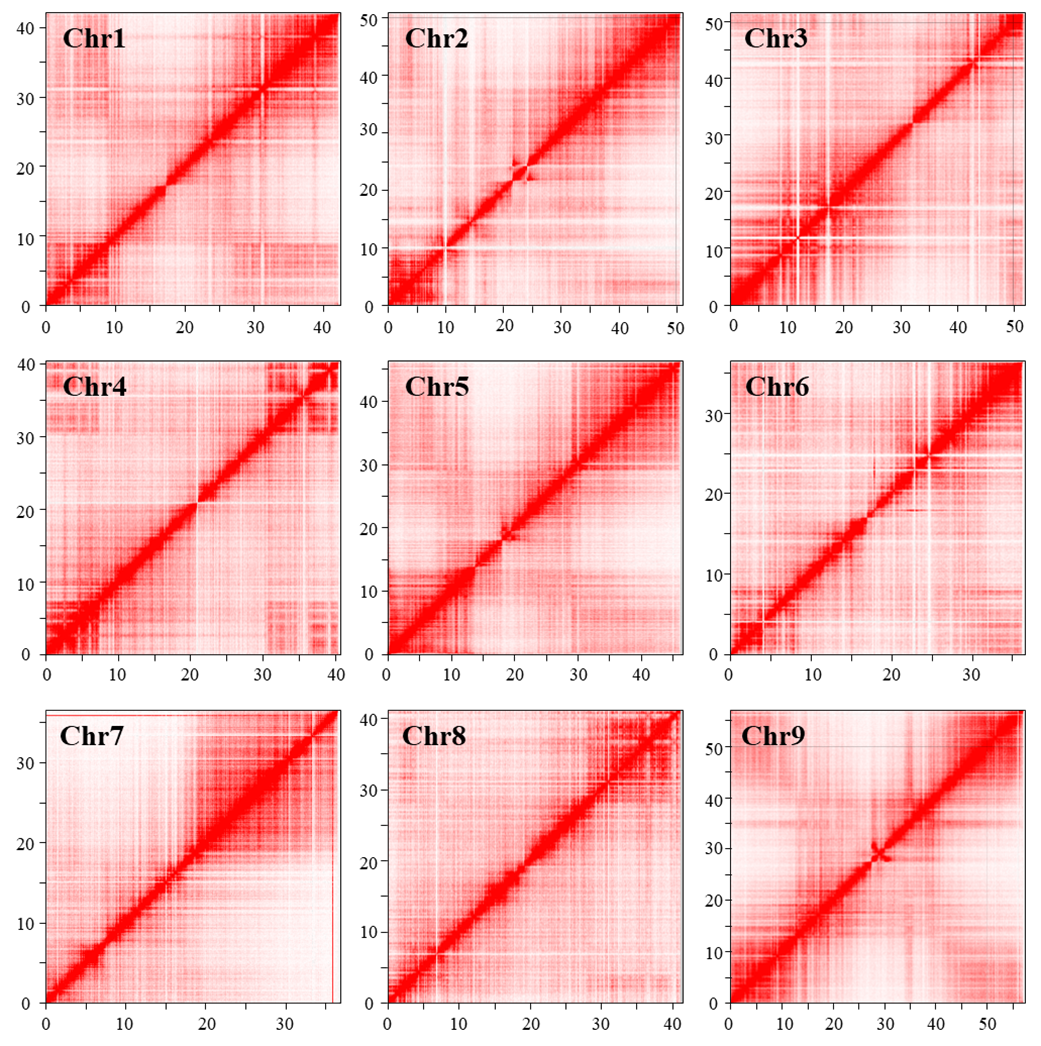
**

Figure S2. Hi C linkage density heat map of assembled contigs.

Supplement: Supplementary file 2 — Additional file 2: Figure S2. Hi C linkage density heat map of assembled contigs. [file 12870_2021_3003_MOESM2_ESM.docx]

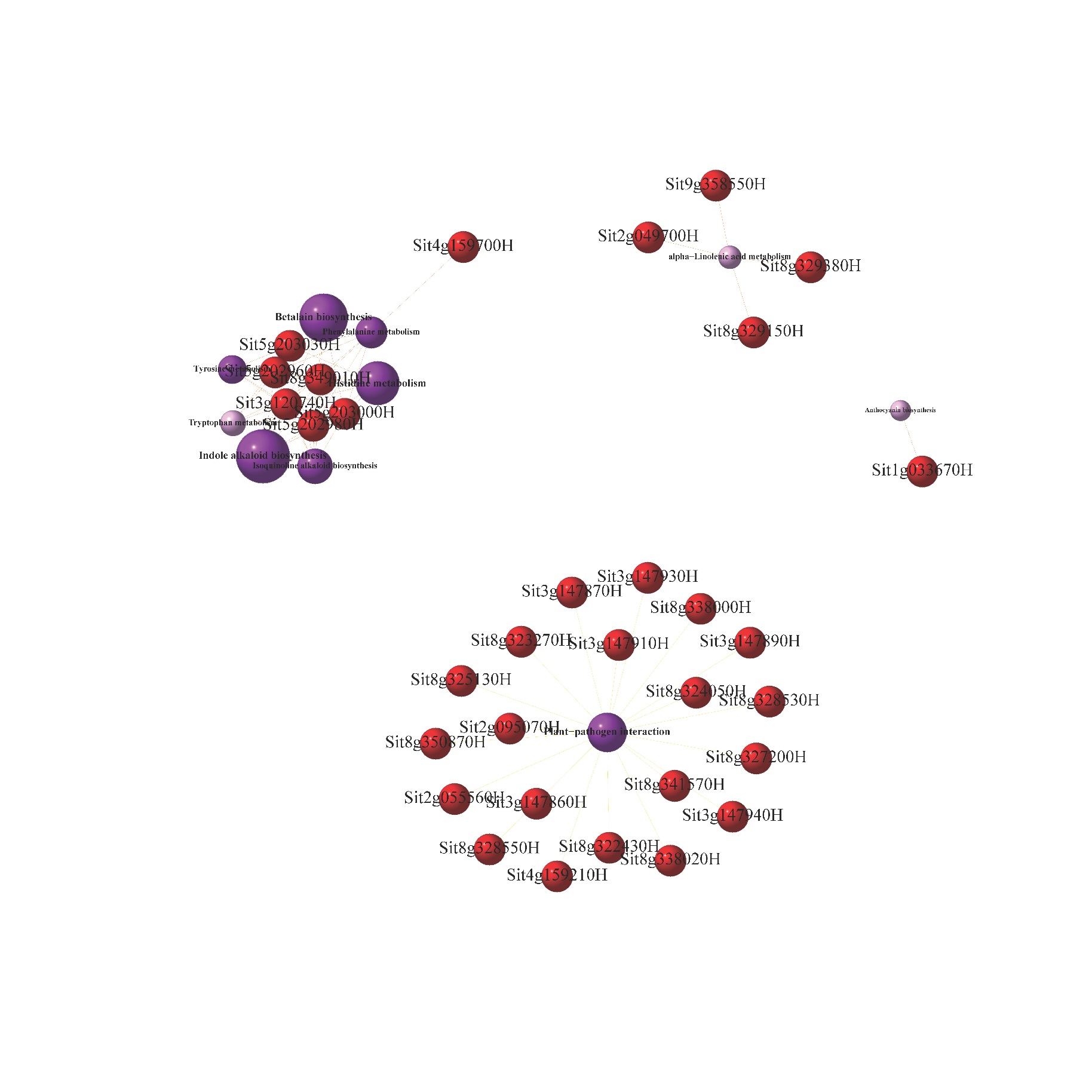


Figure S4. The enriched networks of the specific genes of Huagu11

Supplement: Supplementary file 4 — Additional file 4: Figure S4. The enriched networks of the specific genes of Huagu11. [file 12870_2021_3003_MOESM4_ESM.docx]

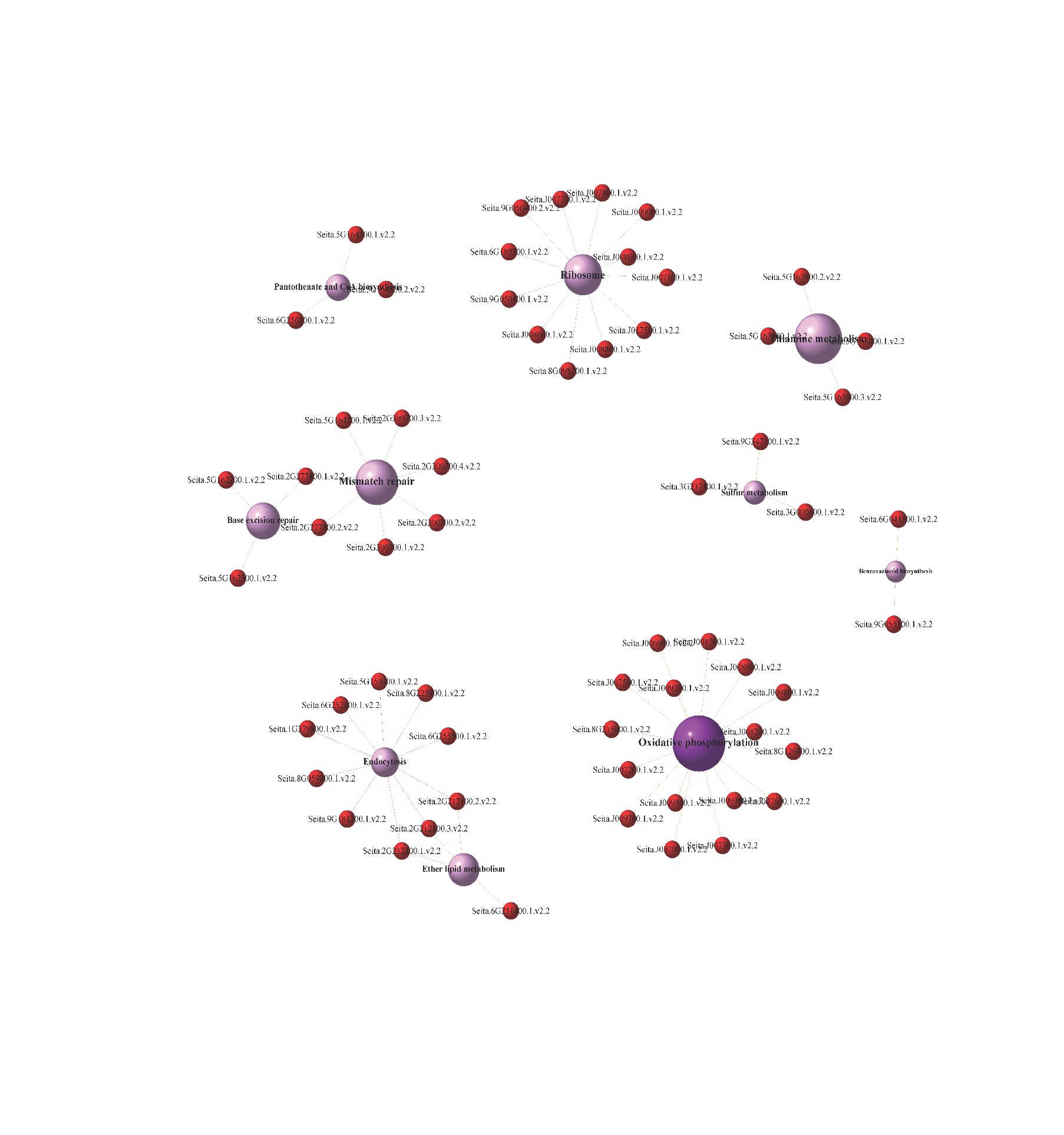


Figure S5. The enriched networks of the specific genes of Huagu11

Supplement: Supplementary file 5 — Additional file 5: Figure S5. The enriched networks of the specific genes of Yugu1. [file 12870_2021_3003_MOESM5_ESM.docx]

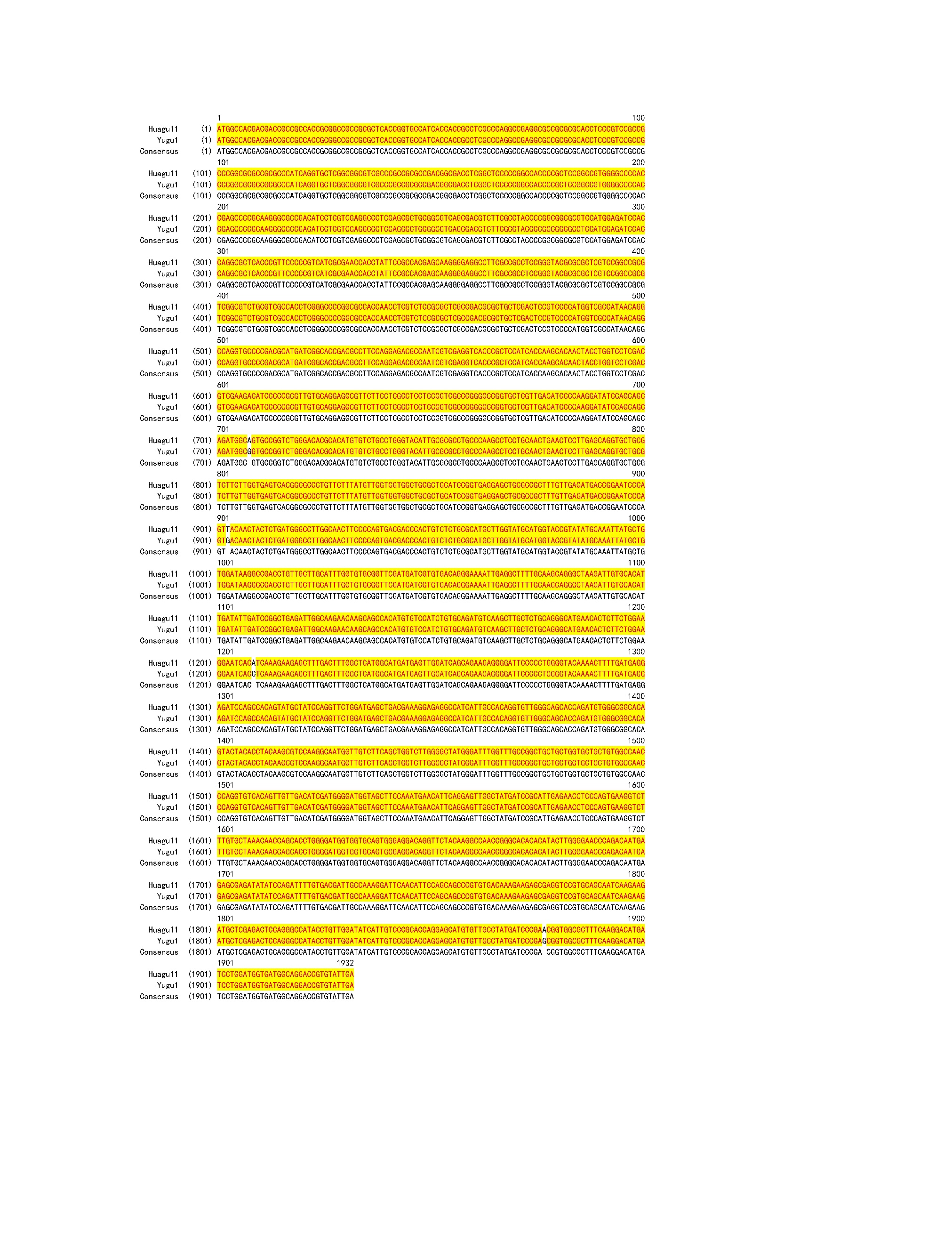


Figure S7. The nucleotide sequence alignment of *AHAS* from the genome of Huagu11 and Yugu1.

Supplement: Supplementary file 7 — Additional file 7: Figure S7. The nucleotide sequence alignment of AHAS from the genome of Huagu11 and Yugu1. [file 12870_2021_3003_MOESM7_ESM.docx]
