# Supplementary material for: De novo genome assembly of a foxtail millet cultivar Huagu11 uncovered the genetic difference to the cultivar Yugu1, and the genetic mechanism of imazethapyr tolerance
Source: BMC Plant Biol. 2021 Jun 12;21:271. doi: 10.1186/s12870-021-03003-8 (PMC8196518; doi:10.1186/s12870-021-03003-8)
Supplement: Supplementary file 3 — Additional file 3: Figure S3. Length distribution of PAV sequences between Huagu11 and Yugu1 genome. Most of the fragments were less than 5 Kb. [file 12870_2021_3003_MOESM3_ESM.docx]

Figure S3. Length distribution of PAV sequences between Huagu11 and Yugu1 genome. Most of the fragments were less than 5 Kb.
