# Supplementary material for: De novo genome assembly of a foxtail millet cultivar Huagu11 uncovered the genetic difference to the cultivar Yugu1, and the genetic mechanism of imazethapyr tolerance
Source: BMC Plant Biol. 2021 Jun 12;21:271. doi: 10.1186/s12870-021-03003-8 (PMC8196518; doi:10.1186/s12870-021-03003-8)
Supplement: Supplementary file 6 — Additional file 6: Figure S6. The proportion of the large chromosome segments with genetics difference in the chromosome. Chr8, Chr9 and Chr3 had relatively high percentages. [file 12870_2021_3003_MOESM6_ESM.docx]

Figure S6. The proportion of the large chromosome segments with genetics difference in the chromosome. Chr8, Chr9 and Chr3 had relatively high percentages.
