# Supplementary material for: De novo genome assembly of a foxtail millet cultivar Huagu11 uncovered the genetic difference to the cultivar Yugu1, and the genetic mechanism of imazethapyr tolerance
Source: BMC Plant Biol. 2021 Jun 12;21:271. doi: 10.1186/s12870-021-03003-8 (PMC8196518; doi:10.1186/s12870-021-03003-8)
Supplement: Supplementary file 8 — Additional file 8: Figure S8. The amino sequence alignment of AHASs from the cultivars with the different resistance to the imazethapyr. [file 12870_2021_3003_MOESM8_ESM.docx]

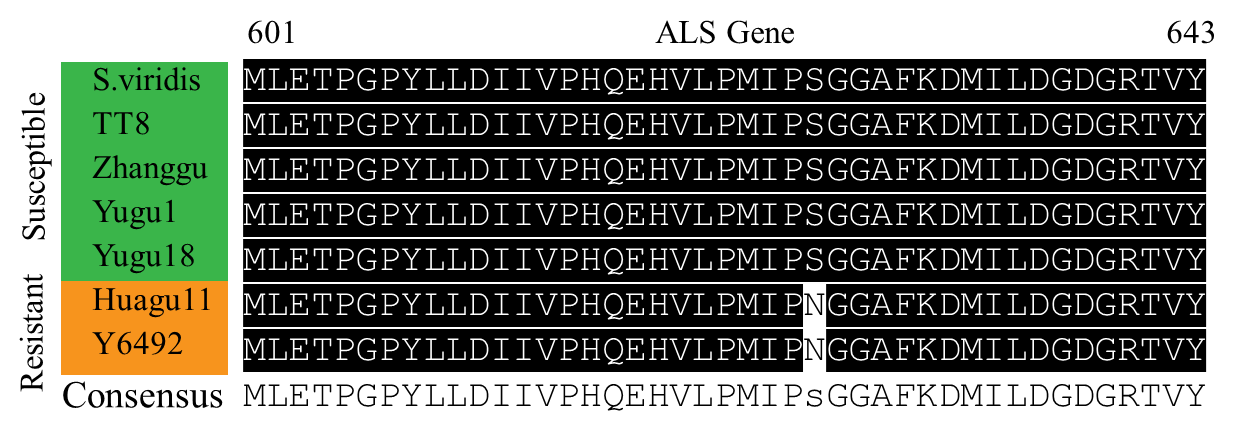


Figure S8 The amino sequence alignment of AHASs from the cultivars with the different resistance to the imazethapyr.
