# Supplementary material for: De novo genome assembly of a foxtail millet cultivar Huagu11 uncovered the genetic difference to the cultivar Yugu1, and the genetic mechanism of imazethapyr tolerance
Source: BMC Plant Biol. 2021 Jun 12;21:271. doi: 10.1186/s12870-021-03003-8 (PMC8196518; doi:10.1186/s12870-021-03003-8)
Supplement: Supplementary file 9 — Additional file 9: Table S1. The main different phenotypes of Yugu1 and Huagu11. [file 12870_2021_3003_MOESM9_ESM.docx]

| Cultivar | Grouting days | Plant length（cm） | Spike length（cm） | Internode length under panicle（cm） | Glume color | Thousand grain weight(g) | The response to the imazethapyr |
| --- | --- | --- | --- | --- | --- | --- | --- |
| Huagu11 | 37 | 122.67 | 17.67 | 12.67 | Purple | 2.86 | Resistance |
| Yugu1 | 39 | 138.67 | 18.33 | 13.33 | Kelly | 2.74 | Sensitive |

Table S1. The main different phenotypes of Yugu1 and Huagu11
