# Supplementary material for: De novo genome assembly of a foxtail millet cultivar Huagu11 uncovered the genetic difference to the cultivar Yugu1, and the genetic mechanism of imazethapyr tolerance
Source: BMC Plant Biol. 2021 Jun 12;21:271. doi: 10.1186/s12870-021-03003-8 (PMC8196518; doi:10.1186/s12870-021-03003-8)
Supplement: Supplementary file 10 — Additional file 10: Table S2. List of sequencing data generated. [file 12870_2021_3003_MOESM10_ESM.docx]

Table S2. List of sequencing data generated.

| Type | Library | Platform | Mean Fragment size(bp) | Read length (bp) | Raw data (Gp) | Raw coverage (x) | Effective data (Gp) | Effective coverage (x) |
| --- | --- | --- | --- | --- | --- | --- | --- | --- |
| Genome | PCR-pree | Illumina | 350 | 150_150 | 84.37 | 203.30 | 63.31 | 152.55 |
|  | Single molecule | PacBio | - | 8657* | 64.43 | 155.25 | 64.43 | 155.25 |
|  | Hi-C | Illumina | - | 100_100 | 132.57 | 319.45 | 34.34 | 82.75 |
| RNAseq | Root | Illumina | 200 | 100_100 | 10.48 |  | 10.25 |  |
|  | Steam | Illumina | 200 | 100_100 | 15.43 |  | 14.98 |  |
|  | Leaf | Illumina | 200 | 100_100 | 10.65 |  | 10.37 |  |
