# Supplementary material for: De novo genome assembly of a foxtail millet cultivar Huagu11 uncovered the genetic difference to the cultivar Yugu1, and the genetic mechanism of imazethapyr tolerance
Source: BMC Plant Biol. 2021 Jun 12;21:271. doi: 10.1186/s12870-021-03003-8 (PMC8196518; doi:10.1186/s12870-021-03003-8)
Supplement: Supplementary file 11 — Additional file 11: Table S3. The statistics of 17-mer analysis. [file 12870_2021_3003_MOESM11_ESM.docx]

Table S3. The statistics of 17-mer analysis.

| kmer | kmer_num | pkdepth | genome_size | used_base | used_read |
| --- | --- | --- | --- | --- | --- |
| 17 | 11,857,979,432 | 26 | 456,076,132 | 14,492,974,350 | 96,619,829 |
