# Supplementary material for: De novo genome assembly of a foxtail millet cultivar Huagu11 uncovered the genetic difference to the cultivar Yugu1, and the genetic mechanism of imazethapyr tolerance
Source: BMC Plant Biol. 2021 Jun 12;21:271. doi: 10.1186/s12870-021-03003-8 (PMC8196518; doi:10.1186/s12870-021-03003-8)
Supplement: Supplementary file 12 — Additional file 12: Table S4. The statistics of Hi-C result. [file 12870_2021_3003_MOESM12_ESM.docx]

Table S4. The statistics of Hi-C result.

| Chromosome name | Chromosome length | Chromosome length (%) | Anchor Congtig Number | Gene number | Gene percentage (%) |
| --- | --- | --- | --- | --- | --- |
| Chr1 | 42,287,314 | 10.35 | 15 | 4631 | 11.03 |
| Chr2 | 50,747,758 | 12.43 | 36 | 5426 | 12.92 |
| Chr3 | 51,699,307 | 12.66 | 19 | 5273 | 12.56 |
| Chr4 | 40,391,864 | 9.89 | 25 | 3824 | 9.11 |
| Chr5 | 46,281,728 | 11.33 | 19 | 5320 | 12.67 |
| Chr6 | 36,337,568 | 8.90 | 21 | 3280 | 7.81 |
| Chr7 | 36,536,947 | 8.95 | 12 | 3997 | 9.52 |
| Chr8 | 41,186,315 | 10.09 | 15 | 3340 | 7.95 |
| Chr9 | 57,179,044 | 14.00 | 19 | 6563 | 15.63 |
| Total | 402,647,845 | 98.60 | 181 | 41654 | 99.20 |
