# Supplementary material for: De novo genome assembly of a foxtail millet cultivar Huagu11 uncovered the genetic difference to the cultivar Yugu1, and the genetic mechanism of imazethapyr tolerance
Source: BMC Plant Biol. 2021 Jun 12;21:271. doi: 10.1186/s12870-021-03003-8 (PMC8196518; doi:10.1186/s12870-021-03003-8)
Supplement: Supplementary file 13 — Additional file 13: Table S5. Comparison of the assemblied genomes among the Huagu11, Yugu, Zhanggu and TT8. [file 12870_2021_3003_MOESM13_ESM.docx]

Table S5. Comparison of the assemblied genomes among the Huagu11, Yugu, Zhanggu and TT8

|  | Huagu11 | Yugu | Zhanggu | TT8 |
| --- | --- | --- | --- | --- |
| Scaffold Number | 215 | 336 | 3,555 | 2,689 |
| Total Scaffold Length | 408,372,916 | 405,732,883 | 417,479,101 | 477,541,705 |
| Max Scaffold Length | 58,425,682 | 58,970,307 | 4,589,508 | 65,039,919 |
| Mean Scaffold Length | 1,899,408 | 1,207,538 | 117,434 | 177,590 |
| Scaffold N50 (size/number) | 45,892,096/4 | 47,252,588/4 | 1,022,469/133 | 53,212,001/5 |
| Scaffold N90 (size/number) | 36,337,568/9 | 36,014,550/8 | 275,395/420 | 39,546,705/9 |
| N BaseNum | 82,000 | 4,823,979 | 29,202,168 | 13,670,722 |
| Contig Number | 379 | 6,778 | 37,837 | 18,089 |
| Total Contig Length | 408,290,916 | 400,908,904 | 388,276,933 | 463,870,983 |
| Max Contig Length | 20,505,576 | 585,239 | 216,717 | 1,710,213 |
| Mean Contig Length | 1,077,284 | 59,148 | 10,261 | 25,643 |
| Contig N50 (size/number) | 5,393,493/25 | 126,273/982 | 25,819/4,554 | 134,021/941 |
| Contig N90 (size/number) | 1,409,556/86 | 34,917/3251 | 6,024/15986 | 17,499/4315 |
| Chr length | 401,570,661 | 401,296,418 | 400,133,814 | 458,457,535 |
| GC% | 46.16% | 46.14% | 45.89% | 46.18% |
