# Supplementary material for: De novo genome assembly of a foxtail millet cultivar Huagu11 uncovered the genetic difference to the cultivar Yugu1, and the genetic mechanism of imazethapyr tolerance
Source: BMC Plant Biol. 2021 Jun 12;21:271. doi: 10.1186/s12870-021-03003-8 (PMC8196518; doi:10.1186/s12870-021-03003-8)
Supplement: Supplementary file 14 — Additional file 14: Table S6. Genome assembly completeness evaluation with BUSCO groups. [file 12870_2021_3003_MOESM14_ESM.docx]

Table S6. Genome assembly completeness evaluation with BUSCO groups.

|  | Huagu11 | | Yugu | | Zhanggu | | TT8 | |
| --- | --- | --- | --- | --- | --- | --- | --- | --- |
| Total searched:1375 | Number | % | Number | % | Number | % | Number | % |
| Complete | 1351 | 98.3 | 1345 | 97.8 | 1342 | 97.6 | 1341 | 97.5 |
| Complete single-copy | 1328 | 96.6 | 1323 | 96.2 | 1313 | 95.5 | 1239 | 90.1 |
| Complete duplicated | 23 | 1.7 | 22 | 1.6 | 29 | 2.1 | 102 | 7.4 |
| Fragmented | 6 | 0.4 | 9 | 0.7 | 10 | 0.7 | 8 | 0.6 |
| Missing | 18 | 1.3 | 21 | 1.5 | 23 | 1.7 | 26 | 1.9 |
