# Supplementary material for: De novo genome assembly of a foxtail millet cultivar Huagu11 uncovered the genetic difference to the cultivar Yugu1, and the genetic mechanism of imazethapyr tolerance
Source: BMC Plant Biol. 2021 Jun 12;21:271. doi: 10.1186/s12870-021-03003-8 (PMC8196518; doi:10.1186/s12870-021-03003-8)
Supplement: Supplementary file 15 — Additional file 15: Table S7. Mapping reads to the genome assembly. [file 12870_2021_3003_MOESM15_ESM.docx]

Table S7. Mapping reads to the genome assembly.

| Reads | Average sequencing depth | 152 |
| --- | --- | --- |
|  | Mapping rate | 98.30% |
| Genome | Coverage | 99.12% |
|  | Coverage at least 5× | 98.60% |
|  | Coverage at least 10× | 98.29% |
|  | Coverage at least 20× | 97.75% |
|  | Coverage at least 40× | 96.59% |
