# Supplementary material for: De novo genome assembly of a foxtail millet cultivar Huagu11 uncovered the genetic difference to the cultivar Yugu1, and the genetic mechanism of imazethapyr tolerance
Source: BMC Plant Biol. 2021 Jun 12;21:271. doi: 10.1186/s12870-021-03003-8 (PMC8196518; doi:10.1186/s12870-021-03003-8)
Supplement: Supplementary file 16 — Additional file 16: Table S8. Assessment of the Huagu11 genome assembly using 29399 EST sequences. [file 12870_2021_3003_MOESM16_ESM.docx]

Table S8. Assessment of the Huagu11 genome assembly using 29399 EST sequences.

| Dataset | Number | Total length(bp) | Bases covered by assembly (%) | with >90% sequence in one scaffold | | with >50% sequence in one scaffold | |
| --- | --- | --- | --- | --- | --- | --- | --- |
|  |  |  |  | Number | Percent | Number | Percent |
| >0bp | 29,399 | 16,633,380 | 97.37 | 27,628 | 93.98 | 28,553 | 97.12 |
| >200bp | 28,457 | 16,496,108 | 97.55 | 27,038 | 95.01 | 27,833 | 97.81 |
| >500bp | 19,441 | 12,850,583 | 97.86 | 18,636 | 95.86 | 19,096 | 98.23 |
