# Supplementary material for: De novo genome assembly of a foxtail millet cultivar Huagu11 uncovered the genetic difference to the cultivar Yugu1, and the genetic mechanism of imazethapyr tolerance
Source: BMC Plant Biol. 2021 Jun 12;21:271. doi: 10.1186/s12870-021-03003-8 (PMC8196518; doi:10.1186/s12870-021-03003-8)
Supplement: Supplementary file 17 — Additional file 17: Table S9. Repeat element in the foxtail millet genome. [file 12870_2021_3003_MOESM17_ESM.docx]

Table S9. Repeat element in the foxtail millet genome.

| Type | Repbase TEs | | TE protiens | | De novo | | Cobpined TEs | |
| --- | --- | --- | --- | --- | --- | --- | --- | --- |
|  | Length (bp) | % in genome | Length (bp) | % in genome | Length (bp) | % in genome | Length (bp) | % in genome |
| DNA | 28,002,958 | 6.86 | 6,651,493 | 1.63 | 32,823,118 | 8.04 | 47,088,377 | 11.53 |
| LINE | 5,643,366 | 1.38 | 4,397,694 | 1.08 | 6,135,172 | 1.50 | 10,044,519 | 2.46 |
| SINE | 54,428 | 0.01 | 0 | 0.00 | 485,733 | 0.12 | 517,744 | 0.13 |
| LTR | 66,550,147 | 16.30 | 36,538,541 | 8.95 | 122,369,176 | 29.97 | 130,587,841 | 31.98 |
| Other | 2,770 | 0.00 | 0 | 0.00 | 0 | 0.00 | 2,770 | 0.00 |
| Unknown | 0 | 0.00 | 0 | 0.00 | 3,395,021 | 0.83 | 3,395,021 | 0.83 |
| Total | 100,124,266 | 24.52 | 47,585,934 | 11.65 | 162,277,711 | 39.75 | 182,228,354 | 44.63 |
