# Supplementary material for: De novo genome assembly of a foxtail millet cultivar Huagu11 uncovered the genetic difference to the cultivar Yugu1, and the genetic mechanism of imazethapyr tolerance
Source: BMC Plant Biol. 2021 Jun 12;21:271. doi: 10.1186/s12870-021-03003-8 (PMC8196518; doi:10.1186/s12870-021-03003-8)
Supplement: Supplementary file 18 — Additional file 18: Table S10. Gene function annotation in the foxtail millet genome. [file 12870_2021_3003_MOESM18_ESM.docx]

Table S10. Gene function annotation in the foxtail millet genome

| Values | Total | Nr-Annotated | Swissprot-Annotated | KEGG-Annotated | TrEMBL-Annotated | Interpro-Annotated | GO-Annotated | Overall |
| --- | --- | --- | --- | --- | --- | --- | --- | --- |
| Number | 41,992 | 33,878 | 22,825 | 22,126 | 34,589 | 23,941 | 17,269 | 35,177 |
| Percentage | 100% | 80.68% | 54.36% | 52.69% | 82.37% | 57.01% | 41.12% | 83.77% |
