# Supplementary material for: De novo genome assembly of a foxtail millet cultivar Huagu11 uncovered the genetic difference to the cultivar Yugu1, and the genetic mechanism of imazethapyr tolerance
Source: BMC Plant Biol. 2021 Jun 12;21:271. doi: 10.1186/s12870-021-03003-8 (PMC8196518; doi:10.1186/s12870-021-03003-8)
Supplement: Supplementary file 19 — Additional file 19: Table S11. Non-coding RNA annotation in the foxtail millet genome. [file 12870_2021_3003_MOESM19_ESM.docx]

Table S11. Non-coding RNA annotation in the foxtail millet genome

| Type | | Copy(w) | Average length(bp) | Total length(bp) | % of genome |
| --- | --- | --- | --- | --- | --- |
| miRNA | | 161 | 133.07 | 21,424 | 0.0052 |
| tRNA | | 976 | 75.09 | 73,284 | 0.0179 |
| rRNA | rRNA | 91 | 118.30 | 10,765 | 0.0026 |
|  | 18S | 3 | 243.33 | 730 | 0.0002 |
|  | 28S | 1 | 77.00 | 77 | 0.0000 |
|  | 5.8S | 0 | 0.00 | 0 | 0.0000 |
|  | 5S | 87 | 114.46 | 9,958 | 0.0024 |
| snRNA | snRNA | 503 | 117.72 | 59,214 | 0.0145 |
|  | CD-box | 352 | 107.15 | 37,716 | 0.0092 |
|  | HACA-box | 59 | 128.39 | 7,575 | 0.0019 |
|  | splicing | 92 | 151.34 | 13,923 | 0.0034 |
