# Supplementary material for: De novo genome assembly of a foxtail millet cultivar Huagu11 uncovered the genetic difference to the cultivar Yugu1, and the genetic mechanism of imazethapyr tolerance
Source: BMC Plant Biol. 2021 Jun 12;21:271. doi: 10.1186/s12870-021-03003-8 (PMC8196518; doi:10.1186/s12870-021-03003-8)
Supplement: Supplementary file 20 — Additional file 20: Table S12. Genomic rearrangement details between Huagu11 and Yugu1 genome. [file 12870_2021_3003_MOESM20_ESM.docx]

Table S12. Genomic rearrangement details between Huagu11 and Yugu1 genome

| Huagu11 | Start | End | Yugu1 | Start | End | Type |
| --- | --- | --- | --- | --- | --- | --- |
| Chr1 | 333746 | 334953 | chr9 | 31104807 | 31106036 | Inter-translocation |
| Chr1 | 336262 | 334990 | chr4 | 4917015 | 4918288 | Inversion |
| Chr1 | 672749 | 674131 | chr3 | 22869234 | 22870635 | Inter-translocation |
| Chr1 | 725587 | 725419 | chr8 | 39736546 | 39736714 | Inversion |
| Chr1 | 725835 | 725591 | chr8 | 39735604 | 39735852 | Inversion |
| Chr1 | 773094 | 771767 | chr8 | 20187992 | 20189320 | Inversion |
| Chr1 | 816903 | 817184 | chr8 | 13590838 | 13591119 | Inter-translocation |
| Chr1 | 952776 | 942747 | chr1 | 803972 | 813838 | Inversion |
| Chr1 | 956144 | 954109 | chr1 | 860116 | 862144 | Inversion |
| Chr1 | 966654 | 964396 | chr1 | 814195 | 816513 | Inversion |
| Chr1 | 1077616 | 1083001 | chr9 | 29159634 | 29165041 | Inter-translocation |
| Chr1 | 1488413 | 1488769 | chr5 | 6983989 | 6984345 | Inter-translocation |
| Chr1 | 1547030 | 1546790 | chr1 | 402083 | 402323 | Inversion |
| Chr1 | 2290402 | 2290062 | chr8 | 38753579 | 38753920 | Inversion |
| Chr1 | 2391473 | 2391825 | chr4 | 5592637 | 5592991 | Inter-translocation |
| Chr1 | 2466844 | 2466646 | chr9 | 57114483 | 57114681 | Inversion |
| Chr1 | 2788373 | 2789414 | chr8 | 2772654 | 2773685 | Inter-translocation |
| Chr1 | 2854115 | 2859537 | chr1 | 2797014 | 2802441 | Intra-translocation |
| Chr1 | 2868031 | 2867438 | chr8 | 7916053 | 7916636 | Inversion |
| Chr1 | 2873765 | 2874117 | chr4 | 7446946 | 7447298 | Inter-translocation |
| Chr1 | 3085186 | 3084834 | chr3 | 48138169 | 48138521 | Inversion |
| Chr1 | 3250157 | 3250540 | chr8 | 9250139 | 9250522 | Inter-translocation |
| Chr1 | 3915898 | 3914476 | chr8 | 10390136 | 10391559 | Inversion |
| Chr1 | 4028892 | 4029173 | chr5 | 8445980 | 8446261 | Inter-translocation |
| Chr1 | 4491782 | 4489511 | chr5 | 14940170 | 14942471 | Inversion |
| Chr1 | 5168309 | 5165338 | chr3 | 39641246 | 39644212 | Inversion |
| Chr1 | 5241440 | 5244373 | chr9 | 16801286 | 16804223 | Inter-translocation |
| Chr1 | 5254532 | 5254888 | chr4 | 5908661 | 5909017 | Inter-translocation |
| Chr1 | 6999419 | 6998460 | chr1 | 24380042 | 24381000 | Inversion |
| Chr1 | 7680474 | 7683842 | chr1 | 10495384 | 10498751 | Intra-translocation |
| Chr1 | 7986758 | 7986416 | chr3 | 21612032 | 21612376 | Inversion |
| Chr1 | 8610726 | 8611081 | chr3 | 44527228 | 44527583 | Inter-translocation |
| Chr1 | 8902583 | 8902432 | chr8 | 5880596 | 5880747 | Inversion |
| Chr1 | 9223510 | 9215349 | chr9 | 28398401 | 28406514 | Inversion |
| Chr1 | 9387691 | 9385475 | chr8 | 20036455 | 20038699 | Inversion |
| Chr1 | 9639113 | 9638934 | chr8 | 29828586 | 29828765 | Inversion |
| Chr1 | 9669669 | 9669749 | chr3 | 11406675 | 11406755 | Inter-translocation |
| Chr1 | 9670221 | 9670865 | chr8 | 38173380 | 38174029 | Inter-translocation |
| Chr1 | 10523593 | 10523420 | chr8 | 8157034 | 8157207 | Inversion |
| Chr1 | 10542890 | 10541620 | chr8 | 27652352 | 27653618 | Inversion |
| Chr1 | 10543690 | 10543135 | chr8 | 27824327 | 27824882 | Inversion |
| Chr1 | 10550204 | 10547363 | chr8 | 27817292 | 27820131 | Inversion |
| Chr1 | 10645241 | 10645517 | chr3 | 2911364 | 2911640 | Inter-translocation |
| Chr1 | 10752751 | 10751344 | chr9 | 27226884 | 27228291 | Inversion |
| Chr1 | 10922473 | 10922743 | chr6 | 34226465 | 34226721 | Inter-translocation |
| Chr1 | 11190633 | 11193808 | chr1 | 27622537 | 27625703 | Intra-translocation |
| Chr1 | 11228704 | 11227033 | chr8 | 3400090 | 3401757 | Inversion |
| Chr1 | 11236005 | 11231515 | chr8 | 3392649 | 3397127 | Inversion |
| Chr1 | 12039384 | 12036527 | chr9 | 41746768 | 41749607 | Inversion |
| Chr1 | 12505308 | 12505899 | chr4 | 2369869 | 2370432 | Inter-translocation |
| Chr1 | 12717217 | 12717562 | chr3 | 44326721 | 44327068 | Inter-translocation |
| Chr1 | 12815022 | 12814934 | chr8 | 25195094 | 25195182 | Inversion |
| Chr1 | 12912290 | 12912046 | chr6 | 28301626 | 28301868 | Inversion |
| Chr1 | 13545478 | 13547604 | chr9 | 31062180 | 31064306 | Inter-translocation |
| Chr1 | 13997469 | 13997586 | chr7 | 4475104 | 4475221 | Inter-translocation |
| Chr1 | 14731837 | 14734146 | chr8 | 37679842 | 37682151 | Inter-translocation |
| Chr1 | 14756960 | 14756449 | chr8 | 27480988 | 27481499 | Inversion |
| Chr1 | 15446529 | 15446182 | chr2 | 43716208 | 43716555 | Inversion |
| Chr1 | 15667349 | 15661781 | chr9 | 26440605 | 26446180 | Inversion |
| Chr1 | 15748539 | 15751016 | chr8 | 25179640 | 25182088 | Inter-translocation |
| Chr1 | 17513360 | 17513252 | chr1 | 17268218 | 17268325 | Inversion |
| Chr1 | 17952758 | 17893575 | chr1 | 19590220 | 19649410 | Inversion |
| Chr1 | 17981095 | 17953218 | chr1 | 19561523 | 19589357 | Inversion |
| Chr1 | 18288753 | 17981828 | chr1 | 19254600 | 19561536 | Inversion |
| Chr1 | 18393820 | 18288990 | chr1 | 19149669 | 19254499 | Inversion |
| Chr1 | 18504406 | 18394671 | chr1 | 19036763 | 19146499 | Inversion |
| Chr1 | 18635418 | 18504799 | chr1 | 18906153 | 19036767 | Inversion |
| Chr1 | 18677968 | 18635757 | chr1 | 18863843 | 18906052 | Inversion |
| Chr1 | 18802158 | 18678680 | chr1 | 18740273 | 18863742 | Inversion |
| Chr1 | 18927730 | 18808988 | chr1 | 18616127 | 18734845 | Inversion |
| Chr1 | 18934633 | 18927836 | chr1 | 18609231 | 18616023 | Inversion |
| Chr1 | 18967320 | 18934803 | chr1 | 18576555 | 18609070 | Inversion |
| Chr1 | 19065267 | 19067753 | chr9 | 29290788 | 29293274 | Inter-translocation |
| Chr1 | 19066143 | 18967560 | chr1 | 18477723 | 18576309 | Inversion |
| Chr1 | 19245010 | 19067716 | chr1 | 18299494 | 18476770 | Inversion |
| Chr1 | 19298518 | 19254692 | chr1 | 18271685 | 18315515 | Inversion |
| Chr1 | 19341068 | 19298534 | chr1 | 18228807 | 18271338 | Inversion |
| Chr1 | 19385710 | 19341195 | chr1 | 18184198 | 18228706 | Inversion |
| Chr1 | 19487716 | 19499777 | chr1 | 13268208 | 13280269 | Intra-translocation |
| Chr1 | 19490216 | 19386233 | chr1 | 18080215 | 18184201 | Inversion |
| Chr1 | 19533300 | 19500036 | chr1 | 18038971 | 18072242 | Inversion |
| Chr1 | 19620302 | 19533550 | chr1 | 17952120 | 18038870 | Inversion |
| Chr1 | 19668970 | 19614134 | chr1 | 17905221 | 17960051 | Inversion |
| Chr1 | 19705982 | 19669471 | chr1 | 17836002 | 17872522 | Inversion |
| Chr1 | 19715785 | 19706036 | chr1 | 17789036 | 17798784 | Inversion |
| Chr1 | 19740903 | 19712297 | chr1 | 17808785 | 17837392 | Inversion |
| Chr1 | 19760315 | 19762012 | chr8 | 7614808 | 7616504 | Inter-translocation |
| Chr1 | 19772734 | 19771407 | chr1 | 17758806 | 17760132 | Inversion |
| Chr1 | 19883091 | 19772757 | chr1 | 17648325 | 17758659 | Inversion |
| Chr1 | 20478464 | 20471766 | chr9 | 33943422 | 33950155 | Inversion |
| Chr1 | 20768083 | 20768408 | chr7 | 33410381 | 33410706 | Inter-translocation |
| Chr1 | 21346007 | 21345117 | chr9 | 29204552 | 29205442 | Inversion |
| Chr1 | 21674993 | 21674720 | chr1 | 13173353 | 13173629 | Inversion |
| Chr1 | 22269939 | 22263002 | chr8 | 10030551 | 10037460 | Inversion |
| Chr1 | 22292023 | 22289068 | chr5 | 15980606 | 15983561 | Inversion |
| Chr1 | 22506417 | 22506731 | chr9 | 32873571 | 32873881 | Inter-translocation |
| Chr1 | 23774982 | 23780095 | chr1 | 23498157 | 23503271 | Intra-translocation |
| Chr1 | 24331018 | 24330657 | chr8 | 39666111 | 39666471 | Inversion |
| Chr1 | 24339713 | 24339170 | chr5 | 14879489 | 14880020 | Inversion |
| Chr1 | 24447920 | 24438228 | chr1 | 25321285 | 25330984 | Inversion |
| Chr1 | 24487269 | 24483859 | chr6 | 11939986 | 11943382 | Inversion |
| Chr1 | 24487349 | 24492166 | chr8 | 8650481 | 8655296 | Inter-translocation |
| Chr1 | 24706640 | 24706981 | chr9 | 40322038 | 40322377 | Inter-translocation |
| Chr1 | 24892141 | 24890618 | chr9 | 25884540 | 25886064 | Inversion |
| Chr1 | 24906262 | 24907124 | chr1 | 24580037 | 24580899 | Intra-translocation |
| Chr1 | 25019690 | 25020055 | chr9 | 13563393 | 13563758 | Inter-translocation |
| Chr1 | 25108695 | 25108470 | chr8 | 5866992 | 5867217 | Inversion |
| Chr1 | 25321443 | 25317751 | chr8 | 22753286 | 22756974 | Inversion |
| Chr1 | 25376798 | 25378364 | chr9 | 27810628 | 27812196 | Inter-translocation |
| Chr1 | 25564109 | 25561929 | chr5 | 16178701 | 16180895 | Inversion |
| Chr1 | 25591058 | 25587002 | chr8 | 38362760 | 38366803 | Inversion |
| Chr1 | 25592332 | 25594846 | chr8 | 11192753 | 11195267 | Inter-translocation |
| Chr1 | 25724889 | 25721659 | chr5 | 15588076 | 15591306 | Inversion |
| Chr1 | 25792619 | 25792963 | chr4 | 37632966 | 37633310 | Inter-translocation |
| Chr1 | 25848814 | 25848928 | chr3 | 14696557 | 14696671 | Inter-translocation |
| Chr1 | 26189674 | 26189430 | chr9 | 23957311 | 23957554 | Inversion |
| Chr1 | 26268182 | 26269577 | chr9 | 31123904 | 31125266 | Inter-translocation |
| Chr1 | 26276572 | 26276835 | chr7 | 30474387 | 30474658 | Inter-translocation |
| Chr1 | 26279321 | 26278907 | chr4 | 10292895 | 10293311 | Inversion |
| Chr1 | 26409635 | 26407703 | chr8 | 8618363 | 8620293 | Inversion |
| Chr1 | 26502648 | 26502888 | chr1 | 26603458 | 26603698 | Intra-translocation |
| Chr1 | 26502670 | 26502434 | chr8 | 11504845 | 11505081 | Inversion |
| Chr1 | 26588762 | 26592218 | chr3 | 13938699 | 13942141 | Inter-translocation |
| Chr1 | 26627425 | 26625987 | chr8 | 9928034 | 9929450 | Inversion |
| Chr1 | 26627427 | 26626645 | chr8 | 11996571 | 11997358 | Inversion |
| Chr1 | 26765896 | 26766768 | chr5 | 15796987 | 15797856 | Inter-translocation |
| Chr1 | 27042139 | 27038969 | chr1 | 27622536 | 27625701 | Inversion |
| Chr1 | 27065278 | 27065166 | chr7 | 30725930 | 30726042 | Inversion |
| Chr1 | 27097199 | 27097560 | chr7 | 33367164 | 33367525 | Inter-translocation |
| Chr1 | 27137836 | 27141535 | chr5 | 15459033 | 15462736 | Inter-translocation |
| Chr1 | 27960417 | 27960651 | chr9 | 21507736 | 21507970 | Inter-translocation |
| Chr1 | 28601133 | 28600963 | chr9 | 41897314 | 41897484 | Inversion |
| Chr1 | 31040151 | 31043124 | chr3 | 10802909 | 10805881 | Inter-translocation |
| Chr1 | 31673475 | 31672907 | chr4 | 9208774 | 9209342 | Inversion |
| Chr1 | 32046508 | 32046424 | chr3 | 28091616 | 28091700 | Inversion |
| Chr1 | 32046542 | 32046427 | chr1 | 25424098 | 25424213 | Inversion |
| Chr1 | 32277294 | 32279823 | chr1 | 32066455 | 32068970 | Intra-translocation |
| Chr1 | 32279850 | 32281633 | chr1 | 32069088 | 32070872 | Intra-translocation |
| Chr1 | 34820457 | 34820381 | chr7 | 31783822 | 31783898 | Inversion |
| Chr1 | 36111656 | 36111943 | chr5 | 8640108 | 8640395 | Inter-translocation |
| Chr1 | 36932651 | 36931570 | chr3 | 6981331 | 6982412 | Inversion |
| Chr1 | 36936309 | 36937115 | chr3 | 6982411 | 6983216 | Inter-translocation |
| Chr1 | 36959824 | 36951268 | chr9 | 16801977 | 16810540 | Inversion |
| Chr1 | 38669046 | 38674094 | chr9 | 12133733 | 12138788 | Inter-translocation |
| Chr1 | 40031154 | 40030863 | chr9 | 49632692 | 49632983 | Inversion |
| Chr1 | 41247416 | 41247258 | chr1 | 41297898 | 41298055 | Inversion |
| Chr1 | 41291817 | 41292091 | chr4 | 9966342 | 9966616 | Inter-translocation |
| Chr2 | 32192 | 32395 | chr3 | 25088857 | 25089068 | Inter-translocation |
| Chr2 | 70564 | 70074 | chr7 | 35083670 | 35084159 | Inversion |
| Chr2 | 75017 | 70563 | chr6 | 33683005 | 33687465 | Inversion |
| Chr2 | 88392 | 88737 | chr6 | 28614775 | 28615120 | Inter-translocation |
| Chr2 | 251834 | 252688 | chr9 | 30002326 | 30003167 | Inter-translocation |
| Chr2 | 282174 | 282240 | chr2 | 2983756 | 2983822 | Intra-translocation |
| Chr2 | 294447 | 293996 | chr8 | 12739129 | 12739580 | Inversion |
| Chr2 | 294996 | 294417 | chr5 | 9586225 | 9586807 | Inversion |
| Chr2 | 1119877 | 1122809 | chr2 | 787108 | 790040 | Intra-translocation |
| Chr2 | 1143083 | 1142739 | chr9 | 28857412 | 28857754 | Inversion |
| Chr2 | 1722545 | 1721092 | chr8 | 14331796 | 14333202 | Inversion |
| Chr2 | 2643812 | 2642524 | chr6 | 1377411 | 1378701 | Inversion |
| Chr2 | 2888636 | 2888325 | chr9 | 31742297 | 31742607 | Inversion |
| Chr2 | 2901206 | 2901966 | chr2 | 2562533 | 2563254 | Intra-translocation |
| Chr2 | 2907431 | 2906842 | chr2 | 2568551 | 2569135 | Inversion |
| Chr2 | 2918507 | 2919973 | chr2 | 2575976 | 2577441 | Intra-translocation |
| Chr2 | 3021853 | 3021501 | chr9 | 40409679 | 40410031 | Inversion |
| Chr2 | 3221646 | 3221580 | chr3 | 28091556 | 28091622 | Inversion |
| Chr2 | 3309589 | 3307082 | chr9 | 18218999 | 18221512 | Inversion |
| Chr2 | 3515729 | 3514892 | chr4 | 33210547 | 33211384 | Inversion |
| Chr2 | 3601794 | 3600272 | chr2 | 3358545 | 3360071 | Inversion |
| Chr2 | 3612755 | 3612900 | chr2 | 3290177 | 3290322 | Intra-translocation |
| Chr2 | 3613995 | 3614490 | chr2 | 3311121 | 3311610 | Intra-translocation |
| Chr2 | 3615903 | 3616382 | chr2 | 3290911 | 3291400 | Intra-translocation |
| Chr2 | 3617137 | 3617552 | chr2 | 3313651 | 3314065 | Intra-translocation |
| Chr2 | 3617681 | 3617552 | chr8 | 7540891 | 7541020 | Inversion |
| Chr2 | 3627313 | 3627494 | chr2 | 3327978 | 3328157 | Intra-translocation |
| Chr2 | 3908584 | 3908747 | chr4 | 33154548 | 33154712 | Inter-translocation |
| Chr2 | 3965121 | 3965427 | chr9 | 540816 | 541128 | Inter-translocation |
| Chr2 | 3992716 | 3992141 | chr8 | 7820572 | 7821175 | Inversion |
| Chr2 | 4120192 | 4120432 | chr8 | 13818053 | 13818290 | Inter-translocation |
| Chr2 | 4189493 | 4192072 | chr3 | 24036172 | 24038703 | Inter-translocation |
| Chr2 | 4295881 | 4296607 | chr2 | 4043887 | 4044609 | Intra-translocation |
| Chr2 | 4298230 | 4300000 | chr2 | 4044607 | 4046354 | Intra-translocation |
| Chr2 | 4316067 | 4315946 | chr5 | 15050753 | 15050871 | Inversion |
| Chr2 | 4318749 | 4318568 | chr4 | 900854 | 901035 | Inversion |
| Chr2 | 4325813 | 4325937 | chr7 | 29865978 | 29866102 | Inter-translocation |
| Chr2 | 4348157 | 4348432 | chr8 | 5249165 | 5249440 | Inter-translocation |
| Chr2 | 4699136 | 4698505 | chr5 | 18205582 | 18206209 | Inversion |
| Chr2 | 4809128 | 4809273 | chr6 | 4017614 | 4017759 | Inter-translocation |
| Chr2 | 4868682 | 4863600 | chr6 | 3191509 | 3196581 | Inversion |
| Chr2 | 5228691 | 5227652 | chr3 | 45831477 | 45832495 | Inversion |
| Chr2 | 5893130 | 5890253 | chr1 | 25276248 | 25279159 | Inversion |
| Chr2 | 5925137 | 5926685 | chr2 | 5661900 | 5663446 | Intra-translocation |
| Chr2 | 6169483 | 6181345 | chr2 | 5859198 | 5871057 | Intra-translocation |
| Chr2 | 6567679 | 6566908 | chr8 | 16651231 | 16652001 | Inversion |
| Chr2 | 7139146 | 7144728 | chr3 | 22445558 | 22451191 | Inter-translocation |
| Chr2 | 7706383 | 7706027 | chr4 | 7034175 | 7034531 | Inversion |
| Chr2 | 8181219 | 8180873 | chr9 | 57415744 | 57416090 | Inversion |
| Chr2 | 8328055 | 8327867 | chr8 | 5778557 | 5778747 | Inversion |
| Chr2 | 8332390 | 8328086 | chr9 | 34543843 | 34548143 | Inversion |
| Chr2 | 8464963 | 8463717 | chr2 | 8144463 | 8145706 | Inversion |
| Chr2 | 9727761 | 9752690 | chr2 | 9561065 | 9585994 | Intra-translocation |
| Chr2 | 9798070 | 9789018 | chr2 | 9470374 | 9479424 | Inversion |
| Chr2 | 9844710 | 9798112 | chr2 | 9423816 | 9470413 | Inversion |
| Chr2 | 9851119 | 9844752 | chr2 | 9417022 | 9423400 | Inversion |
| Chr2 | 9937207 | 9924967 | chr2 | 9379485 | 9391725 | Inversion |
| Chr2 | 9937798 | 9937358 | chr1 | 665804 | 666238 | Inversion |
| Chr2 | 9938940 | 9937855 | chr1 | 664583 | 665668 | Inversion |
| Chr2 | 9946132 | 9938085 | chr2 | 9371339 | 9379384 | Inversion |
| Chr2 | 9979950 | 9964782 | chr2 | 9353337 | 9368505 | Inversion |
| Chr2 | 10004941 | 10014018 | chr8 | 37592916 | 37601985 | Inter-translocation |
| Chr2 | 10077248 | 10077622 | chr3 | 39020795 | 39021174 | Inter-translocation |
| Chr2 | 10221955 | 10221726 | chr8 | 14339177 | 14339412 | Inversion |
| Chr2 | 10227024 | 10222007 | chr8 | 11518479 | 11523533 | Inversion |
| Chr2 | 10232999 | 10227297 | chr8 | 11512835 | 11518513 | Inversion |
| Chr2 | 10233198 | 10228774 | chr8 | 25189255 | 25193656 | Inversion |
| Chr2 | 10307280 | 10308653 | chr2 | 23874327 | 23875700 | Intra-translocation |
| Chr2 | 10307749 | 10312479 | chr1 | 2672095 | 2676830 | Inter-translocation |
| Chr2 | 10430192 | 10429986 | chr8 | 26193718 | 26193923 | Inversion |
| Chr2 | 10685514 | 10683847 | chr6 | 26666102 | 26667769 | Inversion |
| Chr2 | 10687415 | 10686556 | chr1 | 21451268 | 21452126 | Inversion |
| Chr2 | 10813960 | 10813793 | chr8 | 10203295 | 10203464 | Inversion |
| Chr2 | 10989504 | 10989731 | chr9 | 28852525 | 28852754 | Inter-translocation |
| Chr2 | 11005030 | 11000781 | chr5 | 16439982 | 16444233 | Inversion |
| Chr2 | 11343589 | 11344259 | chr8 | 29612713 | 29613359 | Inter-translocation |
| Chr2 | 11346386 | 11349202 | chr8 | 29618755 | 29621571 | Inter-translocation |
| Chr2 | 11540685 | 11541552 | chr3 | 49575653 | 49576559 | Inter-translocation |
| Chr2 | 11560536 | 11560185 | chr9 | 1836342 | 1836693 | Inversion |
| Chr2 | 12566499 | 12559714 | chr8 | 3190276 | 3197059 | Inversion |
| Chr2 | 12695455 | 12695572 | chr8 | 11972057 | 11972174 | Inter-translocation |
| Chr2 | 12724706 | 12725011 | chr8 | 40340518 | 40340823 | Inter-translocation |
| Chr2 | 13559277 | 13560313 | chr8 | 11098341 | 11099374 | Inter-translocation |
| Chr2 | 13662813 | 13663000 | chr8 | 4563814 | 4564002 | Inter-translocation |
| Chr2 | 13663560 | 13664504 | chr8 | 4564446 | 4565387 | Inter-translocation |
| Chr2 | 14044049 | 14043564 | chr9 | 32918374 | 32918881 | Inversion |
| Chr2 | 14044444 | 14044082 | chr8 | 3230732 | 3231090 | Inversion |
| Chr2 | 15120173 | 15132747 | chr2 | 13758171 | 13770730 | Intra-translocation |
| Chr2 | 15880706 | 15880370 | chr5 | 15903430 | 15903766 | Inversion |
| Chr2 | 16166275 | 16167339 | chr9 | 22756434 | 22757499 | Inter-translocation |
| Chr2 | 16449878 | 16449324 | chr9 | 56964855 | 56965400 | Inversion |
| Chr2 | 17480803 | 17481234 | chr6 | 2715358 | 2715773 | Inter-translocation |
| Chr2 | 17823709 | 17825447 | chr6 | 1073899 | 1075635 | Inter-translocation |
| Chr2 | 18064511 | 18063552 | chr8 | 34941921 | 34942886 | Inversion |
| Chr2 | 18562998 | 18557237 | chr3 | 24007190 | 24013022 | Inversion |
| Chr2 | 18633855 | 18624818 | chr6 | 23982255 | 23991293 | Inversion |
| Chr2 | 18656805 | 18656688 | chr9 | 2881728 | 2881845 | Inversion |
| Chr2 | 19867720 | 19883767 | chr2 | 9542053 | 9558101 | Intra-translocation |
| Chr2 | 19888966 | 19891958 | chr8 | 34948124 | 34951090 | Inter-translocation |
| Chr2 | 20410117 | 20409272 | chr5 | 15078376 | 15079227 | Inversion |
| Chr2 | 20432977 | 20440628 | chr9 | 24846107 | 24853759 | Inter-translocation |
| Chr2 | 23347859 | 23347324 | chr9 | 54606882 | 54607419 | Inversion |
| Chr2 | 23599820 | 23599738 | chr9 | 25362337 | 25362419 | Inversion |
| Chr2 | 23772979 | 23769051 | chr8 | 38311259 | 38315188 | Inversion |
| Chr2 | 23915628 | 23915828 | chr9 | 30591231 | 30591432 | Inter-translocation |
| Chr2 | 23919927 | 23921678 | chr2 | 22399927 | 22401677 | Intra-translocation |
| Chr2 | 24106792 | 24117470 | chr2 | 22525869 | 22536547 | Intra-translocation |
| Chr2 | 24193460 | 24176933 | chr2 | 20590810 | 20607337 | Inversion |
| Chr2 | 24199843 | 24193576 | chr2 | 20584557 | 20590824 | Inversion |
| Chr2 | 24200592 | 24193641 | chr8 | 5393233 | 5400198 | Inversion |
| Chr2 | 24234865 | 24234210 | chr4 | 35230554 | 35231209 | Inversion |
| Chr2 | 24302861 | 24287114 | chr2 | 22852684 | 22868403 | Inversion |
| Chr2 | 24522850 | 24522992 | chr8 | 10949497 | 10949640 | Inter-translocation |
| Chr2 | 24528410 | 24523360 | chr4 | 17424182 | 17429256 | Inversion |
| Chr2 | 24852281 | 24852009 | chr8 | 9609719 | 9609990 | Inversion |
| Chr2 | 24860205 | 24861851 | chr9 | 33636583 | 33638228 | Inter-translocation |
| Chr2 | 25218834 | 25218664 | chr8 | 37113330 | 37113499 | Inversion |
| Chr2 | 26478530 | 26475418 | chr2 | 14540618 | 14543730 | Inversion |
| Chr2 | 26794414 | 26792351 | chr5 | 17555779 | 17557825 | Inversion |
| Chr2 | 27053019 | 27051359 | chr2 | 3039014 | 3040674 | Inversion |
| Chr2 | 28846617 | 28845398 | chr8 | 14615733 | 14616909 | Inversion |
| Chr2 | 28850710 | 28849936 | chr8 | 25493105 | 25493897 | Inversion |
| Chr2 | 28855072 | 28852959 | chr3 | 50569192 | 50571306 | Inversion |
| Chr2 | 28971961 | 28979921 | chr6 | 22309394 | 22317347 | Inter-translocation |
| Chr2 | 29000400 | 29000193 | chr8 | 2857005 | 2857211 | Inversion |
| Chr2 | 29068078 | 29067632 | chr2 | 41630891 | 41631326 | Inversion |
| Chr2 | 29068459 | 29068178 | chr2 | 41630503 | 41630784 | Inversion |
| Chr2 | 29165958 | 29165156 | chr9 | 1732657 | 1733473 | Inversion |
| Chr2 | 29195243 | 29196581 | chr6 | 32671306 | 32672646 | Inter-translocation |
| Chr2 | 29196591 | 29197073 | chr6 | 32672493 | 32672964 | Inter-translocation |
| Chr2 | 29422975 | 29423693 | chr9 | 27319194 | 27319906 | Inter-translocation |
| Chr2 | 29539733 | 29539638 | chr8 | 38171998 | 38172094 | Inversion |
| Chr2 | 29589565 | 29589349 | chr7 | 30405139 | 30405355 | Inversion |
| Chr2 | 30033186 | 30033763 | chr6 | 31062225 | 31062802 | Inter-translocation |
| Chr2 | 30096783 | 30096589 | chr6 | 32767827 | 32768022 | Inversion |
| Chr2 | 30143153 | 30144464 | chr9 | 38553441 | 38554755 | Inter-translocation |
| Chr2 | 30160876 | 30161230 | chr2 | 41663257 | 41663611 | Intra-translocation |
| Chr2 | 30513067 | 30512711 | chr9 | 21763163 | 21763519 | Inversion |
| Chr2 | 30623882 | 30617936 | chr4 | 24878382 | 24884327 | Inversion |
| Chr2 | 32036562 | 32036280 | chr9 | 41967386 | 41967668 | Inversion |
| Chr2 | 33039035 | 33038123 | chr9 | 54511482 | 54512349 | Inversion |
| Chr2 | 33262437 | 33262652 | chr6 | 34337183 | 34337398 | Inter-translocation |
| Chr2 | 33308437 | 33308175 | chr3 | 26686585 | 26686847 | Inversion |
| Chr2 | 34075700 | 34076052 | chr6 | 29821961 | 29822313 | Inter-translocation |
| Chr2 | 34139472 | 34139116 | chr3 | 8820570 | 8820926 | Inversion |
| Chr2 | 35109161 | 35105702 | chr2 | 33296594 | 33300055 | Inversion |
| Chr2 | 35124338 | 35109303 | chr2 | 33281424 | 33296460 | Inversion |
| Chr2 | 35129145 | 35123350 | chr2 | 33300056 | 33305872 | Inversion |
| Chr2 | 35620168 | 35620438 | chr6 | 2606273 | 2606542 | Inter-translocation |
| Chr2 | 36465326 | 36463435 | chr9 | 21603434 | 21605310 | Inversion |
| Chr2 | 37730856 | 37731381 | chr2 | 42662296 | 42662827 | Intra-translocation |
| Chr2 | 38291923 | 38292231 | chr9 | 2881059 | 2881366 | Inter-translocation |
| Chr2 | 38292530 | 38292728 | chr5 | 24480138 | 24480335 | Inter-translocation |
| Chr2 | 38313327 | 38313023 | chr8 | 27768002 | 27768306 | Inversion |
| Chr2 | 38841644 | 38841299 | chr6 | 2699017 | 2699362 | Inversion |
| Chr2 | 39103344 | 39102994 | chr9 | 17616355 | 17616705 | Inversion |
| Chr2 | 39244478 | 39244107 | chr9 | 2968716 | 2969091 | Inversion |
| Chr2 | 39244532 | 39244108 | chr9 | 37542763 | 37543184 | Inversion |
| Chr2 | 39254774 | 39254495 | chr9 | 10340700 | 10340979 | Inversion |
| Chr2 | 40034516 | 40032645 | chr6 | 22316757 | 22318625 | Inversion |
| Chr2 | 40404077 | 40404348 | chr5 | 9047283 | 9047554 | Inter-translocation |
| Chr2 | 40590726 | 40596379 | chr4 | 3454150 | 3459790 | Inter-translocation |
| Chr2 | 40955867 | 40956342 | chr2 | 37188935 | 37189410 | Intra-translocation |
| Chr2 | 41051134 | 41050939 | chr8 | 8217319 | 8217513 | Inversion |
| Chr2 | 41990895 | 41990997 | chr1 | 829083 | 829185 | Inter-translocation |
| Chr2 | 42322759 | 42322502 | chr8 | 36872963 | 36873220 | Inversion |
| Chr2 | 42405371 | 42400564 | chr8 | 8649539 | 8654348 | Inversion |
| Chr2 | 42686765 | 42686428 | chr4 | 7102299 | 7102636 | Inversion |
| Chr2 | 42716912 | 42714087 | chr8 | 40420823 | 40423654 | Inversion |
| Chr2 | 42791085 | 42789499 | chr8 | 7792046 | 7793638 | Inversion |
| Chr2 | 43013608 | 43012366 | chr8 | 13677882 | 13679154 | Inversion |
| Chr2 | 43044390 | 43044054 | chr9 | 32365676 | 32366013 | Inversion |
| Chr2 | 43045927 | 43045647 | chr9 | 16722549 | 16722829 | Inversion |
| Chr2 | 43070569 | 43070915 | chr9 | 34610821 | 34611168 | Inter-translocation |
| Chr2 | 43099770 | 43091768 | chr8 | 15064886 | 15072891 | Inversion |
| Chr2 | 43132365 | 43132623 | chr5 | 15564929 | 15565187 | Inter-translocation |
| Chr2 | 43133989 | 43133708 | chr6 | 30986220 | 30986501 | Inversion |
| Chr2 | 43164932 | 43164732 | chr5 | 9390677 | 9390877 | Inversion |
| Chr2 | 43376280 | 43380356 | chr2 | 42637495 | 42641577 | Intra-translocation |
| Chr2 | 43381119 | 43381691 | chr8 | 17856642 | 17857214 | Inter-translocation |
| Chr2 | 43388982 | 43394651 | chr2 | 42645986 | 42651770 | Intra-translocation |
| Chr2 | 43392906 | 43386861 | chr2 | 41636029 | 41642056 | Inversion |
| Chr2 | 43397443 | 43393066 | chr2 | 41631551 | 41635987 | Inversion |
| Chr2 | 43530493 | 43529356 | chr4 | 34192580 | 34193720 | Inversion |
| Chr2 | 43732014 | 43731655 | chr7 | 23222128 | 23222486 | Inversion |
| Chr2 | 43747980 | 43747516 | chr8 | 22894768 | 22895234 | Inversion |
| Chr2 | 43819509 | 43819774 | chr6 | 35859544 | 35859809 | Inter-translocation |
| Chr2 | 43871145 | 43870563 | chr2 | 41905639 | 41906231 | Inversion |
| Chr2 | 43939889 | 43939645 | chr2 | 42442338 | 42442582 | Inversion |
| Chr2 | 43960383 | 43960160 | chr3 | 49488830 | 49489051 | Inversion |
| Chr2 | 44030304 | 44030765 | chr7 | 285338 | 285799 | Inter-translocation |
| Chr2 | 44081788 | 44082139 | chr2 | 47720362 | 47720713 | Intra-translocation |
| Chr2 | 44115520 | 44115138 | chr8 | 9607600 | 9607982 | Inversion |
| Chr2 | 44157002 | 44156548 | chr2 | 42370476 | 42370929 | Inversion |
| Chr2 | 44157416 | 44156740 | chr2 | 42373224 | 42373900 | Inversion |
| Chr2 | 44162868 | 44160533 | chr2 | 41633674 | 41636011 | Inversion |
| Chr2 | 45150744 | 45151087 | chr9 | 34767272 | 34767616 | Inter-translocation |
| Chr2 | 45196331 | 45192403 | chr5 | 8542057 | 8546010 | Inversion |
| Chr2 | 45354323 | 45354578 | chr9 | 49711581 | 49711836 | Inter-translocation |
| Chr2 | 45653758 | 45653462 | chr4 | 34273377 | 34273674 | Inversion |
| Chr2 | 46597293 | 46596935 | chr2 | 44731088 | 44731446 | Inversion |
| Chr2 | 46695061 | 46694714 | chr2 | 48943970 | 48944317 | Inversion |
| Chr2 | 46897722 | 46898075 | chr2 | 45750247 | 45750600 | Intra-translocation |
| Chr2 | 47101251 | 47101329 | chr2 | 43869137 | 43869215 | Intra-translocation |
| Chr2 | 47469779 | 47470137 | chr2 | 27592006 | 27592364 | Intra-translocation |
| Chr2 | 47721104 | 47720756 | chr3 | 2814991 | 2815339 | Inversion |
| Chr2 | 47922653 | 47922484 | chr9 | 17151284 | 17151453 | Inversion |
| Chr2 | 48494255 | 48493902 | chr9 | 58316940 | 58317293 | Inversion |
| Chr3 | 942862 | 944013 | chr7 | 4473317 | 4474468 | Inter-translocation |
| Chr3 | 991790 | 992047 | chr3 | 24325521 | 24325777 | Intra-translocation |
| Chr3 | 2278829 | 2279183 | chr3 | 7286554 | 7286908 | Intra-translocation |
| Chr3 | 2613383 | 2613028 | chr9 | 10024460 | 10024815 | Inversion |
| Chr3 | 2757445 | 2757090 | chr3 | 7292075 | 7292430 | Inversion |
| Chr3 | 2771212 | 2770268 | chr9 | 36532222 | 36533166 | Inversion |
| Chr3 | 2883654 | 2883934 | chr6 | 32172503 | 32172783 | Inter-translocation |
| Chr3 | 3020211 | 3019957 | chr4 | 12083950 | 12084205 | Inversion |
| Chr3 | 3483619 | 3483577 | chr6 | 3725116 | 3725159 | Inversion |
| Chr3 | 4043798 | 4043450 | chr6 | 28614776 | 28615124 | Inversion |
| Chr3 | 4046070 | 4053072 | chr8 | 40252011 | 40259013 | Inter-translocation |
| Chr3 | 4336271 | 4334319 | chr3 | 47107829 | 47109797 | Inversion |
| Chr3 | 4964490 | 4964302 | chr9 | 33408731 | 33408919 | Inversion |
| Chr3 | 5193295 | 5193652 | chr9 | 52826832 | 52827189 | Inter-translocation |
| Chr3 | 5715523 | 5716793 | chr8 | 11004669 | 11005930 | Inter-translocation |
| Chr3 | 5741563 | 5738373 | chr8 | 22106872 | 22110110 | Inversion |
| Chr3 | 5748435 | 5747156 | chr7 | 31784119 | 31785421 | Inversion |
| Chr3 | 5882621 | 5882204 | chr8 | 25554577 | 25554996 | Inversion |
| Chr3 | 5944352 | 5944695 | chr6 | 28612668 | 28613011 | Inter-translocation |
| Chr3 | 5986256 | 5985654 | chr9 | 28911250 | 28911854 | Inversion |
| Chr3 | 6011930 | 6015628 | chr3 | 6068708 | 6072430 | Intra-translocation |
| Chr3 | 6015623 | 6017910 | chr3 | 6043558 | 6045846 | Intra-translocation |
| Chr3 | 6137205 | 6136835 | chr7 | 33405131 | 33405507 | Inversion |
| Chr3 | 6328129 | 6327871 | chr9 | 20511233 | 20511492 | Inversion |
| Chr3 | 6567120 | 6566941 | chr8 | 26106024 | 26106203 | Inversion |
| Chr3 | 6569345 | 6569032 | chr8 | 25113082 | 25113390 | Inversion |
| Chr3 | 6795454 | 6795307 | chr4 | 37234769 | 37234917 | Inversion |
| Chr3 | 6803192 | 6803431 | chr4 | 918372 | 918611 | Inter-translocation |
| Chr3 | 6821640 | 6821840 | chr8 | 13065877 | 13066075 | Inter-translocation |
| Chr3 | 6843496 | 6843268 | chr9 | 18704015 | 18704243 | Inversion |
| Chr3 | 6867189 | 6866841 | chr2 | 44490709 | 44491057 | Inversion |
| Chr3 | 6911583 | 6911790 | chr8 | 5995652 | 5995859 | Inter-translocation |
| Chr3 | 7068239 | 7067895 | chr8 | 29842761 | 29843105 | Inversion |
| Chr3 | 7097695 | 7096760 | chr3 | 13409783 | 13410717 | Inversion |
| Chr3 | 7097967 | 7097275 | chr3 | 13421962 | 13422632 | Inversion |
| Chr3 | 7098317 | 7098730 | chr8 | 39620873 | 39621287 | Inter-translocation |
| Chr3 | 7251045 | 7250760 | chr9 | 21702560 | 21702845 | Inversion |
| Chr3 | 7279838 | 7280118 | chr7 | 27595769 | 27596049 | Inter-translocation |
| Chr3 | 7480976 | 7480712 | chr8 | 29165062 | 29165326 | Inversion |
| Chr3 | 7518917 | 7519052 | chr8 | 4564161 | 4564295 | Inter-translocation |
| Chr3 | 8076280 | 8076553 | chr9 | 41264528 | 41264801 | Inter-translocation |
| Chr3 | 8242555 | 8249124 | chr8 | 37947017 | 37953616 | Inter-translocation |
| Chr3 | 8369576 | 8369696 | chr1 | 24910385 | 24910505 | Inter-translocation |
| Chr3 | 8444281 | 8443377 | chr8 | 2939382 | 2940296 | Inversion |
| Chr3 | 8487644 | 8487869 | chr9 | 28852524 | 28852749 | Inter-translocation |
| Chr3 | 8954218 | 8953992 | chr8 | 14615341 | 14615563 | Inversion |
| Chr3 | 8955642 | 8954574 | chr3 | 13325254 | 13326361 | Inversion |
| Chr3 | 9409087 | 9409434 | chr9 | 2753424 | 2753771 | Inter-translocation |
| Chr3 | 9409398 | 9409086 | chr9 | 444400 | 444712 | Inversion |
| Chr3 | 10063937 | 10060872 | chr9 | 21621806 | 21624856 | Inversion |
| Chr3 | 10534795 | 10534512 | chr5 | 15520756 | 15521039 | Inversion |
| Chr3 | 10754127 | 10737608 | chr8 | 31359041 | 31375553 | Inversion |
| Chr3 | 10928093 | 10927769 | chr8 | 9956349 | 9956673 | Inversion |
| Chr3 | 11470447 | 11463675 | chr3 | 11560915 | 11567690 | Inversion |
| Chr3 | 11473570 | 11470433 | chr3 | 11554952 | 11558089 | Inversion |
| Chr3 | 11483933 | 11484291 | chr8 | 23026940 | 23027299 | Inter-translocation |
| Chr3 | 11483935 | 11473922 | chr3 | 11544936 | 11554950 | Inversion |
| Chr3 | 11490953 | 11484290 | chr3 | 11538276 | 11544940 | Inversion |
| Chr3 | 11494876 | 11491710 | chr3 | 11534958 | 11538123 | Inversion |
| Chr3 | 11514034 | 11494873 | chr3 | 11515432 | 11534583 | Inversion |
| Chr3 | 11518242 | 11514351 | chr3 | 11511233 | 11515120 | Inversion |
| Chr3 | 11532006 | 11518308 | chr3 | 11497434 | 11511126 | Inversion |
| Chr3 | 11541230 | 11532279 | chr3 | 11488487 | 11497438 | Inversion |
| Chr3 | 11815504 | 11708389 | chr5 | 29274990 | 29382101 | Inversion |
| Chr3 | 11845522 | 11816016 | chr5 | 29245393 | 29274889 | Inversion |
| Chr3 | 11945879 | 11845683 | chr5 | 29145051 | 29245239 | Inversion |
| Chr3 | 11953375 | 11946034 | chr5 | 29137011 | 29144353 | Inversion |
| Chr3 | 12130962 | 11953488 | chr5 | 28959231 | 29136697 | Inversion |
| Chr3 | 12145847 | 12132395 | chr5 | 28945779 | 28959231 | Inversion |
| Chr3 | 12153773 | 12145895 | chr5 | 28937612 | 28945490 | Inversion |
| Chr3 | 12159793 | 12153766 | chr5 | 28929730 | 28935757 | Inversion |
| Chr3 | 12173514 | 12159998 | chr5 | 28916000 | 28929518 | Inversion |
| Chr3 | 13344867 | 13344795 | chr8 | 10770368 | 10770440 | Inversion |
| Chr3 | 13395051 | 13394941 | chr7 | 22756901 | 22757011 | Inversion |
| Chr3 | 13598068 | 13595030 | chr8 | 13702601 | 13705609 | Inversion |
| Chr3 | 13669534 | 13669679 | chr6 | 30539715 | 30539860 | Inter-translocation |
| Chr3 | 13792294 | 13792611 | chr3 | 13406213 | 13406524 | Intra-translocation |
| Chr3 | 13794643 | 13795108 | chr3 | 13408121 | 13408575 | Intra-translocation |
| Chr3 | 14181826 | 14181605 | chr3 | 47034960 | 47035184 | Inversion |
| Chr3 | 14196335 | 14196622 | chr6 | 33196015 | 33196302 | Inter-translocation |
| Chr3 | 14336501 | 14337222 | chr5 | 15205831 | 15206552 | Inter-translocation |
| Chr3 | 14930971 | 14930659 | chr8 | 10939317 | 10939629 | Inversion |
| Chr3 | 14954232 | 14952238 | chr3 | 14659875 | 14661863 | Inversion |
| Chr3 | 14961602 | 14954228 | chr3 | 14649342 | 14656701 | Inversion |
| Chr3 | 14977656 | 14961624 | chr3 | 14631646 | 14647687 | Inversion |
| Chr3 | 14977930 | 14977657 | chr1 | 26289724 | 26289997 | Inversion |
| Chr3 | 14978565 | 14977978 | chr3 | 14630988 | 14631576 | Inversion |
| Chr3 | 14979751 | 14978589 | chr3 | 14629854 | 14631015 | Inversion |
| Chr3 | 14985774 | 14980397 | chr3 | 14624459 | 14629854 | Inversion |
| Chr3 | 14987207 | 14986058 | chr3 | 14623301 | 14624456 | Inversion |
| Chr3 | 14994344 | 14987324 | chr3 | 14615900 | 14622961 | Inversion |
| Chr3 | 15067641 | 15067323 | chr9 | 25101546 | 25101864 | Inversion |
| Chr3 | 15080295 | 15079915 | chr8 | 25933216 | 25933596 | Inversion |
| Chr3 | 15120101 | 15120002 | chr9 | 18663947 | 18664046 | Inversion |
| Chr3 | 15492229 | 15487671 | chr8 | 19435960 | 19440523 | Inversion |
| Chr3 | 15495402 | 15490402 | chr8 | 22557469 | 22562469 | Inversion |
| Chr3 | 17012081 | 17007205 | chr5 | 8968038 | 8972949 | Inversion |
| Chr3 | 17139068 | 17147182 | chr8 | 20713188 | 20721292 | Inter-translocation |
| Chr3 | 17313053 | 17322897 | chr3 | 16726357 | 16736195 | Intra-translocation |
| Chr3 | 17322947 | 17323958 | chr3 | 16736296 | 16737307 | Intra-translocation |
| Chr3 | 17345176 | 17360752 | chr3 | 16470060 | 16485633 | Intra-translocation |
| Chr3 | 17360663 | 17363707 | chr3 | 16485808 | 16488851 | Intra-translocation |
| Chr3 | 17363742 | 17363958 | chr9 | 17853330 | 17853546 | Inter-translocation |
| Chr3 | 17431903 | 17435054 | chr8 | 22754553 | 22757719 | Inter-translocation |
| Chr3 | 17482726 | 17481350 | chr9 | 31972312 | 31973673 | Inversion |
| Chr3 | 17488411 | 17483255 | chr8 | 21367451 | 21372612 | Inversion |
| Chr3 | 17493607 | 17497353 | chr9 | 29475079 | 29478847 | Inter-translocation |
| Chr3 | 17648538 | 17648321 | chr3 | 5695765 | 5695982 | Inversion |
| Chr3 | 18616129 | 18616553 | chr9 | 17950507 | 17950917 | Inter-translocation |
| Chr3 | 19618815 | 19619121 | chr9 | 32593654 | 32593957 | Inter-translocation |
| Chr3 | 19719680 | 19719830 | chr9 | 32683458 | 32683607 | Inter-translocation |
| Chr3 | 19882117 | 19878175 | chr9 | 31000080 | 31004020 | Inversion |
| Chr3 | 20593412 | 20592589 | chr8 | 10948649 | 10949431 | Inversion |
| Chr3 | 20595625 | 20593698 | chr1 | 2669101 | 2671018 | Inversion |
| Chr3 | 22175498 | 22174353 | chr4 | 7171296 | 7172437 | Inversion |
| Chr3 | 22318957 | 22314608 | chr7 | 8540171 | 8544494 | Inversion |
| Chr3 | 22328790 | 22318888 | chr7 | 8548765 | 8558663 | Inversion |
| Chr3 | 22332961 | 22328948 | chr7 | 8544595 | 8548607 | Inversion |
| Chr3 | 22938770 | 22938958 | chr6 | 32231949 | 32232136 | Inter-translocation |
| Chr3 | 23103598 | 23103319 | chr4 | 7433431 | 7433711 | Inversion |
| Chr3 | 23295152 | 23286641 | chr9 | 39291987 | 39300500 | Inversion |
| Chr3 | 23399417 | 23398871 | chr8 | 2471267 | 2471809 | Inversion |
| Chr3 | 23411328 | 23410372 | chr9 | 18334072 | 18335019 | Inversion |
| Chr3 | 23481215 | 23483094 | chr4 | 40384704 | 40386569 | Inter-translocation |
| Chr3 | 23846848 | 23846527 | chr2 | 42054076 | 42054387 | Inversion |
| Chr3 | 23865978 | 23865696 | chr8 | 23199996 | 23200279 | Inversion |
| Chr3 | 23892293 | 23892546 | chr1 | 27136905 | 27137158 | Inter-translocation |
| Chr3 | 23916145 | 23921609 | chr3 | 7531328 | 7536790 | Intra-translocation |
| Chr3 | 23929126 | 23929320 | chr8 | 10215278 | 10215472 | Inter-translocation |
| Chr3 | 24004510 | 24000763 | chr1 | 26107502 | 26111252 | Inversion |
| Chr3 | 24065703 | 24064641 | chr8 | 6880034 | 6881124 | Inversion |
| Chr3 | 24069160 | 24067901 | chr8 | 6878474 | 6879765 | Inversion |
| Chr3 | 24116814 | 24130053 | chr8 | 21917592 | 21930837 | Inter-translocation |
| Chr3 | 24185995 | 24185655 | chr6 | 1529400 | 1529738 | Inversion |
| Chr3 | 24240759 | 24241023 | chr1 | 25385304 | 25385568 | Inter-translocation |
| Chr3 | 24264689 | 24256024 | chr9 | 12122999 | 12131631 | Inversion |
| Chr3 | 24587645 | 24587989 | chr9 | 22185247 | 22185592 | Inter-translocation |
| Chr3 | 24724331 | 24717456 | chr8 | 13394300 | 13401175 | Inversion |
| Chr3 | 24728348 | 24724327 | chr8 | 13389571 | 13393590 | Inversion |
| Chr3 | 24827003 | 24827970 | chr8 | 20796984 | 20797951 | Inter-translocation |
| Chr3 | 24886719 | 24887214 | chr7 | 27020405 | 27020898 | Inter-translocation |
| Chr3 | 24888586 | 24888878 | chr7 | 27023454 | 27023746 | Inter-translocation |
| Chr3 | 25122431 | 25120184 | chr3 | 45219675 | 45221924 | Inversion |
| Chr3 | 25220176 | 25212452 | chr4 | 2387345 | 2395058 | Inversion |
| Chr3 | 25243088 | 25247138 | chr8 | 10543932 | 10547983 | Inter-translocation |
| Chr3 | 25285995 | 25287960 | chr2 | 46059054 | 46061019 | Inter-translocation |
| Chr3 | 25287936 | 25283748 | chr5 | 23190841 | 23195024 | Inversion |
| Chr3 | 25305248 | 25297873 | chr1 | 24544560 | 24551926 | Inversion |
| Chr3 | 25328830 | 25324973 | chr9 | 27703714 | 27707600 | Inversion |
| Chr3 | 25337728 | 25337388 | chr3 | 47329781 | 47330121 | Inversion |
| Chr3 | 25707095 | 25707630 | chr6 | 34260130 | 34260670 | Inter-translocation |
| Chr3 | 25765074 | 25764666 | chr8 | 12422038 | 12422446 | Inversion |
| Chr3 | 25775139 | 25772090 | chr9 | 30367951 | 30370999 | Inversion |
| Chr3 | 25870457 | 25874956 | chr1 | 2666512 | 2671011 | Inter-translocation |
| Chr3 | 25876426 | 25876285 | chr9 | 28857356 | 28857497 | Inversion |
| Chr3 | 25877839 | 25881684 | chr7 | 14775081 | 14778910 | Inter-translocation |
| Chr3 | 26182509 | 26185631 | chr4 | 7318003 | 7321123 | Inter-translocation |
| Chr3 | 26734185 | 26730629 | chr3 | 40213986 | 40217545 | Inversion |
| Chr3 | 26816386 | 26816039 | chr1 | 24048829 | 24049176 | Inversion |
| Chr3 | 26818331 | 26817981 | chr3 | 12961062 | 12961412 | Inversion |
| Chr3 | 28086782 | 28086633 | chr5 | 9073034 | 9073184 | Inversion |
| Chr3 | 28412766 | 28413330 | chr8 | 25328943 | 25329499 | Inter-translocation |
| Chr3 | 28709683 | 28710001 | chr8 | 10609336 | 10609657 | Inter-translocation |
| Chr3 | 28711803 | 28710792 | chr8 | 7920317 | 7921327 | Inversion |
| Chr3 | 28774303 | 28771752 | chr4 | 21331523 | 21334083 | Inversion |
| Chr3 | 29205329 | 29204593 | chr6 | 22287784 | 22288549 | Inversion |
| Chr3 | 29867921 | 29869654 | chr8 | 29628201 | 29629937 | Inter-translocation |
| Chr3 | 30173575 | 30168617 | chr3 | 13340491 | 13345452 | Inversion |
| Chr3 | 31793152 | 31791935 | chr8 | 19532489 | 19533706 | Inversion |
| Chr3 | 32163139 | 32162560 | chr4 | 21331520 | 21332099 | Inversion |
| Chr3 | 32194409 | 32184222 | chr7 | 7707718 | 7717917 | Inversion |
| Chr3 | 32521614 | 32522533 | chr3 | 33761227 | 33762145 | Intra-translocation |
| Chr3 | 33818329 | 33821278 | chr9 | 30994581 | 30997538 | Inter-translocation |
| Chr3 | 34566722 | 34565800 | chr7 | 22074292 | 22075214 | Inversion |
| Chr3 | 34801041 | 34801575 | chr8 | 12266567 | 12267114 | Inter-translocation |
| Chr3 | 34801551 | 34801765 | chr8 | 10425357 | 10425571 | Inter-translocation |
| Chr3 | 34835870 | 34835325 | chr1 | 24380470 | 24381006 | Inversion |
| Chr3 | 35070455 | 35021459 | chr3 | 38271018 | 38320014 | Inversion |
| Chr3 | 35070957 | 35070649 | chr6 | 30065269 | 30065582 | Inversion |
| Chr3 | 35073255 | 35070839 | chr3 | 38268367 | 38270783 | Inversion |
| Chr3 | 35076559 | 35073366 | chr3 | 38265084 | 38268266 | Inversion |
| Chr3 | 35148591 | 35076372 | chr3 | 38193170 | 38265396 | Inversion |
| Chr3 | 35307564 | 35149283 | chr3 | 38033900 | 38192172 | Inversion |
| Chr3 | 35458481 | 35307563 | chr3 | 37883744 | 38034658 | Inversion |
| Chr3 | 35611636 | 35458988 | chr3 | 37730807 | 37883451 | Inversion |
| Chr3 | 35657244 | 35618390 | chr3 | 37691956 | 37730811 | Inversion |
| Chr3 | 35685906 | 35657358 | chr3 | 37663060 | 37691610 | Inversion |
| Chr3 | 35752610 | 35686187 | chr3 | 37596314 | 37662733 | Inversion |
| Chr3 | 35828282 | 35752776 | chr3 | 37520530 | 37596052 | Inversion |
| Chr3 | 35985004 | 35808288 | chr3 | 37363809 | 37540535 | Inversion |
| Chr3 | 35987332 | 35984959 | chr3 | 37360747 | 37363119 | Inversion |
| Chr3 | 35993063 | 35988285 | chr3 | 37356770 | 37361549 | Inversion |
| Chr3 | 36007874 | 35993121 | chr3 | 37341915 | 37356669 | Inversion |
| Chr3 | 36112193 | 36007867 | chr3 | 37237181 | 37341509 | Inversion |
| Chr3 | 36142948 | 36112863 | chr3 | 37206995 | 37237080 | Inversion |
| Chr3 | 36190131 | 36143407 | chr3 | 37159718 | 37206426 | Inversion |
| Chr3 | 36247768 | 36170140 | chr3 | 37102100 | 37179691 | Inversion |
| Chr3 | 36413451 | 36227741 | chr3 | 36936424 | 37122117 | Inversion |
| Chr3 | 36444271 | 36430616 | chr3 | 36926970 | 36940623 | Inversion |
| Chr3 | 36446319 | 36444521 | chr3 | 36920379 | 36922177 | Inversion |
| Chr3 | 36454205 | 36446343 | chr3 | 36912405 | 36920278 | Inversion |
| Chr3 | 36600420 | 36454444 | chr3 | 36766201 | 36912165 | Inversion |
| Chr3 | 36619266 | 36600457 | chr3 | 36747252 | 36766060 | Inversion |
| Chr3 | 36628132 | 36619299 | chr3 | 36738315 | 36747147 | Inversion |
| Chr3 | 36736099 | 36628169 | chr3 | 36630248 | 36738178 | Inversion |
| Chr3 | 36776957 | 36736197 | chr3 | 36589341 | 36630099 | Inversion |
| Chr3 | 36783046 | 36776957 | chr3 | 36583155 | 36589243 | Inversion |
| Chr3 | 36821644 | 36783038 | chr3 | 36544519 | 36583125 | Inversion |
| Chr3 | 36830256 | 36821773 | chr3 | 36536130 | 36544616 | Inversion |
| Chr3 | 36832152 | 36830335 | chr1 | 6761103 | 6762920 | Inversion |
| Chr3 | 36875736 | 36831114 | chr3 | 36491084 | 36535691 | Inversion |
| Chr3 | 37093984 | 36876072 | chr3 | 36273180 | 36491088 | Inversion |
| Chr3 | 37102368 | 37094141 | chr3 | 36265241 | 36273466 | Inversion |
| Chr3 | 37126167 | 37104255 | chr3 | 36242317 | 36264225 | Inversion |
| Chr3 | 37161545 | 37127498 | chr3 | 36206868 | 36240921 | Inversion |
| Chr3 | 37262986 | 37161720 | chr3 | 36104490 | 36205764 | Inversion |
| Chr3 | 37342734 | 37265399 | chr3 | 36025287 | 36102617 | Inversion |
| Chr3 | 37367649 | 37343620 | chr3 | 36001144 | 36025170 | Inversion |
| Chr3 | 37456371 | 37367866 | chr3 | 35912398 | 36000889 | Inversion |
| Chr3 | 37477998 | 37456412 | chr3 | 35890902 | 35912444 | Inversion |
| Chr3 | 37487344 | 37491753 | chr8 | 26023785 | 26028176 | Inter-translocation |
| Chr3 | 37491379 | 37478087 | chr3 | 35877647 | 35890938 | Inversion |
| Chr3 | 37497532 | 37491917 | chr3 | 35871658 | 35877266 | Inversion |
| Chr3 | 37669051 | 37497686 | chr3 | 35700282 | 35871658 | Inversion |
| Chr3 | 37786542 | 37664297 | chr3 | 35573289 | 35695532 | Inversion |
| Chr3 | 37925445 | 37786583 | chr3 | 35434433 | 35573331 | Inversion |
| Chr3 | 37935539 | 37926240 | chr3 | 35423372 | 35432669 | Inversion |
| Chr3 | 38021226 | 37935873 | chr3 | 35337909 | 35423271 | Inversion |
| Chr3 | 38102209 | 38021357 | chr3 | 35257152 | 35337992 | Inversion |
| Chr3 | 38104914 | 38104849 | chr4 | 7207702 | 7207767 | Inversion |
| Chr3 | 38145650 | 38105014 | chr3 | 35215648 | 35256272 | Inversion |
| Chr3 | 38146108 | 38145856 | chr8 | 29157235 | 29157487 | Inversion |
| Chr3 | 38146453 | 38145915 | chr3 | 35214845 | 35215383 | Inversion |
| Chr3 | 38175643 | 38146545 | chr3 | 35185612 | 35214709 | Inversion |
| Chr3 | 38178594 | 38176721 | chr3 | 35182952 | 35184827 | Inversion |
| Chr3 | 38208555 | 38178616 | chr3 | 35153067 | 35183008 | Inversion |
| Chr3 | 38225237 | 38212110 | chr3 | 35137324 | 35150449 | Inversion |
| Chr3 | 38267519 | 38225477 | chr3 | 35095171 | 35137218 | Inversion |
| Chr3 | 38311929 | 38247531 | chr3 | 35050761 | 35115165 | Inversion |
| Chr3 | 38340812 | 38312571 | chr3 | 35022540 | 35050762 | Inversion |
| Chr3 | 38481361 | 38320831 | chr3 | 34881992 | 35042502 | Inversion |
| Chr3 | 38487175 | 38481619 | chr3 | 34876436 | 34881992 | Inversion |
| Chr3 | 38617105 | 38487545 | chr3 | 34746755 | 34876276 | Inversion |
| Chr3 | 38619758 | 38622237 | chr9 | 23196898 | 23199376 | Inter-translocation |
| Chr3 | 38668550 | 38622331 | chr3 | 34695697 | 34741916 | Inversion |
| Chr3 | 38786873 | 38668623 | chr3 | 34577533 | 34695779 | Inversion |
| Chr3 | 38801467 | 38787148 | chr3 | 34563098 | 34577432 | Inversion |
| Chr3 | 38820800 | 38801605 | chr3 | 34543794 | 34562980 | Inversion |
| Chr3 | 38824498 | 38821170 | chr3 | 34540469 | 34543795 | Inversion |
| Chr3 | 38839823 | 38824511 | chr3 | 34525233 | 34540547 | Inversion |
| Chr3 | 39030252 | 38839842 | chr3 | 34335184 | 34525585 | Inversion |
| Chr3 | 39035442 | 39030285 | chr3 | 34328154 | 34333311 | Inversion |
| Chr3 | 39130464 | 39035436 | chr3 | 34233037 | 34328050 | Inversion |
| Chr3 | 39207493 | 39130517 | chr3 | 34155954 | 34232936 | Inversion |
| Chr3 | 39208754 | 39207735 | chr3 | 34154610 | 34155620 | Inversion |
| Chr3 | 39224501 | 39211095 | chr3 | 34140782 | 34154187 | Inversion |
| Chr3 | 39229308 | 39224734 | chr3 | 34133850 | 34138419 | Inversion |
| Chr3 | 39289377 | 39288344 | chr8 | 2697996 | 2699051 | Inversion |
| Chr3 | 39644806 | 39645563 | chr3 | 22972569 | 22973340 | Intra-translocation |
| Chr3 | 39947722 | 39942833 | chr8 | 10943788 | 10948688 | Inversion |
| Chr3 | 40089207 | 40088522 | chr8 | 19021761 | 19022446 | Inversion |
| Chr3 | 40141857 | 40130244 | chr8 | 4977159 | 4988748 | Inversion |
| Chr3 | 40329786 | 40329099 | chr8 | 8074570 | 8075257 | Inversion |
| Chr3 | 40748175 | 40748046 | chr1 | 12625338 | 12625464 | Inversion |
| Chr3 | 41879660 | 41879430 | chr7 | 26586065 | 26586293 | Inversion |
| Chr3 | 41938648 | 41938518 | chr8 | 26006712 | 26006842 | Inversion |
| Chr3 | 42215987 | 42216332 | chr6 | 34627515 | 34627861 | Inter-translocation |
| Chr3 | 42814090 | 42816584 | chr3 | 41902725 | 41905224 | Intra-translocation |
| Chr3 | 42890957 | 42892344 | chr3 | 42019402 | 42020789 | Intra-translocation |
| Chr3 | 43193319 | 43192257 | chr2 | 3337579 | 3338647 | Inversion |
| Chr3 | 43199169 | 43200982 | chr5 | 15806762 | 15808573 | Inter-translocation |
| Chr3 | 43217841 | 43217627 | chr8 | 7563387 | 7563603 | Inversion |
| Chr3 | 43223680 | 43218762 | chr9 | 29371981 | 29376912 | Inversion |
| Chr3 | 43233900 | 43226908 | chr3 | 13337381 | 13344358 | Inversion |
| Chr3 | 44112849 | 44109701 | chr9 | 31080528 | 31083677 | Inversion |
| Chr3 | 44124176 | 44128886 | chr8 | 26111798 | 26116509 | Inter-translocation |
| Chr3 | 44130289 | 44128887 | chr9 | 30542488 | 30543891 | Inversion |
| Chr3 | 44640835 | 44640909 | chr1 | 40972532 | 40972606 | Inter-translocation |
| Chr3 | 45445904 | 45445751 | chr4 | 7414107 | 7414260 | Inversion |
| Chr3 | 45540129 | 45540274 | chr3 | 46265846 | 46265991 | Intra-translocation |
| Chr3 | 45615419 | 45615534 | chr9 | 40961834 | 40961949 | Inter-translocation |
| Chr3 | 45623687 | 45625840 | chr9 | 31477175 | 31479331 | Inter-translocation |
| Chr3 | 45682537 | 45679020 | chr8 | 9100239 | 9103756 | Inversion |
| Chr3 | 45766082 | 45766474 | chr6 | 34514123 | 34514515 | Inter-translocation |
| Chr3 | 45847995 | 45847695 | chr9 | 35284410 | 35284708 | Inversion |
| Chr3 | 45851743 | 45856947 | chr3 | 44831527 | 44836719 | Intra-translocation |
| Chr3 | 45857721 | 45861113 | chr3 | 44818582 | 44821961 | Intra-translocation |
| Chr3 | 47054853 | 47054969 | chr2 | 48840024 | 48840140 | Inter-translocation |
| Chr3 | 47094644 | 47095827 | chr2 | 3050219 | 3051428 | Inter-translocation |
| Chr3 | 47098009 | 47097819 | chr8 | 5731986 | 5732176 | Inversion |
| Chr3 | 47112307 | 47111921 | chr7 | 30969702 | 30970088 | Inversion |
| Chr3 | 47128251 | 47128526 | chr8 | 12079929 | 12080204 | Inter-translocation |
| Chr3 | 47139717 | 47139072 | chr1 | 14693643 | 14694299 | Inversion |
| Chr3 | 47155969 | 47158167 | chr1 | 23950245 | 23952426 | Inter-translocation |
| Chr3 | 47200205 | 47199516 | chr1 | 14692049 | 14692740 | Inversion |
| Chr3 | 47253253 | 47253019 | chr9 | 30870846 | 30871080 | Inversion |
| Chr3 | 47332860 | 47332069 | chr8 | 11674399 | 11675190 | Inversion |
| Chr3 | 47350345 | 47351775 | chr8 | 9980363 | 9981792 | Inter-translocation |
| Chr3 | 47423579 | 47422135 | chr8 | 34490815 | 34492260 | Inversion |
| Chr3 | 47424946 | 47424078 | chr7 | 15508635 | 15509515 | Inversion |
| Chr3 | 47553634 | 47553852 | chr8 | 14438405 | 14438623 | Inter-translocation |
| Chr3 | 47554477 | 47553846 | chr8 | 11419107 | 11419738 | Inversion |
| Chr3 | 47672576 | 47672927 | chr9 | 30001965 | 30002316 | Inter-translocation |
| Chr3 | 47787069 | 47787615 | chr3 | 22654848 | 22655395 | Intra-translocation |
| Chr3 | 47814946 | 47815221 | chr4 | 37978840 | 37979115 | Inter-translocation |
| Chr3 | 47843606 | 47843329 | chr1 | 25500615 | 25500901 | Inversion |
| Chr3 | 48134376 | 48134214 | chr3 | 13775752 | 13775913 | Inversion |
| Chr3 | 48152952 | 48154629 | chr8 | 11607030 | 11608710 | Inter-translocation |
| Chr3 | 48156692 | 48156355 | chr4 | 674907 | 675244 | Inversion |
| Chr3 | 48161724 | 48160252 | chr3 | 49002702 | 49004160 | Inversion |
| Chr3 | 48169079 | 48168921 | chr8 | 38903979 | 38904136 | Inversion |
| Chr3 | 48178709 | 48179036 | chr9 | 21485274 | 21485602 | Inter-translocation |
| Chr3 | 48231579 | 48231226 | chr6 | 3258842 | 3259195 | Inversion |
| Chr3 | 48285322 | 48285675 | chr8 | 9459096 | 9459452 | Inter-translocation |
| Chr3 | 48371953 | 48374957 | chr8 | 25933220 | 25936244 | Inter-translocation |
| Chr3 | 48395789 | 48395981 | chr9 | 27185249 | 27185441 | Inter-translocation |
| Chr3 | 48398239 | 48399843 | chr8 | 30541769 | 30543374 | Inter-translocation |
| Chr3 | 48404766 | 48398348 | chr9 | 31302096 | 31308543 | Inversion |
| Chr3 | 48418622 | 48418969 | chr8 | 9906817 | 9907165 | Inter-translocation |
| Chr3 | 48494975 | 48494756 | chr7 | 35520799 | 35521015 | Inversion |
| Chr3 | 49074777 | 49075075 | chr8 | 4145760 | 4146058 | Inter-translocation |
| Chr3 | 49277940 | 49277660 | chr9 | 48683570 | 48683850 | Inversion |
| Chr3 | 49450424 | 49450069 | chr9 | 10362878 | 10363233 | Inversion |
| Chr3 | 49561048 | 49560375 | chr4 | 5838567 | 5839242 | Inversion |
| Chr3 | 49859921 | 49861434 | chr2 | 3296455 | 3297968 | Inter-translocation |
| Chr3 | 49865212 | 49861431 | chr3 | 10797395 | 10801184 | Inversion |
| Chr3 | 49866168 | 49868181 | chr8 | 5611256 | 5613260 | Inter-translocation |
| Chr3 | 49884256 | 49881537 | chr6 | 32746094 | 32748822 | Inversion |
| Chr3 | 49898052 | 49898325 | chr3 | 49518582 | 49518855 | Intra-translocation |
| Chr3 | 50151159 | 50163289 | chr5 | 14233697 | 14245826 | Inter-translocation |
| Chr3 | 50207431 | 50208286 | chr9 | 23773567 | 23774451 | Inter-translocation |
| Chr3 | 50451913 | 50455038 | chr8 | 13629472 | 13632605 | Inter-translocation |
| Chr3 | 50454363 | 50455162 | chr3 | 14758953 | 14759750 | Intra-translocation |
| Chr3 | 50458773 | 50458890 | chr3 | 49393214 | 49393334 | Intra-translocation |
| Chr3 | 50459302 | 50459729 | chr9 | 36849699 | 36850135 | Inter-translocation |
| Chr3 | 50500152 | 50499968 | chr2 | 41509546 | 41509731 | Inversion |
| Chr3 | 50616078 | 50615899 | chr8 | 25434016 | 25434194 | Inversion |
| Chr3 | 50748555 | 50748308 | chr8 | 36803541 | 36803792 | Inversion |
| Chr3 | 50748556 | 50748788 | chr8 | 34944214 | 34944445 | Inter-translocation |
| Chr3 | 50862918 | 50863195 | chr8 | 3292855 | 3293130 | Inter-translocation |
| Chr3 | 50924338 | 50924465 | chr6 | 23235969 | 23236096 | Inter-translocation |
| Chr3 | 50951549 | 50951758 | chr5 | 8871577 | 8871786 | Inter-translocation |
| Chr3 | 50952030 | 50951723 | chr8 | 10203069 | 10203373 | Inversion |
| Chr3 | 50962359 | 50956769 | chr5 | 22690873 | 22696463 | Inversion |
| Chr3 | 50964160 | 50960897 | chr6 | 2879530 | 2882791 | Inversion |
| Chr3 | 50971482 | 50962995 | chr5 | 22682388 | 22690876 | Inversion |
| Chr3 | 51047259 | 51047399 | chr9 | 21946978 | 21947119 | Inter-translocation |
| Chr3 | 51053696 | 51053354 | chr9 | 584124 | 584466 | Inversion |
| Chr3 | 51063119 | 51065071 | chr7 | 16198728 | 16200663 | Inter-translocation |
| Chr3 | 51136146 | 51143464 | chr4 | 7590233 | 7597582 | Inter-translocation |
| Chr3 | 51226043 | 51226397 | chr2 | 27850567 | 27850921 | Inter-translocation |
| Chr3 | 51282801 | 51283530 | chr3 | 49988397 | 49989150 | Intra-translocation |
| Chr3 | 51283618 | 51283837 | chr3 | 50018441 | 50018649 | Intra-translocation |
| Chr3 | 51283846 | 51283953 | chr3 | 50019128 | 50019235 | Intra-translocation |
| Chr4 | 25710 | 25542 | chr4 | 51234 | 51402 | Inversion |
| Chr4 | 29854 | 25827 | chr6 | 34456585 | 34460612 | Inversion |
| Chr4 | 227870 | 228147 | chr3 | 14664268 | 14664545 | Inter-translocation |
| Chr4 | 675704 | 675987 | chr4 | 709438 | 709741 | Intra-translocation |
| Chr4 | 678709 | 679446 | chr4 | 712846 | 713574 | Intra-translocation |
| Chr4 | 679626 | 680484 | chr4 | 713572 | 714440 | Intra-translocation |
| Chr4 | 751289 | 751114 | chr4 | 7207425 | 7207598 | Inversion |
| Chr4 | 857109 | 857547 | chr4 | 919359 | 919801 | Intra-translocation |
| Chr4 | 857257 | 857547 | chr4 | 940696 | 940994 | Intra-translocation |
| Chr4 | 860292 | 860829 | chr4 | 918759 | 919296 | Intra-translocation |
| Chr4 | 899655 | 899890 | chr1 | 26592878 | 26593115 | Inter-translocation |
| Chr4 | 955610 | 953787 | chr8 | 10374399 | 10376223 | Inversion |
| Chr4 | 1006036 | 1013721 | chr8 | 15500741 | 15508427 | Inter-translocation |
| Chr4 | 1254529 | 1254871 | chr3 | 46113567 | 46113909 | Inter-translocation |
| Chr4 | 1285463 | 1285338 | chr5 | 15878383 | 15878508 | Inversion |
| Chr4 | 1596339 | 1595979 | chr2 | 30427638 | 30427998 | Inversion |
| Chr4 | 1991228 | 1991463 | chr8 | 22794538 | 22794770 | Inter-translocation |
| Chr4 | 2115274 | 2115928 | chr8 | 6876070 | 6876734 | Inter-translocation |
| Chr4 | 2163097 | 2159145 | chr9 | 21591797 | 21595749 | Inversion |
| Chr4 | 2201817 | 2201566 | chr9 | 53435080 | 53435340 | Inversion |
| Chr4 | 2204287 | 2204578 | chr8 | 22117031 | 22117322 | Inter-translocation |
| Chr4 | 2210760 | 2211090 | chr4 | 2273861 | 2274190 | Intra-translocation |
| Chr4 | 2241002 | 2236168 | chr8 | 11902912 | 11907751 | Inversion |
| Chr4 | 2366554 | 2365189 | chr6 | 2682430 | 2683799 | Inversion |
| Chr4 | 2659931 | 2660844 | chr9 | 40219068 | 40219985 | Inter-translocation |
| Chr4 | 3016486 | 3016373 | chr8 | 5301449 | 5301562 | Inversion |
| Chr4 | 3023430 | 3023686 | chr8 | 25450195 | 25450451 | Inter-translocation |
| Chr4 | 3177355 | 3177070 | chr8 | 11596288 | 11596569 | Inversion |
| Chr4 | 3241744 | 3241396 | chr7 | 28573405 | 28573753 | Inversion |
| Chr4 | 3694002 | 3694350 | chr5 | 8819891 | 8820239 | Inter-translocation |
| Chr4 | 3992267 | 3991902 | chr3 | 6850946 | 6851311 | Inversion |
| Chr4 | 4084084 | 4083745 | chr3 | 8431611 | 8431950 | Inversion |
| Chr4 | 4308010 | 4308285 | chr9 | 54496761 | 54497036 | Inter-translocation |
| Chr4 | 4926703 | 4926348 | chr2 | 44509848 | 44510203 | Inversion |
| Chr4 | 5374339 | 5373278 | chr9 | 40220086 | 40221148 | Inversion |
| Chr4 | 5938466 | 5938185 | chr3 | 22127164 | 22127445 | Inversion |
| Chr4 | 6035719 | 6035498 | chr5 | 15068217 | 15068438 | Inversion |
| Chr4 | 6531557 | 6531768 | chr5 | 15639081 | 15639290 | Inter-translocation |
| Chr4 | 6673630 | 6668125 | chr4 | 2323075 | 2328581 | Inversion |
| Chr4 | 6693705 | 6694786 | chr8 | 11054846 | 11055902 | Inter-translocation |
| Chr4 | 6694780 | 6696159 | chr8 | 11059458 | 11060846 | Inter-translocation |
| Chr4 | 6696298 | 6696557 | chr8 | 11060851 | 11061110 | Inter-translocation |
| Chr4 | 6715242 | 6719233 | chr9 | 19606736 | 19610716 | Inter-translocation |
| Chr4 | 6816189 | 6814104 | chr4 | 7225621 | 7227716 | Inversion |
| Chr4 | 6817999 | 6816932 | chr4 | 7224466 | 7225571 | Inversion |
| Chr4 | 6821513 | 6819134 | chr4 | 7222099 | 7224470 | Inversion |
| Chr4 | 6835637 | 6821504 | chr4 | 7207769 | 7221916 | Inversion |
| Chr4 | 6836612 | 6835680 | chr4 | 7206485 | 7207425 | Inversion |
| Chr4 | 6847327 | 6836632 | chr4 | 7195713 | 7206384 | Inversion |
| Chr4 | 6850354 | 6850455 | chr7 | 30004409 | 30004511 | Inter-translocation |
| Chr4 | 7114473 | 7114087 | chr2 | 41837616 | 41838017 | Inversion |
| Chr4 | 7266788 | 7267206 | chr1 | 30217623 | 30218041 | Inter-translocation |
| Chr4 | 7655698 | 7648159 | chr6 | 28287536 | 28295037 | Inversion |
| Chr4 | 8731076 | 8730969 | chr8 | 38362713 | 38362822 | Inversion |
| Chr4 | 8978377 | 8978723 | chr3 | 50579329 | 50579675 | Inter-translocation |
| Chr4 | 9485744 | 9483380 | chr2 | 3041215 | 3043577 | Inversion |
| Chr4 | 9583026 | 9582853 | chr3 | 8423531 | 8423704 | Inversion |
| Chr4 | 9583189 | 9583464 | chr9 | 40589485 | 40589760 | Inter-translocation |
| Chr4 | 9779672 | 9779440 | chr8 | 26502180 | 26502412 | Inversion |
| Chr4 | 9788183 | 9788404 | chr9 | 34754869 | 34755087 | Inter-translocation |
| Chr4 | 9813022 | 9811107 | chr8 | 6810994 | 6812907 | Inversion |
| Chr4 | 9841184 | 9835539 | chr5 | 14781860 | 14787493 | Inversion |
| Chr4 | 9954488 | 9954401 | chr4 | 10412888 | 10412975 | Inversion |
| Chr4 | 9961501 | 9962327 | chr4 | 7023302 | 7024128 | Intra-translocation |
| Chr4 | 10027115 | 10029563 | chr9 | 31605751 | 31608204 | Inter-translocation |
| Chr4 | 10029295 | 10027114 | chr8 | 4121 | 6303 | Inversion |
| Chr4 | 10031704 | 10032779 | chr8 | 20474145 | 20475217 | Inter-translocation |
| Chr4 | 10066994 | 10067256 | chr8 | 37536184 | 37536446 | Inter-translocation |
| Chr4 | 10383125 | 10382840 | chr8 | 8530113 | 8530386 | Inversion |
| Chr4 | 10383235 | 10383881 | chr3 | 13424810 | 13425454 | Inter-translocation |
| Chr4 | 10660687 | 10657029 | chr5 | 26259 | 29922 | Inversion |
| Chr4 | 10663357 | 10661179 | chr5 | 24077 | 26259 | Inversion |
| Chr4 | 10829221 | 10828870 | chr4 | 3396561 | 3396912 | Inversion |
| Chr4 | 11412983 | 11412676 | chr4 | 5289307 | 5289612 | Inversion |
| Chr4 | 11674379 | 11674526 | chr9 | 9120860 | 9121007 | Inter-translocation |
| Chr4 | 11721121 | 11720767 | chr3 | 4429526 | 4429880 | Inversion |
| Chr4 | 12181639 | 12181336 | chr1 | 10470795 | 10471098 | Inversion |
| Chr4 | 12306648 | 12307000 | chr2 | 42860861 | 42861213 | Inter-translocation |
| Chr4 | 12325793 | 12325613 | chr3 | 25348786 | 25348967 | Inversion |
| Chr4 | 12460790 | 12462524 | chr8 | 10664731 | 10666461 | Inter-translocation |
| Chr4 | 13274156 | 13272912 | chr8 | 5391537 | 5392786 | Inversion |
| Chr4 | 13608654 | 13606094 | chr8 | 9840899 | 9843461 | Inversion |
| Chr4 | 13892874 | 13892647 | chr5 | 15909332 | 15909561 | Inversion |
| Chr4 | 13894621 | 13896607 | chr9 | 34262430 | 34264414 | Inter-translocation |
| Chr4 | 14248776 | 14248580 | chr9 | 41221410 | 41221606 | Inversion |
| Chr4 | 14458943 | 14459412 | chr8 | 3399610 | 3400067 | Inter-translocation |
| Chr4 | 14459261 | 14456348 | chr8 | 4269501 | 4272410 | Inversion |
| Chr4 | 14805830 | 14800795 | chr5 | 15972097 | 15977127 | Inversion |
| Chr4 | 15351592 | 15351181 | chr8 | 2787086 | 2787497 | Inversion |
| Chr4 | 15353017 | 15351502 | chr8 | 2785532 | 2787093 | Inversion |
| Chr4 | 16432873 | 16432688 | chr8 | 3238894 | 3239083 | Inversion |
| Chr4 | 17038261 | 17032636 | chr4 | 16850100 | 16855732 | Inversion |
| Chr4 | 17064704 | 17065045 | chr2 | 3542452 | 3542793 | Inter-translocation |
| Chr4 | 18245495 | 18245891 | chr9 | 12208849 | 12209245 | Inter-translocation |
| Chr4 | 19360886 | 19359896 | chr8 | 3643598 | 3644589 | Inversion |
| Chr4 | 20696454 | 20683772 | chr1 | 25159673 | 25172252 | Inversion |
| Chr4 | 20842998 | 20847288 | chr8 | 19745790 | 19750078 | Inter-translocation |
| Chr4 | 20880346 | 20879642 | chr9 | 30849456 | 30850158 | Inversion |
| Chr4 | 20893859 | 20900646 | chr9 | 38930328 | 38937122 | Inter-translocation |
| Chr4 | 20911983 | 20917085 | chr1 | 17063580 | 17068690 | Inter-translocation |
| Chr4 | 20915839 | 20919533 | chr1 | 17060611 | 17064304 | Inter-translocation |
| Chr4 | 20975625 | 20949742 | chr4 | 31517863 | 31543765 | Inversion |
| Chr4 | 20984998 | 20975665 | chr4 | 31508426 | 31517760 | Inversion |
| Chr4 | 20985707 | 20995109 | chr4 | 31508358 | 31517757 | Intra-translocation |
| Chr4 | 21003167 | 21022799 | chr4 | 31487611 | 31507240 | Intra-translocation |
| Chr4 | 21024638 | 21027708 | chr7 | 7692624 | 7695682 | Inter-translocation |
| Chr4 | 21051586 | 21050415 | chr3 | 22648129 | 22649298 | Inversion |
| Chr4 | 21066228 | 21066115 | chr9 | 27214399 | 27214512 | Inversion |
| Chr4 | 21080104 | 21080389 | chr3 | 49483938 | 49484225 | Inter-translocation |
| Chr4 | 21100279 | 21098360 | chr8 | 29591256 | 29593175 | Inversion |
| Chr4 | 21159500 | 21159636 | chr8 | 22398081 | 22398219 | Inter-translocation |
| Chr4 | 21192529 | 21191164 | chr9 | 26661161 | 26662555 | Inversion |
| Chr4 | 21197993 | 21197653 | chr8 | 4691988 | 4692337 | Inversion |
| Chr4 | 21228217 | 21226826 | chr8 | 12662220 | 12663604 | Inversion |
| Chr4 | 21247980 | 21246415 | chr8 | 4692510 | 4694114 | Inversion |
| Chr4 | 21248674 | 21250064 | chr8 | 13684518 | 13685909 | Inter-translocation |
| Chr4 | 21313521 | 21312364 | chr9 | 30898489 | 30899645 | Inversion |
| Chr4 | 21321260 | 21319096 | chr9 | 30448038 | 30450204 | Inversion |
| Chr4 | 22500900 | 22500982 | chr4 | 2668370 | 2668452 | Intra-translocation |
| Chr4 | 23678523 | 23680318 | chr4 | 23714012 | 23715806 | Intra-translocation |
| Chr4 | 23718067 | 23712485 | chr3 | 13926303 | 13931849 | Inversion |
| Chr4 | 23825244 | 23825048 | chr3 | 50158751 | 50158947 | Inversion |
| Chr4 | 23838454 | 23842124 | chr7 | 7072347 | 7075999 | Inter-translocation |
| Chr4 | 24823261 | 24823613 | chr7 | 26309019 | 26309371 | Inter-translocation |
| Chr4 | 25905162 | 25904949 | chr8 | 17474418 | 17474631 | Inversion |
| Chr4 | 27040659 | 27036610 | chr9 | 44438593 | 44442642 | Inversion |
| Chr4 | 27046665 | 27046595 | chr8 | 3563283 | 3563353 | Inversion |
| Chr4 | 28646625 | 28645775 | chr2 | 42524117 | 42525005 | Inversion |
| Chr4 | 28755941 | 28750335 | chr9 | 22757314 | 22762920 | Inversion |
| Chr4 | 28757427 | 28756009 | chr9 | 22753132 | 22754549 | Inversion |
| Chr4 | 28882489 | 28880417 | chr1 | 25450276 | 25452338 | Inversion |
| Chr4 | 30751607 | 30756117 | chr2 | 2940221 | 2944793 | Inter-translocation |
| Chr4 | 32231955 | 32231326 | chr6 | 21431076 | 21431695 | Inversion |
| Chr4 | 32259836 | 32260111 | chr3 | 49407599 | 49407874 | Inter-translocation |
| Chr4 | 32284094 | 32284450 | chr9 | 55618007 | 55618363 | Inter-translocation |
| Chr4 | 32821061 | 32821341 | chr9 | 3265046 | 3265326 | Inter-translocation |
| Chr4 | 33071169 | 33071518 | chr8 | 22730856 | 22731205 | Inter-translocation |
| Chr4 | 33100764 | 33097505 | chr8 | 6969295 | 6972554 | Inversion |
| Chr4 | 33350905 | 33351260 | chr9 | 52586004 | 52586359 | Inter-translocation |
| Chr4 | 33404868 | 33404518 | chr8 | 30456814 | 30457167 | Inversion |
| Chr4 | 33404950 | 33404826 | chr3 | 46857374 | 46857499 | Inversion |
| Chr4 | 33425313 | 33425592 | chr7 | 22522840 | 22523119 | Inter-translocation |
| Chr4 | 33986543 | 33986183 | chr3 | 27653865 | 27654225 | Inversion |
| Chr4 | 34050250 | 34038395 | chr9 | 19832257 | 19844089 | Inversion |
| Chr4 | 34806723 | 34801765 | chr5 | 23423363 | 23428321 | Inversion |
| Chr4 | 34823588 | 34825639 | chr5 | 15948520 | 15950582 | Inter-translocation |
| Chr4 | 35008251 | 35008611 | chr4 | 35140491 | 35140851 | Intra-translocation |
| Chr4 | 35018325 | 35013267 | chr5 | 3933491 | 3938564 | Inversion |
| Chr4 | 35029026 | 35033062 | chr4 | 35143285 | 35147329 | Intra-translocation |
| Chr4 | 35035840 | 35036172 | chr4 | 35140157 | 35140482 | Intra-translocation |
| Chr4 | 35038072 | 35037008 | chr8 | 12557379 | 12558431 | Inversion |
| Chr4 | 35077045 | 35081160 | chr4 | 35376225 | 35380339 | Intra-translocation |
| Chr4 | 35127158 | 35126877 | chr9 | 34285425 | 34285707 | Inversion |
| Chr4 | 35335762 | 35335988 | chr8 | 2875709 | 2875936 | Inter-translocation |
| Chr4 | 35363555 | 35363661 | chr1 | 25952518 | 25952625 | Inter-translocation |
| Chr4 | 35372726 | 35372315 | chr1 | 26603287 | 26603696 | Inversion |
| Chr4 | 35391423 | 35391546 | chr7 | 30294655 | 30294779 | Inter-translocation |
| Chr4 | 35412350 | 35412076 | chr7 | 22613655 | 22613929 | Inversion |
| Chr4 | 35433254 | 35434094 | chr4 | 35511511 | 35512334 | Intra-translocation |
| Chr4 | 35472099 | 35475318 | chr4 | 35490994 | 35494196 | Intra-translocation |
| Chr4 | 35490835 | 35492812 | chr4 | 35483386 | 35485355 | Intra-translocation |
| Chr4 | 35895531 | 35898021 | chr9 | 31064307 | 31066788 | Inter-translocation |
| Chr4 | 37696473 | 37701342 | chr8 | 4363607 | 4368447 | Inter-translocation |
| Chr4 | 37820898 | 37821179 | chr4 | 36965766 | 36966047 | Intra-translocation |
| Chr4 | 37900019 | 37900368 | chr3 | 11413900 | 11414249 | Inter-translocation |
| Chr4 | 37953550 | 37953798 | chr3 | 8301133 | 8301381 | Inter-translocation |
| Chr4 | 37961486 | 37962278 | chr3 | 5056694 | 5057486 | Inter-translocation |
| Chr4 | 37965444 | 37966054 | chr3 | 5061393 | 5061995 | Inter-translocation |
| Chr4 | 38114669 | 38114950 | chr3 | 49638553 | 49638834 | Inter-translocation |
| Chr4 | 38233901 | 38233554 | chr9 | 12302961 | 12303308 | Inversion |
| Chr4 | 38706799 | 38701597 | chr9 | 39187426 | 39192644 | Inversion |
| Chr4 | 38804292 | 38804720 | chr9 | 19778731 | 19779165 | Inter-translocation |
| Chr4 | 38932796 | 38943784 | chr4 | 38987850 | 38998838 | Intra-translocation |
| Chr4 | 39148009 | 39146552 | chr8 | 16496939 | 16498402 | Inversion |
| Chr4 | 39374834 | 39375046 | chr3 | 5695763 | 5695975 | Inter-translocation |
| Chr4 | 39538853 | 39525642 | chr4 | 39408125 | 39421344 | Inversion |
| Chr4 | 40364141 | 40361967 | chr4 | 21123 | 23304 | Inversion |
| Chr4 | 40366060 | 40369039 | chr8 | 29792531 | 29795510 | Inter-translocation |
| Chr5 | 755806 | 755480 | chr4 | 34079576 | 34079899 | Inversion |
| Chr5 | 822651 | 805771 | chr5 | 795556 | 812416 | Inversion |
| Chr5 | 1561389 | 1559852 | chr6 | 10057083 | 10058620 | Inversion |
| Chr5 | 1600183 | 1608333 | chr5 | 1616885 | 1625031 | Intra-translocation |
| Chr5 | 1719345 | 1722046 | chr2 | 48358943 | 48361642 | Inter-translocation |
| Chr5 | 2340751 | 2339343 | chr9 | 32751923 | 32753332 | Inversion |
| Chr5 | 2345898 | 2350276 | chr8 | 38070170 | 38074562 | Inter-translocation |
| Chr5 | 3087068 | 3086883 | chr2 | 3200119 | 3200304 | Inversion |
| Chr5 | 3957655 | 3957785 | chr9 | 40286395 | 40286523 | Inter-translocation |
| Chr5 | 4131132 | 4131486 | chr1 | 27148482 | 27148836 | Inter-translocation |
| Chr5 | 5376002 | 5375651 | chr9 | 54534967 | 54535318 | Inversion |
| Chr5 | 5619965 | 5621559 | chr8 | 11622627 | 11624218 | Inter-translocation |
| Chr5 | 5915335 | 5915155 | chr4 | 12083519 | 12083700 | Inversion |
| Chr5 | 7085081 | 7085430 | chr9 | 52949484 | 52949833 | Inter-translocation |
| Chr5 | 7844326 | 7842370 | chr5 | 14107662 | 14109617 | Inversion |
| Chr5 | 7920611 | 7920893 | chr9 | 51302230 | 51302512 | Inter-translocation |
| Chr5 | 7961670 | 7960533 | chr8 | 25166515 | 25167653 | Inversion |
| Chr5 | 7965666 | 7966327 | chr8 | 8601101 | 8601792 | Inter-translocation |
| Chr5 | 7966311 | 7966636 | chr8 | 8602091 | 8602419 | Inter-translocation |
| Chr5 | 7966633 | 7966928 | chr8 | 8602569 | 8602864 | Inter-translocation |
| Chr5 | 7993770 | 7992533 | chr5 | 8016689 | 8017938 | Inversion |
| Chr5 | 8010827 | 8010000 | chr5 | 7969632 | 7970442 | Inversion |
| Chr5 | 8011723 | 8011352 | chr9 | 17453603 | 17453975 | Inversion |
| Chr5 | 8022327 | 8021479 | chr5 | 7951800 | 7952648 | Inversion |
| Chr5 | 8025991 | 8026228 | chr5 | 7987350 | 7987587 | Intra-translocation |
| Chr5 | 8026228 | 8026645 | chr5 | 7992564 | 7992982 | Intra-translocation |
| Chr5 | 8027655 | 8028422 | chr5 | 8001294 | 8002060 | Intra-translocation |
| Chr5 | 8029430 | 8033005 | chr5 | 7995405 | 7998884 | Intra-translocation |
| Chr5 | 8034019 | 8033648 | chr2 | 27555773 | 27556143 | Inversion |
| Chr5 | 8034021 | 8034770 | chr5 | 8011336 | 8012076 | Intra-translocation |
| Chr5 | 8034760 | 8034924 | chr5 | 8012571 | 8012738 | Intra-translocation |
| Chr5 | 8035094 | 8036113 | chr5 | 8039787 | 8040804 | Intra-translocation |
| Chr5 | 8037035 | 8037992 | chr5 | 8039656 | 8040605 | Intra-translocation |
| Chr5 | 8241290 | 8240500 | chr8 | 25105110 | 25105898 | Inversion |
| Chr5 | 8517954 | 8518506 | chr5 | 8530808 | 8531359 | Intra-translocation |
| Chr5 | 8526861 | 8527389 | chr5 | 8524453 | 8524973 | Intra-translocation |
| Chr5 | 8561840 | 8560750 | chr5 | 8585680 | 8586769 | Inversion |
| Chr5 | 8570566 | 8568594 | chr5 | 8590923 | 8592881 | Inversion |
| Chr5 | 8580868 | 8583585 | chr5 | 8552004 | 8554665 | Intra-translocation |
| Chr5 | 8583518 | 8584773 | chr5 | 8581587 | 8582837 | Intra-translocation |
| Chr5 | 8585325 | 8585749 | chr5 | 8583145 | 8583568 | Intra-translocation |
| Chr5 | 8586386 | 8586744 | chr5 | 8584188 | 8584564 | Intra-translocation |
| Chr5 | 8588591 | 8589297 | chr5 | 8584570 | 8585267 | Intra-translocation |
| Chr5 | 8602877 | 8599692 | chr5 | 8589594 | 8592789 | Inversion |
| Chr5 | 8603408 | 8602911 | chr5 | 8588408 | 8588905 | Inversion |
| Chr5 | 8875994 | 8876513 | chr5 | 8871047 | 8871578 | Intra-translocation |
| Chr5 | 8900285 | 8900462 | chr8 | 20491816 | 20491993 | Inter-translocation |
| Chr5 | 8906635 | 8908887 | chr5 | 8910226 | 8912453 | Intra-translocation |
| Chr5 | 8920344 | 8922689 | chr5 | 8864002 | 8866354 | Intra-translocation |
| Chr5 | 8925428 | 8927199 | chr5 | 8884653 | 8886455 | Intra-translocation |
| Chr5 | 8935305 | 8939839 | chr5 | 8936374 | 8940874 | Intra-translocation |
| Chr5 | 8936161 | 8940518 | chr5 | 8891794 | 8896166 | Intra-translocation |
| Chr5 | 8947318 | 8947489 | chr5 | 8958787 | 8958958 | Intra-translocation |
| Chr5 | 8990533 | 8990159 | chr3 | 6994892 | 6995265 | Inversion |
| Chr5 | 9006904 | 9010467 | chr8 | 11881377 | 11884945 | Inter-translocation |
| Chr5 | 9139801 | 9140018 | chr3 | 48152936 | 48153152 | Inter-translocation |
| Chr5 | 9144093 | 9144368 | chr3 | 49856266 | 49856541 | Inter-translocation |
| Chr5 | 9144978 | 9144698 | chr5 | 15877632 | 15877912 | Inversion |
| Chr5 | 9146355 | 9146237 | chr5 | 9211374 | 9211492 | Inversion |
| Chr5 | 9149143 | 9150722 | chr2 | 27867594 | 27869130 | Inter-translocation |
| Chr5 | 9157241 | 9157472 | chr3 | 49502712 | 49502943 | Inter-translocation |
| Chr5 | 9231338 | 9228847 | chr9 | 20209139 | 20211626 | Inversion |
| Chr5 | 9231967 | 9231725 | chr3 | 24982518 | 24982760 | Inversion |
| Chr5 | 9232525 | 9232301 | chr3 | 24981931 | 24982155 | Inversion |
| Chr5 | 9285686 | 9286042 | chr1 | 24789639 | 24789995 | Inter-translocation |
| Chr5 | 9298306 | 9299697 | chr5 | 9325119 | 9326461 | Intra-translocation |
| Chr5 | 9299387 | 9299761 | chr5 | 9327561 | 9327934 | Intra-translocation |
| Chr5 | 9299898 | 9300985 | chr5 | 9326503 | 9327544 | Intra-translocation |
| Chr5 | 11619039 | 11618760 | chr7 | 30722567 | 30722846 | Inversion |
| Chr5 | 12834080 | 12827264 | chr9 | 25101887 | 25108693 | Inversion |
| Chr5 | 13639674 | 13640020 | chr8 | 5237763 | 5238109 | Inter-translocation |
| Chr5 | 13754257 | 13753990 | chr7 | 31423678 | 31423946 | Inversion |
| Chr5 | 13936749 | 13936624 | chr9 | 41081484 | 41081609 | Inversion |
| Chr5 | 14198416 | 14198893 | chr2 | 2975608 | 2976088 | Inter-translocation |
| Chr5 | 14202436 | 14202788 | chr4 | 8921586 | 8921938 | Inter-translocation |
| Chr5 | 14267402 | 14266186 | chr4 | 34283327 | 34284572 | Inversion |
| Chr5 | 14462101 | 14463431 | chr1 | 1947011 | 1948320 | Inter-translocation |
| Chr5 | 14592207 | 14594280 | chr8 | 4538687 | 4540762 | Inter-translocation |
| Chr5 | 14891303 | 14884377 | chr8 | 12667153 | 12674099 | Inversion |
| Chr5 | 14963120 | 14961889 | chr8 | 19409110 | 19410349 | Inversion |
| Chr5 | 15033902 | 15033560 | chr3 | 49540231 | 49540573 | Inversion |
| Chr5 | 15575369 | 15575012 | chr9 | 49181387 | 49181744 | Inversion |
| Chr5 | 15658018 | 15657272 | chr8 | 40341626 | 40342369 | Inversion |
| Chr5 | 15961611 | 15960760 | chr9 | 34264407 | 34265255 | Inversion |
| Chr5 | 16089957 | 16088316 | chr7 | 27021100 | 27022743 | Inversion |
| Chr5 | 16414867 | 16433073 | chr5 | 18878219 | 18896420 | Intra-translocation |
| Chr5 | 16743977 | 16745001 | chr5 | 16731667 | 16732690 | Intra-translocation |
| Chr5 | 16745435 | 16758402 | chr5 | 16733205 | 16746146 | Intra-translocation |
| Chr5 | 16837952 | 16834740 | chr5 | 21352494 | 21355705 | Inversion |
| Chr5 | 18070430 | 18070922 | chr8 | 18989633 | 18990125 | Inter-translocation |
| Chr5 | 18185766 | 18200390 | chr4 | 4918291 | 4932916 | Inter-translocation |
| Chr5 | 18485127 | 18482840 | chr8 | 10995708 | 10998007 | Inversion |
| Chr5 | 18498418 | 18492783 | chr2 | 18563005 | 18568646 | Inversion |
| Chr5 | 18600916 | 18602145 | chr9 | 31104806 | 31106033 | Inter-translocation |
| Chr5 | 18634497 | 18634665 | chr9 | 19605537 | 19605707 | Inter-translocation |
| Chr5 | 18654551 | 18654316 | chr8 | 4504477 | 4504712 | Inversion |
| Chr5 | 18662144 | 18661746 | chr8 | 4483353 | 4483751 | Inversion |
| Chr5 | 18695846 | 18687794 | chr9 | 31372466 | 31380495 | Inversion |
| Chr5 | 18708098 | 18706828 | chr8 | 7121379 | 7122660 | Inversion |
| Chr5 | 18708935 | 18708441 | chr8 | 7120890 | 7121384 | Inversion |
| Chr5 | 18710980 | 18709281 | chr8 | 7119188 | 7120893 | Inversion |
| Chr5 | 18751273 | 18750133 | chr8 | 7050405 | 7051546 | Inversion |
| Chr5 | 18798863 | 18795690 | chr9 | 31648444 | 31651619 | Inversion |
| Chr5 | 18820597 | 18820451 | chr1 | 25975035 | 25975182 | Inversion |
| Chr5 | 18838648 | 18837600 | chr3 | 49560153 | 49561200 | Inversion |
| Chr5 | 18841386 | 18839353 | chr3 | 49558160 | 49560159 | Inversion |
| Chr5 | 18855685 | 18856817 | chr2 | 27742389 | 27743524 | Inter-translocation |
| Chr5 | 18908759 | 18908429 | chr9 | 27492077 | 27492410 | Inversion |
| Chr5 | 18911344 | 18910293 | chr3 | 7113892 | 7114945 | Inversion |
| Chr5 | 18924939 | 18922992 | chr5 | 25509114 | 25511051 | Inversion |
| Chr5 | 18955680 | 18959254 | chr5 | 19913011 | 19916579 | Intra-translocation |
| Chr5 | 19858148 | 19857838 | chr5 | 9320911 | 9321221 | Inversion |
| Chr5 | 20358426 | 20350107 | chr5 | 19938556 | 19946878 | Inversion |
| Chr5 | 20359120 | 20356315 | chr5 | 19937305 | 19940113 | Inversion |
| Chr5 | 20431038 | 20426891 | chr9 | 30879214 | 30883367 | Inversion |
| Chr5 | 21412062 | 21412505 | chr2 | 23172037 | 23172478 | Inter-translocation |
| Chr5 | 22194342 | 22189897 | chr3 | 39051515 | 39055963 | Inversion |
| Chr5 | 22195822 | 22199708 | chr5 | 16445632 | 16449518 | Intra-translocation |
| Chr5 | 22379188 | 22378781 | chr5 | 23374057 | 23374464 | Inversion |
| Chr5 | 22464019 | 22463665 | chr5 | 23542026 | 23542380 | Inversion |
| Chr5 | 22486891 | 22486194 | chr6 | 2513283 | 2513980 | Inversion |
| Chr5 | 22508573 | 22508997 | chr5 | 23519988 | 23520407 | Intra-translocation |
| Chr5 | 22602534 | 22602186 | chr4 | 7103204 | 7103552 | Inversion |
| Chr5 | 22610642 | 22608337 | chr5 | 23603321 | 23605618 | Inversion |
| Chr5 | 22688740 | 22689333 | chr9 | 24259730 | 24260317 | Inter-translocation |
| Chr5 | 22689040 | 22691032 | chr9 | 27281444 | 27283421 | Inter-translocation |
| Chr5 | 23134677 | 23135793 | chr1 | 8181317 | 8182436 | Inter-translocation |
| Chr5 | 23419827 | 23418421 | chr3 | 4925588 | 4926995 | Inversion |
| Chr5 | 23500715 | 23495346 | chr8 | 29345707 | 29351083 | Inversion |
| Chr5 | 23509341 | 23500828 | chr8 | 29336947 | 29345431 | Inversion |
| Chr5 | 23654538 | 23654440 | chr5 | 24543212 | 24543310 | Inversion |
| Chr5 | 23684313 | 23682362 | chr8 | 19184655 | 19186609 | Inversion |
| Chr5 | 23714317 | 23719083 | chr8 | 25230571 | 25235334 | Inter-translocation |
| Chr5 | 23719701 | 23714318 | chr8 | 12024136 | 12029568 | Inversion |
| Chr5 | 23774170 | 23773818 | chr9 | 57114685 | 57115037 | Inversion |
| Chr5 | 24062367 | 24062720 | chr1 | 16036257 | 16036610 | Inter-translocation |
| Chr5 | 24184676 | 24185110 | chr9 | 23282185 | 23282621 | Inter-translocation |
| Chr5 | 24193308 | 24193066 | chr8 | 36841371 | 36841613 | Inversion |
| Chr5 | 24196404 | 24195341 | chr1 | 14695393 | 14696456 | Inversion |
| Chr5 | 24215029 | 24215149 | chr8 | 5710028 | 5710142 | Inter-translocation |
| Chr5 | 24275373 | 24272842 | chr9 | 31101799 | 31104316 | Inversion |
| Chr5 | 24289850 | 24288084 | chr8 | 16338493 | 16340282 | Inversion |
| Chr5 | 24296783 | 24295118 | chr8 | 25673922 | 25675593 | Inversion |
| Chr5 | 24300893 | 24299939 | chr7 | 3270934 | 3271906 | Inversion |
| Chr5 | 24313912 | 24314287 | chr9 | 22233415 | 22233776 | Inter-translocation |
| Chr5 | 24344516 | 24345154 | chr8 | 20192747 | 20193386 | Inter-translocation |
| Chr5 | 24368409 | 24368104 | chr8 | 9526551 | 9526857 | Inversion |
| Chr5 | 24378545 | 24373140 | chr9 | 535087 | 540487 | Inversion |
| Chr5 | 24380002 | 24380886 | chr7 | 22723735 | 22724614 | Inter-translocation |
| Chr5 | 24870499 | 24869305 | chr9 | 22137795 | 22138992 | Inversion |
| Chr5 | 24931172 | 24930493 | chr8 | 25329366 | 25330021 | Inversion |
| Chr5 | 24959338 | 24963933 | chr5 | 8505968 | 8510530 | Intra-translocation |
| Chr5 | 24993242 | 24993350 | chr9 | 29917697 | 29917805 | Inter-translocation |
| Chr5 | 26058752 | 26064962 | chr8 | 38032509 | 38038719 | Inter-translocation |
| Chr5 | 26065562 | 26065739 | chr2 | 31320591 | 31320770 | Inter-translocation |
| Chr5 | 26066183 | 26066458 | chr8 | 38512005 | 38512280 | Inter-translocation |
| Chr5 | 26079146 | 26066627 | chr5 | 32245008 | 32257530 | Inversion |
| Chr5 | 26081843 | 26079466 | chr5 | 32242217 | 32244594 | Inversion |
| Chr5 | 26176727 | 26082281 | chr5 | 32147672 | 32242115 | Inversion |
| Chr5 | 26311760 | 26176642 | chr5 | 32012363 | 32147483 | Inversion |
| Chr5 | 26427034 | 26311777 | chr5 | 31896620 | 32011875 | Inversion |
| Chr5 | 26563887 | 26427103 | chr5 | 31759717 | 31896494 | Inversion |
| Chr5 | 26603070 | 26564029 | chr5 | 31720335 | 31759376 | Inversion |
| Chr5 | 26693751 | 26603294 | chr5 | 31629692 | 31720122 | Inversion |
| Chr5 | 27871317 | 27871458 | chr9 | 21335339 | 21335479 | Inter-translocation |
| Chr5 | 27888060 | 27888420 | chr8 | 3164839 | 3165198 | Inter-translocation |
| Chr5 | 27928534 | 27928749 | chr5 | 16271146 | 16271360 | Intra-translocation |
| Chr5 | 28527816 | 28522696 | chr1 | 15579585 | 15584701 | Inversion |
| Chr5 | 29844943 | 29846022 | chr1 | 13173987 | 13175065 | Inter-translocation |
| Chr5 | 29844987 | 29850046 | chr9 | 9225232 | 9230290 | Inter-translocation |
| Chr5 | 30479714 | 30480135 | chr4 | 10329827 | 10330248 | Inter-translocation |
| Chr5 | 31432093 | 31432260 | chr7 | 26365670 | 26365837 | Inter-translocation |
| Chr5 | 31432249 | 31432438 | chr7 | 26366136 | 26366325 | Inter-translocation |
| Chr5 | 32875997 | 32880986 | chr5 | 34086637 | 34091626 | Intra-translocation |
| Chr5 | 33588149 | 33588492 | chr3 | 44494985 | 44495328 | Inter-translocation |
| Chr5 | 34056802 | 34056645 | chr3 | 23267334 | 23267492 | Inversion |
| Chr5 | 34952197 | 34951939 | chr2 | 23558387 | 23558649 | Inversion |
| Chr5 | 36117810 | 36118031 | chr5 | 7914321 | 7914541 | Intra-translocation |
| Chr5 | 36506632 | 36506343 | chr7 | 26498988 | 26499276 | Inversion |
| Chr5 | 37486826 | 37486563 | chr8 | 10538992 | 10539255 | Inversion |
| Chr5 | 37698082 | 37697865 | chr2 | 45321554 | 45321772 | Inversion |
| Chr5 | 38444876 | 38440566 | chr6 | 28616022 | 28620314 | Inversion |
| Chr5 | 38998983 | 38999270 | chr8 | 25968688 | 25968975 | Inter-translocation |
| Chr5 | 39616833 | 39628857 | chr5 | 40720072 | 40732095 | Intra-translocation |
| Chr5 | 41972468 | 41973520 | chr5 | 43119385 | 43120436 | Intra-translocation |
| Chr5 | 42456320 | 42463645 | chr5 | 43601626 | 43608960 | Intra-translocation |
| Chr5 | 43938618 | 43938248 | chr7 | 23046475 | 23046846 | Inversion |
| Chr5 | 44197792 | 44208773 | chr5 | 45346127 | 45357108 | Intra-translocation |
| Chr5 | 44484908 | 44471573 | chr5 | 45592736 | 45606070 | Inversion |
| Chr5 | 45205217 | 45206145 | chr6 | 32981181 | 32982134 | Inter-translocation |
| Chr5 | 45362453 | 45362735 | chr2 | 46366482 | 46366764 | Inter-translocation |
| Chr5 | 45797485 | 45802418 | chr5 | 46866428 | 46871373 | Intra-translocation |
| Chr6 | 1000883 | 1000270 | chr2 | 20202005 | 20202619 | Inversion |
| Chr6 | 1001626 | 1003887 | chr6 | 1010008 | 1012263 | Intra-translocation |
| Chr6 | 1007582 | 1003888 | chr8 | 20786851 | 20790549 | Inversion |
| Chr6 | 1009860 | 1010275 | chr9 | 32770998 | 32771419 | Inter-translocation |
| Chr6 | 1019756 | 1010276 | chr8 | 20777382 | 20786858 | Inversion |
| Chr6 | 1036734 | 1033868 | chr9 | 32590251 | 32593117 | Inversion |
| Chr6 | 1037536 | 1037825 | chr8 | 27765947 | 27766236 | Inter-translocation |
| Chr6 | 1043174 | 1044957 | chr6 | 990753 | 992516 | Intra-translocation |
| Chr6 | 1045693 | 1046636 | chr6 | 1005781 | 1006737 | Intra-translocation |
| Chr6 | 1046758 | 1047141 | chr6 | 1006903 | 1007312 | Intra-translocation |
| Chr6 | 1047603 | 1049793 | chr6 | 965032 | 967223 | Intra-translocation |
| Chr6 | 1051111 | 1051607 | chr8 | 7891230 | 7891722 | Inter-translocation |
| Chr6 | 1057715 | 1059726 | chr6 | 986906 | 988893 | Intra-translocation |
| Chr6 | 1064548 | 1064715 | chr6 | 1007802 | 1007971 | Intra-translocation |
| Chr6 | 1065371 | 1066706 | chr6 | 1008406 | 1009756 | Intra-translocation |
| Chr6 | 1066706 | 1070400 | chr6 | 973430 | 977102 | Intra-translocation |
| Chr6 | 1066787 | 1071613 | chr6 | 983791 | 988562 | Intra-translocation |
| Chr6 | 1134785 | 1129274 | chr8 | 11979152 | 11984663 | Inversion |
| Chr6 | 1179236 | 1184563 | chr8 | 10410776 | 10416066 | Inter-translocation |
| Chr6 | 1233281 | 1233536 | chr9 | 23766539 | 23766793 | Inter-translocation |
| Chr6 | 1244043 | 1244350 | chr7 | 22893234 | 22893543 | Inter-translocation |
| Chr6 | 1254515 | 1254263 | chr6 | 3154642 | 3154891 | Inversion |
| Chr6 | 1578920 | 1578238 | chr6 | 1556362 | 1557054 | Inversion |
| Chr6 | 1579484 | 1579024 | chr6 | 1553841 | 1554299 | Inversion |
| Chr6 | 1579780 | 1579488 | chr6 | 1553145 | 1553436 | Inversion |
| Chr6 | 1580023 | 1580654 | chr4 | 7406938 | 7407568 | Inter-translocation |
| Chr6 | 1581407 | 1581220 | chr6 | 1550377 | 1550564 | Inversion |
| Chr6 | 1586222 | 1582921 | chr6 | 1546624 | 1549935 | Inversion |
| Chr6 | 1591902 | 1591521 | chr6 | 1542717 | 1543100 | Inversion |
| Chr6 | 1592268 | 1591902 | chr6 | 1542177 | 1542562 | Inversion |
| Chr6 | 1611513 | 1611198 | chr7 | 30880887 | 30881213 | Inversion |
| Chr6 | 1613480 | 1613392 | chr1 | 14626785 | 14626873 | Inversion |
| Chr6 | 1822897 | 1825387 | chr6 | 1698488 | 1700978 | Intra-translocation |
| Chr6 | 1827823 | 1827942 | chr8 | 5061766 | 5061882 | Inter-translocation |
| Chr6 | 1831516 | 1834793 | chr6 | 1689698 | 1692982 | Intra-translocation |
| Chr6 | 1981510 | 1987537 | chr6 | 1871073 | 1877100 | Intra-translocation |
| Chr6 | 1994016 | 1998873 | chr6 | 1877982 | 1882849 | Intra-translocation |
| Chr6 | 2046926 | 2059123 | chr6 | 1841912 | 1854088 | Intra-translocation |
| Chr6 | 2488793 | 2488447 | chr3 | 50318819 | 50319167 | Inversion |
| Chr6 | 2649862 | 2650350 | chr8 | 2499537 | 2500017 | Inter-translocation |
| Chr6 | 2656692 | 2657843 | chr7 | 30002148 | 30003291 | Inter-translocation |
| Chr6 | 2664851 | 2661680 | chr5 | 14109685 | 14112874 | Inversion |
| Chr6 | 2665913 | 2665061 | chr9 | 20231266 | 20232119 | Inversion |
| Chr6 | 2726698 | 2726347 | chr9 | 40583093 | 40583444 | Inversion |
| Chr6 | 2788428 | 2783759 | chr8 | 12730934 | 12735635 | Inversion |
| Chr6 | 2800970 | 2801097 | chr8 | 5187442 | 5187569 | Inter-translocation |
| Chr6 | 2804909 | 2804984 | chr6 | 2661349 | 2661424 | Intra-translocation |
| Chr6 | 2836935 | 2838415 | chr6 | 2656119 | 2657598 | Intra-translocation |
| Chr6 | 2838687 | 2842028 | chr6 | 2657895 | 2661271 | Intra-translocation |
| Chr6 | 2841900 | 2842095 | chr6 | 2645526 | 2645709 | Intra-translocation |
| Chr6 | 2842179 | 2843402 | chr6 | 2620891 | 2622115 | Intra-translocation |
| Chr6 | 2842410 | 2843814 | chr6 | 2645853 | 2647256 | Intra-translocation |
| Chr6 | 2843964 | 2844655 | chr6 | 2646831 | 2647521 | Intra-translocation |
| Chr6 | 2844612 | 2854360 | chr6 | 2647334 | 2657104 | Intra-translocation |
| Chr6 | 2951613 | 2952015 | chr9 | 20095277 | 20095679 | Inter-translocation |
| Chr6 | 3079595 | 3080175 | chr6 | 2904340 | 2904893 | Intra-translocation |
| Chr6 | 3080177 | 3085789 | chr6 | 2898253 | 2903899 | Intra-translocation |
| Chr6 | 3107766 | 3112547 | chr6 | 2920957 | 2925640 | Intra-translocation |
| Chr6 | 3125138 | 3127822 | chr6 | 2904818 | 2907478 | Intra-translocation |
| Chr6 | 3285448 | 3285545 | chr2 | 46161634 | 46161730 | Inter-translocation |
| Chr6 | 3330009 | 3329479 | chr6 | 3177992 | 3178522 | Inversion |
| Chr6 | 3339636 | 3338914 | chr6 | 3232785 | 3233522 | Inversion |
| Chr6 | 3340737 | 3340391 | chr9 | 32364869 | 32365215 | Inversion |
| Chr6 | 3348803 | 3348999 | chr9 | 35998056 | 35998252 | Inter-translocation |
| Chr6 | 3565181 | 3565003 | chr8 | 23415666 | 23415844 | Inversion |
| Chr6 | 4029091 | 4028552 | chr6 | 2701842 | 2702382 | Inversion |
| Chr6 | 4031674 | 4029210 | chr6 | 2699366 | 2701842 | Inversion |
| Chr6 | 4032960 | 4031687 | chr6 | 2697704 | 2699016 | Inversion |
| Chr6 | 4034556 | 4032950 | chr6 | 2695609 | 2697225 | Inversion |
| Chr6 | 4043630 | 4034555 | chr6 | 2685261 | 2694288 | Inversion |
| Chr6 | 4045303 | 4043833 | chr6 | 2683808 | 2685255 | Inversion |
| Chr6 | 4052895 | 4049725 | chr6 | 2679243 | 2682429 | Inversion |
| Chr6 | 4055316 | 4053163 | chr6 | 2676993 | 2679142 | Inversion |
| Chr6 | 4058495 | 4055412 | chr6 | 2673912 | 2676992 | Inversion |
| Chr6 | 4078014 | 4069405 | chr6 | 2661479 | 2670058 | Inversion |
| Chr6 | 4078298 | 4078092 | chr6 | 2645481 | 2645688 | Inversion |
| Chr6 | 4085926 | 4078574 | chr6 | 2638179 | 2645480 | Inversion |
| Chr6 | 4620449 | 4620859 | chr6 | 21431731 | 21432135 | Intra-translocation |
| Chr6 | 4827012 | 4827327 | chr8 | 23532068 | 23532383 | Inter-translocation |
| Chr6 | 5825155 | 5842309 | chr6 | 5484309 | 5501432 | Intra-translocation |
| Chr6 | 5877227 | 5878085 | chr6 | 5524692 | 5525549 | Intra-translocation |
| Chr6 | 5880732 | 5883698 | chr6 | 5520784 | 5523764 | Intra-translocation |
| Chr6 | 6426764 | 6427772 | chr8 | 6587241 | 6588249 | Inter-translocation |
| Chr6 | 6427542 | 6431818 | chr8 | 6587752 | 6592028 | Inter-translocation |
| Chr6 | 6428038 | 6432079 | chr8 | 7530222 | 7534261 | Inter-translocation |
| Chr6 | 6437991 | 6433137 | chr3 | 25111593 | 25116465 | Inversion |
| Chr6 | 6538609 | 6538746 | chr9 | 41222643 | 41222780 | Inter-translocation |
| Chr6 | 6539169 | 6538967 | chr8 | 30264754 | 30264954 | Inversion |
| Chr6 | 7278264 | 7255789 | chr9 | 38660172 | 38682646 | Inversion |
| Chr6 | 7294742 | 7277612 | chr9 | 38624506 | 38641638 | Inversion |
| Chr6 | 7296651 | 7295143 | chr9 | 38622445 | 38623953 | Inversion |
| Chr6 | 7303117 | 7307345 | chr9 | 34549431 | 34553670 | Inter-translocation |
| Chr6 | 7307249 | 7301624 | chr9 | 38610767 | 38616394 | Inversion |
| Chr6 | 7323147 | 7307424 | chr9 | 38595015 | 38610747 | Inversion |
| Chr6 | 7660258 | 7660984 | chr6 | 7231583 | 7232334 | Intra-translocation |
| Chr6 | 8115390 | 8113290 | chr4 | 38631117 | 38633216 | Inversion |
| Chr6 | 8117527 | 8116315 | chr4 | 38628980 | 38630192 | Inversion |
| Chr6 | 8946995 | 8946313 | chr8 | 10420208 | 10420887 | Inversion |
| Chr6 | 9089700 | 9090059 | chr8 | 20824721 | 20825080 | Inter-translocation |
| Chr6 | 9791384 | 9790744 | chr2 | 22906879 | 22907518 | Inversion |
| Chr6 | 9927664 | 9925589 | chr9 | 27281362 | 27283421 | Inversion |
| Chr6 | 10143076 | 10144860 | chr6 | 9711874 | 9713654 | Intra-translocation |
| Chr6 | 11661441 | 11659609 | chr9 | 29969569 | 29971400 | Inversion |
| Chr6 | 11813081 | 11813582 | chr3 | 24255594 | 24256097 | Inter-translocation |
| Chr6 | 12070785 | 12070376 | chr8 | 25477154 | 25477563 | Inversion |
| Chr6 | 12437901 | 12437681 | chr8 | 4057572 | 4057792 | Inversion |
| Chr6 | 12443078 | 12438027 | chr8 | 4052523 | 4057572 | Inversion |
| Chr6 | 12629063 | 12623916 | chr9 | 30099525 | 30104672 | Inversion |
| Chr6 | 13226613 | 13218370 | chr8 | 17762095 | 17770339 | Inversion |
| Chr6 | 13888122 | 13887643 | chr9 | 26834169 | 26834648 | Inversion |
| Chr6 | 14165496 | 14163896 | chr8 | 7489992 | 7491594 | Inversion |
| Chr6 | 14496279 | 14491326 | chr3 | 35694917 | 35699889 | Inversion |
| Chr6 | 16008781 | 16008620 | chr4 | 5637621 | 5637782 | Inversion |
| Chr6 | 17019217 | 17017406 | chr9 | 30855350 | 30857171 | Inversion |
| Chr6 | 17866474 | 17913024 | chr6 | 22203465 | 22250005 | Intra-translocation |
| Chr6 | 18044151 | 18043697 | chr9 | 33504015 | 33504458 | Inversion |
| Chr6 | 18089721 | 18088525 | chr8 | 20227795 | 20228982 | Inversion |
| Chr6 | 18106544 | 18107338 | chr1 | 26272021 | 26272813 | Inter-translocation |
| Chr6 | 18524389 | 18516267 | chr8 | 10172354 | 10180518 | Inversion |
| Chr6 | 18962707 | 18956657 | chr8 | 3008817 | 3014855 | Inversion |
| Chr6 | 19350781 | 19350556 | chr8 | 12742134 | 12742359 | Inversion |
| Chr6 | 19651267 | 19647703 | chr1 | 26412944 | 26416504 | Inversion |
| Chr6 | 19950011 | 19949533 | chr8 | 10281125 | 10281600 | Inversion |
| Chr6 | 20634209 | 20634667 | chr8 | 13008669 | 13009126 | Inter-translocation |
| Chr6 | 20992020 | 21013497 | chr6 | 20347534 | 20369011 | Intra-translocation |
| Chr6 | 21035254 | 21057290 | chr6 | 20325497 | 20347533 | Intra-translocation |
| Chr6 | 22679951 | 22679813 | chr3 | 8157791 | 8157929 | Inversion |
| Chr6 | 22680508 | 22680276 | chr8 | 38367821 | 38368053 | Inversion |
| Chr6 | 22683142 | 22685607 | chr9 | 29194460 | 29196930 | Inter-translocation |
| Chr6 | 22818007 | 22818469 | chr6 | 22185531 | 22185991 | Intra-translocation |
| Chr6 | 22820127 | 22818623 | chr9 | 36817467 | 36818971 | Inversion |
| Chr6 | 22830685 | 22820126 | chr9 | 36818261 | 36828783 | Inversion |
| Chr6 | 22862008 | 22866452 | chr6 | 17447231 | 17451670 | Intra-translocation |
| Chr6 | 22905623 | 22908847 | chr6 | 17480785 | 17484007 | Intra-translocation |
| Chr6 | 24153880 | 24151008 | chr8 | 20798607 | 20801479 | Inversion |
| Chr6 | 24154232 | 24153876 | chr3 | 44824847 | 44825195 | Inversion |
| Chr6 | 24633827 | 24634678 | chr8 | 14327623 | 14328468 | Inter-translocation |
| Chr6 | 24737088 | 24732742 | chr5 | 16296174 | 16300522 | Inversion |
| Chr6 | 24774507 | 24773392 | chr8 | 19993972 | 19995087 | Inversion |
| Chr6 | 24774788 | 24774263 | chr8 | 3222796 | 3223323 | Inversion |
| Chr6 | 25076206 | 25076357 | chr4 | 911710 | 911862 | Inter-translocation |
| Chr6 | 25200880 | 25196394 | chr9 | 32484218 | 32488702 | Inversion |
| Chr6 | 25584696 | 25586209 | chr5 | 15147376 | 15148907 | Inter-translocation |
| Chr6 | 26203798 | 26202751 | chr2 | 4440037 | 4441086 | Inversion |
| Chr6 | 26676109 | 26675042 | chr3 | 13325082 | 13326153 | Inversion |
| Chr6 | 26807424 | 26807642 | chr3 | 49975870 | 49976090 | Inter-translocation |
| Chr6 | 26882372 | 26882660 | chr3 | 14108449 | 14108736 | Inter-translocation |
| Chr6 | 27457650 | 27457765 | chr6 | 26759394 | 26759509 | Intra-translocation |
| Chr6 | 27492584 | 27492332 | chr5 | 8492188 | 8492441 | Inversion |
| Chr6 | 27537392 | 27536570 | chr8 | 29630004 | 29630818 | Inversion |
| Chr6 | 27664903 | 27669702 | chr8 | 37548739 | 37553600 | Inter-translocation |
| Chr6 | 28170940 | 28171008 | chr4 | 2408332 | 2408400 | Inter-translocation |
| Chr6 | 28324563 | 28320189 | chr8 | 19336159 | 19340529 | Inversion |
| Chr6 | 28912495 | 28911943 | chr6 | 5676966 | 5677518 | Inversion |
| Chr6 | 29287785 | 29311819 | chr6 | 28581622 | 28605699 | Intra-translocation |
| Chr6 | 29495267 | 29495478 | chr3 | 23980983 | 23981193 | Inter-translocation |
| Chr6 | 29634832 | 29635249 | chr8 | 29540207 | 29540622 | Inter-translocation |
| Chr6 | 29868964 | 29869131 | chr1 | 26253608 | 26253775 | Inter-translocation |
| Chr6 | 30298862 | 30319306 | chr6 | 29640834 | 29661251 | Intra-translocation |
| Chr6 | 30339079 | 30346731 | chr6 | 29603576 | 29611213 | Intra-translocation |
| Chr6 | 30984656 | 30985507 | chr3 | 14676690 | 14677540 | Inter-translocation |
| Chr6 | 31070698 | 31070430 | chr1 | 2644416 | 2644684 | Inversion |
| Chr6 | 31273013 | 31272699 | chr7 | 27485087 | 27485401 | Inversion |
| Chr6 | 31495256 | 31494911 | chr9 | 40667614 | 40667959 | Inversion |
| Chr6 | 31786714 | 31787788 | chr9 | 17242825 | 17243842 | Inter-translocation |
| Chr6 | 31790105 | 31790429 | chr5 | 6983988 | 6984312 | Inter-translocation |
| Chr6 | 32267302 | 32266947 | chr3 | 25507044 | 25507399 | Inversion |
| Chr6 | 32529087 | 32528796 | chr3 | 13670207 | 13670498 | Inversion |
| Chr6 | 32691485 | 32691315 | chr9 | 19095555 | 19095724 | Inversion |
| Chr6 | 32833392 | 32832366 | chr4 | 5857227 | 5858246 | Inversion |
| Chr6 | 32835178 | 32835426 | chr7 | 30405157 | 30405405 | Inter-translocation |
| Chr6 | 32837021 | 32835723 | chr9 | 25515349 | 25516648 | Inversion |
| Chr6 | 32865029 | 32863068 | chr3 | 50031050 | 50033002 | Inversion |
| Chr6 | 33456925 | 33457324 | chr8 | 27797545 | 27797946 | Inter-translocation |
| Chr6 | 33519037 | 33518690 | chr3 | 14236419 | 14236766 | Inversion |
| Chr6 | 33603530 | 33600344 | chr6 | 33409312 | 33412502 | Inversion |
| Chr6 | 33737371 | 33737729 | chr9 | 53767050 | 53767408 | Inter-translocation |
| Chr6 | 33739271 | 33738761 | chr5 | 16041541 | 16042051 | Inversion |
| Chr6 | 33774526 | 33774190 | chr9 | 22674778 | 22675114 | Inversion |
| Chr6 | 33857558 | 33862318 | chr6 | 35847271 | 35852014 | Intra-translocation |
| Chr6 | 34102953 | 34103247 | chr8 | 20153429 | 20153723 | Inter-translocation |
| Chr6 | 34120725 | 34119729 | chr6 | 35895195 | 35896197 | Inversion |
| Chr6 | 34296747 | 34295973 | chr6 | 34284983 | 34285772 | Inversion |
| Chr6 | 34305205 | 34305650 | chr6 | 33567060 | 33567503 | Intra-translocation |
| Chr6 | 34577516 | 34577604 | chr4 | 5899196 | 5899284 | Inter-translocation |
| Chr6 | 34670926 | 34670613 | chr3 | 23267346 | 23267650 | Inversion |
| Chr6 | 34868828 | 34869058 | chr2 | 46161302 | 46161532 | Inter-translocation |
| Chr6 | 34985655 | 34986010 | chr6 | 34252417 | 34252768 | Intra-translocation |
| Chr6 | 35052475 | 35052378 | chr9 | 36530076 | 36530173 | Inversion |
| Chr6 | 35147536 | 35145233 | chr6 | 34537861 | 34540176 | Inversion |
| Chr6 | 35181549 | 35185160 | chr6 | 34451256 | 34454849 | Intra-translocation |
| Chr6 | 35220355 | 35219169 | chr6 | 34489013 | 34490185 | Inversion |
| Chr6 | 35226337 | 35227793 | chr6 | 34534566 | 34536082 | Intra-translocation |
| Chr6 | 35227804 | 35231722 | chr6 | 34536242 | 34540123 | Intra-translocation |
| Chr6 | 35324582 | 35325962 | chr8 | 29334130 | 29335514 | Inter-translocation |
| Chr6 | 35331689 | 35331355 | chr8 | 8823520 | 8823854 | Inversion |
| Chr6 | 35336334 | 35335475 | chr7 | 31462887 | 31463748 | Inversion |
| Chr6 | 35474901 | 35475244 | chr9 | 57540597 | 57540940 | Inter-translocation |
| Chr6 | 35488503 | 35484614 | chr6 | 34528365 | 34532227 | Inversion |
| Chr6 | 35743676 | 35744026 | chr6 | 3492140 | 3492488 | Intra-translocation |
| Chr6 | 36238189 | 36237924 | chr4 | 232815 | 233081 | Inversion |
| Chr6 | 36318732 | 36319996 | chr1 | 900890 | 902179 | Inter-translocation |
| Chr6 | 36318735 | 36317324 | chr6 | 35984751 | 35986161 | Inversion |
| Chr6 | 36324961 | 36326416 | chr3 | 50651110 | 50652567 | Inter-translocation |
| Chr6 | 36333712 | 36331549 | chr8 | 28 | 2188 | Inversion |
| Chr7 | 580563 | 574103 | chr7 | 739040 | 745498 | Inversion |
| Chr7 | 2203977 | 2199713 | chr8 | 25315676 | 25319947 | Inversion |
| Chr7 | 2770308 | 2772241 | chr8 | 3255556 | 3257492 | Inter-translocation |
| Chr7 | 3151350 | 3150991 | chr4 | 8847715 | 8848075 | Inversion |
| Chr7 | 3775294 | 3770174 | chr3 | 46271352 | 46276447 | Inversion |
| Chr7 | 3781131 | 3776807 | chr8 | 14332031 | 14336334 | Inversion |
| Chr7 | 4444826 | 4445109 | chr2 | 42234305 | 42234588 | Inter-translocation |
| Chr7 | 5238122 | 5238268 | chr9 | 45122096 | 45122241 | Inter-translocation |
| Chr7 | 7514789 | 7516443 | chr8 | 19604759 | 19606411 | Inter-translocation |
| Chr7 | 7927240 | 7927421 | chr5 | 23520539 | 23520718 | Inter-translocation |
| Chr7 | 8337935 | 8339655 | chr8 | 13169067 | 13170788 | Inter-translocation |
| Chr7 | 8347275 | 8350234 | chr5 | 24581216 | 24584170 | Inter-translocation |
| Chr7 | 8352659 | 8350871 | chr3 | 9770452 | 9772244 | Inversion |
| Chr7 | 8623043 | 8616724 | chr3 | 30973144 | 30979480 | Inversion |
| Chr7 | 10998604 | 10999011 | chr8 | 4387384 | 4387791 | Inter-translocation |
| Chr7 | 11003292 | 11001808 | chr8 | 4466052 | 4467535 | Inversion |
| Chr7 | 11449256 | 11449084 | chr8 | 29452763 | 29452932 | Inversion |
| Chr7 | 11450101 | 11449330 | chr7 | 11511807 | 11512579 | Inversion |
| Chr7 | 11473499 | 11473880 | chr7 | 11585283 | 11585672 | Intra-translocation |
| Chr7 | 11473500 | 11473430 | chr9 | 22184293 | 22184363 | Inversion |
| Chr7 | 12061352 | 12062281 | chr9 | 30288119 | 30289048 | Inter-translocation |
| Chr7 | 12483755 | 12484197 | chr7 | 12665594 | 12666037 | Intra-translocation |
| Chr7 | 12728107 | 12727984 | chr8 | 25200185 | 25200308 | Inversion |
| Chr7 | 14760901 | 14761682 | chr1 | 32122648 | 32123434 | Inter-translocation |
| Chr7 | 14768962 | 14768163 | chr3 | 43098497 | 43099296 | Inversion |
| Chr7 | 14791572 | 14792624 | chr6 | 34652311 | 34653399 | Inter-translocation |
| Chr7 | 15147616 | 15146576 | chr9 | 39666529 | 39667569 | Inversion |
| Chr7 | 15817792 | 15834025 | chr7 | 15975378 | 15991611 | Intra-translocation |
| Chr7 | 16776207 | 16772041 | chr4 | 14108089 | 14112272 | Inversion |
| Chr7 | 16965770 | 16965688 | chr7 | 32742999 | 32743081 | Inversion |
| Chr7 | 17035669 | 17026422 | chr1 | 30879037 | 30888282 | Inversion |
| Chr7 | 17122787 | 17120929 | chr8 | 10952578 | 10954436 | Inversion |
| Chr7 | 17128589 | 17126308 | chr7 | 33990754 | 33993032 | Inversion |
| Chr7 | 17680654 | 17680343 | chr4 | 5661007 | 5661328 | Inversion |
| Chr7 | 17822759 | 17822947 | chr1 | 21898770 | 21898956 | Inter-translocation |
| Chr7 | 19647645 | 19647842 | chr4 | 34267321 | 34267518 | Inter-translocation |
| Chr7 | 19705612 | 19704135 | chr7 | 22712559 | 22714042 | Inversion |
| Chr7 | 20398068 | 20396573 | chr8 | 4728386 | 4729898 | Inversion |
| Chr7 | 20848344 | 20848130 | chr8 | 7389567 | 7389781 | Inversion |
| Chr7 | 22027826 | 22027944 | chr8 | 3311339 | 3311457 | Inter-translocation |
| Chr7 | 22147222 | 22147404 | chr6 | 1007625 | 1007807 | Inter-translocation |
| Chr7 | 22147665 | 22147416 | chr3 | 23503 | 23753 | Inversion |
| Chr7 | 22208318 | 22208433 | chr9 | 58901396 | 58901511 | Inter-translocation |
| Chr7 | 22239029 | 22239313 | chr7 | 35013811 | 35014095 | Intra-translocation |
| Chr7 | 22638359 | 22639181 | chr7 | 22725776 | 22726599 | Intra-translocation |
| Chr7 | 22652118 | 22653019 | chr6 | 32775286 | 32776225 | Inter-translocation |
| Chr7 | 22843934 | 22843598 | chr3 | 22403079 | 22403417 | Inversion |
| Chr7 | 22948074 | 22945693 | chr8 | 22067992 | 22070388 | Inversion |
| Chr7 | 22948765 | 22948281 | chr8 | 22067454 | 22067947 | Inversion |
| Chr7 | 22963501 | 22962243 | chr8 | 4265556 | 4266835 | Inversion |
| Chr7 | 23149385 | 23149206 | chr8 | 40159757 | 40159936 | Inversion |
| Chr7 | 23194327 | 23194606 | chr3 | 12931710 | 12931991 | Inter-translocation |
| Chr7 | 23245242 | 23245044 | chr9 | 18364630 | 18364828 | Inversion |
| Chr7 | 23724349 | 23724525 | chr1 | 14628600 | 14628776 | Inter-translocation |
| Chr7 | 23732266 | 23732061 | chr6 | 32231949 | 32232154 | Inversion |
| Chr7 | 24037094 | 24041863 | chr8 | 24091912 | 24096669 | Inter-translocation |
| Chr7 | 25108584 | 25108866 | chr3 | 3065958 | 3066240 | Inter-translocation |
| Chr7 | 25534623 | 25536892 | chr7 | 22747633 | 22749908 | Intra-translocation |
| Chr7 | 26066977 | 26067252 | chr1 | 24640903 | 24641178 | Inter-translocation |
| Chr7 | 26203478 | 26203639 | chr4 | 32739092 | 32739253 | Inter-translocation |
| Chr7 | 26212759 | 26212602 | chr6 | 32231615 | 32231772 | Inversion |
| Chr7 | 26268769 | 26270151 | chr3 | 7135035 | 7136421 | Inter-translocation |
| Chr7 | 26426535 | 26312651 | chr2 | 22746954 | 22860821 | Inversion |
| Chr7 | 26457199 | 26426571 | chr2 | 22716030 | 22746658 | Inversion |
| Chr7 | 26461824 | 26457196 | chr2 | 22711057 | 22715685 | Inversion |
| Chr7 | 26471257 | 26462446 | chr2 | 22700302 | 22709134 | Inversion |
| Chr7 | 26471497 | 26471255 | chr2 | 22699785 | 22700027 | Inversion |
| Chr7 | 26475873 | 26471493 | chr2 | 22695131 | 22699512 | Inversion |
| Chr7 | 26476653 | 26475923 | chr2 | 22694082 | 22694812 | Inversion |
| Chr7 | 26478968 | 26476649 | chr2 | 22691689 | 22694008 | Inversion |
| Chr7 | 26487304 | 26478968 | chr2 | 22682405 | 22690754 | Inversion |
| Chr7 | 26488347 | 26487300 | chr2 | 22669791 | 22670833 | Inversion |
| Chr7 | 26491706 | 26488511 | chr2 | 22666537 | 22669795 | Inversion |
| Chr7 | 27140107 | 27138376 | chr7 | 27005026 | 27006773 | Inversion |
| Chr7 | 27145932 | 27140980 | chr7 | 27000166 | 27005028 | Inversion |
| Chr7 | 27159462 | 27158173 | chr8 | 10476592 | 10477879 | Inversion |
| Chr7 | 27412094 | 27411738 | chr1 | 633804 | 634160 | Inversion |
| Chr7 | 27542005 | 27541726 | chr2 | 38344430 | 38344709 | Inversion |
| Chr7 | 27624694 | 27624420 | chr2 | 45697061 | 45697335 | Inversion |
| Chr7 | 27959068 | 27958706 | chr6 | 34013371 | 34013733 | Inversion |
| Chr7 | 28532345 | 28532001 | chr3 | 46730115 | 46730459 | Inversion |
| Chr7 | 28610043 | 28609691 | chr3 | 24518902 | 24519254 | Inversion |
| Chr7 | 28954255 | 28974455 | chr7 | 28846470 | 28866747 | Intra-translocation |
| Chr7 | 29663248 | 29663600 | chr4 | 12841297 | 12841649 | Inter-translocation |
| Chr7 | 29974801 | 29974420 | chr7 | 31019453 | 31019832 | Inversion |
| Chr7 | 30056340 | 30060967 | chr7 | 29966067 | 29970750 | Intra-translocation |
| Chr7 | 30072833 | 30073410 | chr7 | 29985251 | 29985820 | Intra-translocation |
| Chr7 | 30323697 | 30323972 | chr4 | 8848852 | 8849127 | Inter-translocation |
| Chr7 | 30361669 | 30362631 | chr3 | 11732354 | 11733305 | Inter-translocation |
| Chr7 | 30365988 | 30366285 | chr3 | 11736342 | 11736639 | Inter-translocation |
| Chr7 | 30396191 | 30395581 | chr7 | 30374640 | 30375250 | Inversion |
| Chr7 | 30421928 | 30422257 | chr7 | 30324043 | 30324370 | Intra-translocation |
| Chr7 | 30521381 | 30521726 | chr9 | 42692562 | 42692908 | Inter-translocation |
| Chr7 | 30560400 | 30560155 | chr8 | 3159075 | 3159319 | Inversion |
| Chr7 | 30585772 | 30585994 | chr8 | 25737637 | 25737859 | Inter-translocation |
| Chr7 | 30604581 | 30591197 | chr6 | 3106802 | 3120163 | Inversion |
| Chr7 | 30651159 | 30652465 | chr5 | 16268392 | 16269775 | Inter-translocation |
| Chr7 | 30652491 | 30652795 | chr5 | 16269923 | 16270220 | Inter-translocation |
| Chr7 | 30675590 | 30677137 | chr7 | 30554904 | 30556460 | Intra-translocation |
| Chr7 | 30866570 | 30866320 | chr7 | 30744142 | 30744393 | Inversion |
| Chr7 | 30870602 | 30870849 | chr3 | 8151655 | 8151903 | Inter-translocation |
| Chr7 | 30872399 | 30872588 | chr2 | 41272140 | 41272329 | Inter-translocation |
| Chr7 | 31132432 | 31132611 | chr9 | 21335300 | 21335479 | Inter-translocation |
| Chr7 | 31377481 | 31377826 | chr4 | 37031359 | 37031704 | Inter-translocation |
| Chr7 | 31396498 | 31399621 | chr2 | 42460059 | 42463172 | Inter-translocation |
| Chr7 | 31438418 | 31438143 | chr9 | 4461015 | 4461290 | Inversion |
| Chr7 | 31491883 | 31492159 | chr2 | 3683992 | 3684268 | Inter-translocation |
| Chr7 | 31602385 | 31613990 | chr2 | 32786113 | 32797724 | Inter-translocation |
| Chr7 | 31623662 | 31620182 | chr2 | 41447224 | 41450718 | Inversion |
| Chr7 | 31640811 | 31640456 | chr9 | 48663519 | 48663874 | Inversion |
| Chr7 | 31648521 | 31648244 | chr2 | 46374872 | 46375149 | Inversion |
| Chr7 | 31907307 | 31907939 | chr3 | 13941973 | 13942606 | Inter-translocation |
| Chr7 | 31909791 | 31910143 | chr9 | 41617101 | 41617453 | Inter-translocation |
| Chr7 | 31937659 | 31937306 | chr5 | 37444165 | 37444518 | Inversion |
| Chr7 | 31937663 | 31937314 | chr7 | 22561921 | 22562270 | Inversion |
| Chr7 | 32147455 | 32142614 | chr8 | 5853588 | 5858440 | Inversion |
| Chr7 | 32156544 | 32150487 | chr7 | 32025720 | 32031768 | Inversion |
| Chr7 | 32673928 | 32673667 | chr7 | 27155447 | 27155710 | Inversion |
| Chr7 | 32720559 | 32720206 | chr6 | 34338148 | 34338501 | Inversion |
| Chr7 | 33306865 | 33306379 | chr8 | 5904032 | 5904521 | Inversion |
| Chr7 | 33324890 | 33320574 | chr7 | 33360074 | 33364376 | Inversion |
| Chr7 | 33355097 | 33354740 | chr8 | 29434068 | 29434425 | Inversion |
| Chr7 | 33364349 | 33365832 | chr7 | 33190118 | 33191597 | Intra-translocation |
| Chr7 | 33366364 | 33366527 | chr7 | 33192130 | 33192293 | Intra-translocation |
| Chr7 | 33367452 | 33367605 | chr7 | 33188374 | 33188528 | Intra-translocation |
| Chr7 | 33400274 | 33395476 | chr8 | 40233083 | 40237881 | Inversion |
| Chr7 | 33439505 | 33438728 | chr9 | 34405399 | 34406175 | Inversion |
| Chr7 | 33456700 | 33457889 | chr3 | 12895828 | 12897015 | Inter-translocation |
| Chr7 | 33469762 | 33468899 | chr7 | 33183593 | 33184461 | Inversion |
| Chr7 | 33475295 | 33475695 | chr7 | 33197354 | 33197774 | Intra-translocation |
| Chr7 | 33493037 | 33492887 | chr7 | 33189458 | 33189608 | Inversion |
| Chr7 | 33499515 | 33499713 | chr9 | 38552226 | 38552437 | Inter-translocation |
| Chr7 | 33508570 | 33508413 | chr9 | 42685415 | 42685563 | Inversion |
| Chr7 | 33547159 | 33547343 | chr7 | 33189967 | 33190154 | Intra-translocation |
| Chr7 | 33550949 | 33548223 | chr7 | 33359756 | 33362412 | Inversion |
| Chr7 | 33566499 | 33561560 | chr9 | 41729418 | 41734290 | Inversion |
| Chr7 | 33575007 | 33573535 | chr7 | 33388190 | 33389708 | Inversion |
| Chr7 | 33657293 | 33646526 | chr7 | 33348285 | 33359157 | Inversion |
| Chr7 | 33658911 | 33657282 | chr7 | 33346289 | 33347940 | Inversion |
| Chr7 | 33667289 | 33659603 | chr7 | 33338504 | 33346293 | Inversion |
| Chr7 | 33681153 | 33668777 | chr7 | 33326127 | 33338505 | Inversion |
| Chr7 | 33683110 | 33682252 | chr7 | 33325251 | 33326106 | Inversion |
| Chr7 | 33684637 | 33683125 | chr7 | 33323346 | 33324912 | Inversion |
| Chr7 | 33685511 | 33684638 | chr7 | 33322298 | 33323206 | Inversion |
| Chr7 | 33699054 | 33695540 | chr7 | 33379261 | 33382758 | Inversion |
| Chr7 | 33720131 | 33718347 | chr7 | 33320487 | 33322267 | Inversion |
| Chr7 | 33724315 | 33720129 | chr7 | 33316171 | 33320367 | Inversion |
| Chr7 | 33731357 | 33724849 | chr7 | 33308469 | 33314977 | Inversion |
| Chr7 | 33734586 | 33731357 | chr7 | 33305101 | 33308329 | Inversion |
| Chr7 | 33740324 | 33734584 | chr7 | 33299338 | 33305060 | Inversion |
| Chr7 | 33759510 | 33740362 | chr7 | 33279637 | 33298642 | Inversion |
| Chr7 | 33760986 | 33759854 | chr7 | 33278506 | 33279641 | Inversion |
| Chr7 | 33776593 | 33761143 | chr7 | 33263131 | 33278506 | Inversion |
| Chr7 | 33778720 | 33778636 | chr7 | 33261787 | 33261871 | Inversion |
| Chr7 | 33778877 | 33778722 | chr7 | 33261307 | 33261452 | Inversion |
| Chr7 | 33795803 | 33779120 | chr7 | 33244578 | 33261310 | Inversion |
| Chr7 | 33802816 | 33795921 | chr7 | 33237676 | 33244576 | Inversion |
| Chr7 | 33803005 | 33802813 | chr7 | 33237211 | 33237403 | Inversion |
| Chr7 | 33804383 | 33803002 | chr7 | 33235566 | 33236863 | Inversion |
| Chr7 | 33813460 | 33804383 | chr7 | 33226454 | 33235462 | Inversion |
| Chr7 | 33814796 | 33813846 | chr7 | 33225463 | 33226422 | Inversion |
| Chr7 | 33815020 | 33814794 | chr7 | 33225175 | 33225402 | Inversion |
| Chr7 | 33815169 | 33815014 | chr1 | 25289391 | 25289546 | Inversion |
| Chr7 | 33816228 | 33815166 | chr7 | 33224103 | 33225178 | Inversion |
| Chr7 | 33817161 | 33816366 | chr7 | 33223288 | 33224080 | Inversion |
| Chr7 | 33829759 | 33817421 | chr7 | 33210929 | 33223296 | Inversion |
| Chr7 | 33832892 | 33829766 | chr7 | 33207686 | 33210788 | Inversion |
| Chr7 | 33840637 | 33832904 | chr7 | 33199797 | 33207536 | Inversion |
| Chr7 | 33848058 | 33847161 | chr7 | 33182373 | 33183270 | Inversion |
| Chr7 | 33862664 | 33857762 | chr7 | 33466964 | 33471878 | Inversion |
| Chr7 | 33864665 | 33863003 | chr7 | 33465294 | 33466966 | Inversion |
| Chr7 | 33867634 | 33868332 | chr8 | 3869522 | 3870221 | Inter-translocation |
| Chr7 | 33874259 | 33868333 | chr7 | 33459405 | 33465302 | Inversion |
| Chr7 | 33886866 | 33874256 | chr7 | 33446209 | 33458810 | Inversion |
| Chr7 | 33899057 | 33886860 | chr7 | 33433701 | 33445850 | Inversion |
| Chr7 | 33917054 | 33899058 | chr7 | 33415576 | 33433613 | Inversion |
| Chr7 | 33920750 | 33917052 | chr7 | 33411522 | 33415223 | Inversion |
| Chr7 | 33921227 | 33920747 | chr7 | 33410705 | 33411181 | Inversion |
| Chr7 | 33925817 | 33921215 | chr7 | 33405757 | 33410387 | Inversion |
| Chr7 | 33935432 | 33925852 | chr7 | 33395520 | 33405128 | Inversion |
| Chr7 | 33962112 | 33961997 | chr4 | 33094056 | 33094171 | Inversion |
| Chr7 | 35471526 | 35471921 | chr4 | 33354916 | 33355312 | Inter-translocation |
| Chr7 | 35949267 | 35948916 | chr9 | 19994654 | 19995005 | Inversion |
| Chr7 | 36007202 | 36006953 | chr7 | 30782666 | 30782912 | Inversion |
| Chr7 | 36101409 | 36101214 | chr6 | 30551154 | 30551350 | Inversion |
| Chr7 | 36153159 | 36153432 | chr3 | 14730580 | 14730853 | Inter-translocation |
| Chr7 | 36500846 | 36512528 | chr7 | 30352742 | 30364384 | Intra-translocation |
| Chr7 | 36512528 | 36519275 | chr7 | 30365610 | 30372393 | Intra-translocation |
| Chr7 | 36536218 | 36536640 | chr4 | 12148872 | 12149297 | Inter-translocation |
| Chr8 | 321434 | 320382 | chr8 | 29114360 | 29115414 | Inversion |
| Chr8 | 615301 | 616632 | chr4 | 5672506 | 5673835 | Inter-translocation |
| Chr8 | 1379090 | 1378967 | chr4 | 984847 | 984971 | Inversion |
| Chr8 | 1579841 | 1579488 | chr3 | 46134658 | 46135011 | Inversion |
| Chr8 | 2044751 | 2038138 | chr9 | 36825219 | 36831832 | Inversion |
| Chr8 | 2314228 | 2314038 | chr8 | 2300888 | 2301079 | Inversion |
| Chr8 | 2486284 | 2486533 | chr8 | 38818729 | 38818977 | Intra-translocation |
| Chr8 | 2527390 | 2527116 | chr1 | 25228652 | 25228926 | Inversion |
| Chr8 | 2626347 | 2619221 | chr9 | 28584078 | 28591185 | Inversion |
| Chr8 | 2650058 | 2650284 | chr8 | 11674202 | 11674431 | Intra-translocation |
| Chr8 | 2655407 | 2651365 | chr3 | 46271350 | 46275391 | Inversion |
| Chr8 | 2758109 | 2771437 | chr8 | 20679150 | 20692478 | Intra-translocation |
| Chr8 | 2904968 | 2904612 | chr5 | 7949546 | 7949903 | Inversion |
| Chr8 | 2915037 | 2914452 | chr3 | 47289603 | 47290198 | Inversion |
| Chr8 | 2925976 | 2926331 | chr8 | 8407314 | 8407669 | Intra-translocation |
| Chr8 | 2929070 | 2930307 | chr8 | 3237614 | 3238835 | Intra-translocation |
| Chr8 | 2958897 | 2959027 | chr1 | 14701209 | 14701339 | Inter-translocation |
| Chr8 | 3084547 | 3084171 | chr1 | 10667962 | 10668336 | Inversion |
| Chr8 | 3194353 | 3194677 | chr8 | 3588234 | 3588558 | Intra-translocation |
| Chr8 | 3194806 | 3198841 | chr8 | 3588673 | 3592671 | Intra-translocation |
| Chr8 | 3437349 | 3436968 | chr2 | 42379766 | 42380146 | Inversion |
| Chr8 | 3481557 | 3481984 | chr6 | 3203544 | 3203970 | Inter-translocation |
| Chr8 | 3505331 | 3504981 | chr7 | 22630424 | 22630774 | Inversion |
| Chr8 | 3508574 | 3508378 | chr8 | 6929110 | 6929306 | Inversion |
| Chr8 | 3515896 | 3515807 | chr8 | 2653938 | 2654027 | Inversion |
| Chr8 | 3546327 | 3545980 | chr9 | 10749603 | 10749950 | Inversion |
| Chr8 | 3592372 | 3592512 | chr1 | 14698148 | 14698288 | Inter-translocation |
| Chr8 | 3641326 | 3642323 | chr8 | 11370951 | 11371947 | Intra-translocation |
| Chr8 | 3658882 | 3654510 | chr8 | 11062123 | 11066496 | Inversion |
| Chr8 | 3679901 | 3680074 | chr8 | 10591062 | 10591235 | Intra-translocation |
| Chr8 | 3701619 | 3698232 | chr4 | 10128323 | 10131771 | Inversion |
| Chr8 | 3709607 | 3708882 | chr8 | 3963080 | 3963825 | Inversion |
| Chr8 | 3748007 | 3742354 | chr8 | 30472958 | 30478566 | Inversion |
| Chr8 | 3750175 | 3753198 | chr2 | 7972482 | 7975503 | Inter-translocation |
| Chr8 | 3761706 | 3759126 | chr3 | 22454818 | 22457396 | Inversion |
| Chr8 | 3774632 | 3773821 | chr8 | 4239827 | 4240607 | Inversion |
| Chr8 | 3780084 | 3781103 | chr8 | 4028517 | 4029537 | Intra-translocation |
| Chr8 | 3808959 | 3808615 | chr8 | 21580790 | 21581134 | Inversion |
| Chr8 | 3815727 | 3810530 | chr8 | 25140481 | 25145649 | Inversion |
| Chr8 | 3822081 | 3823761 | chr8 | 4846375 | 4848050 | Intra-translocation |
| Chr8 | 3823763 | 3822105 | chr2 | 3055748 | 3057423 | Inversion |
| Chr8 | 3832689 | 3832336 | chr4 | 10018156 | 10018510 | Inversion |
| Chr8 | 3834125 | 3837985 | chr1 | 13175229 | 13179092 | Inter-translocation |
| Chr8 | 3840698 | 3840428 | chr9 | 36754952 | 36755221 | Inversion |
| Chr8 | 4062663 | 4064946 | chr8 | 4346654 | 4348922 | Intra-translocation |
| Chr8 | 4116659 | 4116276 | chr8 | 4482194 | 4482575 | Inversion |
| Chr8 | 4162645 | 4166967 | chr8 | 30009623 | 30013942 | Intra-translocation |
| Chr8 | 4173129 | 4174618 | chr9 | 41808684 | 41810175 | Inter-translocation |
| Chr8 | 4263368 | 4263263 | chr3 | 14649204 | 14649309 | Inversion |
| Chr8 | 4300177 | 4300024 | chr3 | 42746298 | 42746451 | Inversion |
| Chr8 | 4312452 | 4311307 | chr8 | 8530353 | 8531520 | Inversion |
| Chr8 | 4314984 | 4313617 | chr8 | 25824051 | 25825417 | Inversion |
| Chr8 | 4382757 | 4385798 | chr8 | 4639605 | 4642650 | Intra-translocation |
| Chr8 | 4418631 | 4419558 | chr8 | 4621628 | 4622578 | Intra-translocation |
| Chr8 | 4577111 | 4576838 | chr6 | 3262129 | 3262402 | Inversion |
| Chr8 | 4682515 | 4681768 | chr8 | 4448749 | 4449484 | Inversion |
| Chr8 | 4684481 | 4687514 | chr8 | 4922941 | 4925991 | Intra-translocation |
| Chr8 | 4687756 | 4687982 | chr8 | 37779802 | 37780030 | Intra-translocation |
| Chr8 | 4694483 | 4695499 | chr8 | 4908385 | 4909406 | Intra-translocation |
| Chr8 | 4695495 | 4696021 | chr8 | 4915746 | 4916273 | Intra-translocation |
| Chr8 | 4697519 | 4698307 | chr8 | 4918605 | 4919384 | Intra-translocation |
| Chr8 | 4812005 | 4811033 | chr5 | 16434940 | 16435910 | Inversion |
| Chr8 | 4813948 | 4812013 | chr5 | 16429819 | 16431771 | Inversion |
| Chr8 | 4924269 | 4924623 | chr8 | 5152235 | 5152590 | Intra-translocation |
| Chr8 | 4924624 | 4940241 | chr8 | 5152861 | 5168440 | Intra-translocation |
| Chr8 | 5087683 | 5087899 | chr8 | 2848798 | 2849013 | Intra-translocation |
| Chr8 | 5102935 | 5105080 | chr8 | 39229312 | 39231451 | Intra-translocation |
| Chr8 | 5107745 | 5107168 | chr8 | 5423106 | 5423678 | Inversion |
| Chr8 | 5247132 | 5247506 | chr8 | 7586517 | 7586881 | Intra-translocation |
| Chr8 | 5250011 | 5249521 | chr8 | 20201569 | 20202070 | Inversion |
| Chr8 | 5390623 | 5390425 | chr9 | 17910140 | 17910345 | Inversion |
| Chr8 | 5443338 | 5446353 | chr8 | 5800543 | 5803559 | Intra-translocation |
| Chr8 | 5458718 | 5465020 | chr8 | 5794281 | 5800545 | Intra-translocation |
| Chr8 | 5493635 | 5491444 | chr5 | 15138437 | 15140628 | Inversion |
| Chr8 | 5536979 | 5530688 | chr9 | 42110908 | 42117182 | Inversion |
| Chr8 | 5686285 | 5686637 | chr7 | 22334048 | 22334400 | Inter-translocation |
| Chr8 | 5712032 | 5711463 | chr8 | 12627853 | 12628446 | Inversion |
| Chr8 | 5980792 | 5980332 | chr8 | 13008669 | 13009126 | Inversion |
| Chr8 | 6010407 | 6006707 | chr8 | 6051538 | 6055231 | Inversion |
| Chr8 | 6012160 | 6010624 | chr8 | 6049795 | 6051321 | Inversion |
| Chr8 | 6089076 | 6087363 | chr8 | 13223155 | 13224854 | Inversion |
| Chr8 | 6247185 | 6242072 | chr8 | 6546565 | 6551678 | Inversion |
| Chr8 | 6326336 | 6325377 | chr9 | 30033753 | 30034712 | Inversion |
| Chr8 | 6341164 | 6341779 | chr5 | 9824091 | 9824704 | Inter-translocation |
| Chr8 | 6353653 | 6350854 | chr3 | 42292037 | 42294832 | Inversion |
| Chr8 | 6619881 | 6613332 | chr8 | 18298643 | 18305187 | Inversion |
| Chr8 | 6623932 | 6625104 | chr8 | 17789202 | 17790395 | Intra-translocation |
| Chr8 | 6688827 | 6687791 | chr7 | 27041902 | 27042921 | Inversion |
| Chr8 | 6692802 | 6688741 | chr4 | 10428824 | 10432893 | Inversion |
| Chr8 | 6694496 | 6688945 | chr7 | 27036254 | 27041805 | Inversion |
| Chr8 | 6714261 | 6714503 | chr8 | 25328942 | 25329184 | Intra-translocation |
| Chr8 | 6729088 | 6731798 | chr8 | 29795512 | 29798259 | Intra-translocation |
| Chr8 | 6813814 | 6808510 | chr8 | 29442219 | 29447524 | Inversion |
| Chr8 | 6839224 | 6840381 | chr8 | 7203936 | 7205104 | Intra-translocation |
| Chr8 | 6886992 | 6893079 | chr8 | 7207930 | 7214007 | Intra-translocation |
| Chr8 | 6923860 | 6927404 | chr8 | 7172808 | 7176333 | Intra-translocation |
| Chr8 | 6942785 | 6943137 | chr3 | 50524916 | 50525268 | Inter-translocation |
| Chr8 | 6965465 | 6965110 | chr8 | 2499926 | 2500280 | Inversion |
| Chr8 | 6965784 | 6965974 | chr9 | 9896623 | 9896814 | Inter-translocation |
| Chr8 | 7185676 | 7186733 | chr8 | 7445239 | 7446298 | Intra-translocation |
| Chr8 | 7190798 | 7191826 | chr8 | 7444267 | 7445237 | Intra-translocation |
| Chr8 | 7237986 | 7239605 | chr8 | 7514437 | 7516037 | Intra-translocation |
| Chr8 | 7250168 | 7250519 | chr7 | 23269707 | 23270058 | Inter-translocation |
| Chr8 | 7254599 | 7253923 | chr1 | 24864527 | 24865203 | Inversion |
| Chr8 | 7265660 | 7264599 | chr9 | 19627560 | 19628621 | Inversion |
| Chr8 | 7282657 | 7285460 | chr1 | 26810659 | 26813446 | Inter-translocation |
| Chr8 | 7318722 | 7316597 | chr8 | 9980424 | 9982570 | Inversion |
| Chr8 | 7321967 | 7321738 | chr8 | 30051463 | 30051694 | Inversion |
| Chr8 | 7477740 | 7477056 | chr9 | 23842504 | 23843176 | Inversion |
| Chr8 | 7545720 | 7546110 | chr8 | 11612807 | 11613201 | Intra-translocation |
| Chr8 | 7567534 | 7567408 | chr8 | 30273436 | 30273562 | Inversion |
| Chr8 | 7590719 | 7594375 | chr8 | 38302958 | 38306606 | Intra-translocation |
| Chr8 | 7651676 | 7651962 | chr8 | 12033805 | 12034089 | Intra-translocation |
| Chr8 | 7729029 | 7729276 | chr8 | 13393590 | 13393837 | Intra-translocation |
| Chr8 | 7765510 | 7764105 | chr8 | 12577240 | 12578645 | Inversion |
| Chr8 | 7779757 | 7779226 | chr6 | 35832532 | 35833063 | Inversion |
| Chr8 | 7907095 | 7906748 | chr9 | 18749439 | 18749786 | Inversion |
| Chr8 | 7954745 | 7954028 | chr3 | 46163286 | 46163997 | Inversion |
| Chr8 | 7955268 | 7954762 | chr3 | 46162632 | 46163142 | Inversion |
| Chr8 | 7958107 | 7959821 | chr9 | 27458280 | 27459984 | Inter-translocation |
| Chr8 | 8025515 | 8026786 | chr3 | 47301172 | 47302442 | Inter-translocation |
| Chr8 | 8037639 | 8041290 | chr9 | 30284509 | 30288109 | Inter-translocation |
| Chr8 | 8065481 | 8064650 | chr6 | 32775285 | 32776111 | Inversion |
| Chr8 | 8095417 | 8090712 | chr8 | 18827885 | 18832604 | Inversion |
| Chr8 | 8101258 | 8102374 | chr9 | 31099413 | 31100528 | Inter-translocation |
| Chr8 | 8115988 | 8117125 | chr5 | 9028998 | 9030137 | Inter-translocation |
| Chr8 | 8119716 | 8120050 | chr8 | 20489204 | 20489530 | Intra-translocation |
| Chr8 | 8165715 | 8165493 | chr5 | 24080673 | 24080895 | Inversion |
| Chr8 | 8172163 | 8171978 | chr7 | 4475368 | 4475554 | Inversion |
| Chr8 | 8214186 | 8211758 | chr8 | 26114363 | 26116793 | Inversion |
| Chr8 | 8242993 | 8242186 | chr7 | 32100307 | 32101114 | Inversion |
| Chr8 | 8396518 | 8396403 | chr6 | 34408935 | 34409050 | Inversion |
| Chr8 | 8544139 | 8544437 | chr8 | 4065648 | 4065945 | Intra-translocation |
| Chr8 | 8553958 | 8549461 | chr8 | 8694258 | 8698730 | Inversion |
| Chr8 | 8591641 | 8591304 | chr3 | 44796082 | 44796419 | Inversion |
| Chr8 | 8605239 | 8604841 | chr3 | 8535028 | 8535425 | Inversion |
| Chr8 | 8689375 | 8689040 | chr7 | 30990043 | 30990378 | Inversion |
| Chr8 | 8741933 | 8747930 | chr4 | 927500 | 933534 | Inter-translocation |
| Chr8 | 8747993 | 8748427 | chr3 | 13397650 | 13398086 | Inter-translocation |
| Chr8 | 8748297 | 8752092 | chr4 | 933802 | 937602 | Inter-translocation |
| Chr8 | 8755225 | 8752755 | chr8 | 8271463 | 8273938 | Inversion |
| Chr8 | 8803516 | 8802013 | chr6 | 28283836 | 28285336 | Inversion |
| Chr8 | 8832940 | 8831246 | chr8 | 11093372 | 11095111 | Inversion |
| Chr8 | 8851565 | 8852322 | chr8 | 7186439 | 7187180 | Intra-translocation |
| Chr8 | 8927569 | 8926638 | chr3 | 46831087 | 46832015 | Inversion |
| Chr8 | 8935999 | 8930766 | chr8 | 29619160 | 29624397 | Inversion |
| Chr8 | 8950785 | 8949806 | chr6 | 20761315 | 20762295 | Inversion |
| Chr8 | 9051978 | 9054377 | chr8 | 2592040 | 2594417 | Intra-translocation |
| Chr8 | 9209422 | 9212327 | chr3 | 46524679 | 46527580 | Inter-translocation |
| Chr8 | 9257482 | 9255187 | chr6 | 17302567 | 17304862 | Inversion |
| Chr8 | 9274419 | 9269696 | chr8 | 18827885 | 18832604 | Inversion |
| Chr8 | 9447086 | 9446020 | chr9 | 31006631 | 31007698 | Inversion |
| Chr8 | 9451591 | 9452897 | chr9 | 34260717 | 34262023 | Inter-translocation |
| Chr8 | 9583511 | 9583136 | chr8 | 9607979 | 9608354 | Inversion |
| Chr8 | 9614123 | 9613797 | chr1 | 25268699 | 25269018 | Inversion |
| Chr8 | 9621434 | 9624603 | chr4 | 5560517 | 5563692 | Inter-translocation |
| Chr8 | 9635309 | 9636735 | chr8 | 22732265 | 22733693 | Intra-translocation |
| Chr8 | 9637391 | 9638578 | chr1 | 25269119 | 25270307 | Inter-translocation |
| Chr8 | 9903808 | 9904710 | chr6 | 22262358 | 22263259 | Inter-translocation |
| Chr8 | 9939888 | 9940377 | chr8 | 10130187 | 10130676 | Intra-translocation |
| Chr8 | 10074389 | 10073076 | chr8 | 10279816 | 10281146 | Inversion |
| Chr8 | 10077901 | 10074385 | chr8 | 10274859 | 10278415 | Inversion |
| Chr8 | 10080472 | 10078605 | chr2 | 41901548 | 41903414 | Inversion |
| Chr8 | 10084811 | 10081474 | chr8 | 10265035 | 10268373 | Inversion |
| Chr8 | 10099745 | 10085174 | chr8 | 10250510 | 10265037 | Inversion |
| Chr8 | 10126348 | 10127259 | chr8 | 10402497 | 10403406 | Intra-translocation |
| Chr8 | 10138456 | 10138310 | chr2 | 37484040 | 37484188 | Inversion |
| Chr8 | 10140100 | 10139792 | chr8 | 30096794 | 30097101 | Inversion |
| Chr8 | 10169639 | 10172895 | chr8 | 10346646 | 10349909 | Intra-translocation |
| Chr8 | 10182811 | 10182272 | chr8 | 2676782 | 2677356 | Inversion |
| Chr8 | 10199422 | 10199151 | chr8 | 10476226 | 10476497 | Inversion |
| Chr8 | 10200278 | 10199379 | chr8 | 10482907 | 10483806 | Inversion |
| Chr8 | 10279626 | 10278568 | chr5 | 15811597 | 15812643 | Inversion |
| Chr8 | 10308598 | 10311316 | chr8 | 10759745 | 10762429 | Intra-translocation |
| Chr8 | 10384937 | 10384706 | chr9 | 31348661 | 31348892 | Inversion |
| Chr8 | 10457995 | 10459047 | chr8 | 10917965 | 10919033 | Intra-translocation |
| Chr8 | 10459970 | 10460490 | chr8 | 10919365 | 10919899 | Intra-translocation |
| Chr8 | 10513659 | 10510848 | chr5 | 14792138 | 14794954 | Inversion |
| Chr8 | 10518590 | 10519424 | chr8 | 10886886 | 10887727 | Intra-translocation |
| Chr8 | 10553914 | 10550640 | chr8 | 15815687 | 15818951 | Inversion |
| Chr8 | 10561496 | 10560103 | chr9 | 33721278 | 33722671 | Inversion |
| Chr8 | 10571982 | 10572887 | chr8 | 20198594 | 20199488 | Intra-translocation |
| Chr8 | 10574516 | 10574906 | chr8 | 20200570 | 20200964 | Intra-translocation |
| Chr8 | 10629910 | 10630329 | chr1 | 9057768 | 9058187 | Inter-translocation |
| Chr8 | 10742155 | 10742328 | chr8 | 4533554 | 4533727 | Intra-translocation |
| Chr8 | 10752546 | 10752671 | chr8 | 37878431 | 37878556 | Intra-translocation |
| Chr8 | 10793131 | 10797008 | chr4 | 35226581 | 35230473 | Inter-translocation |
| Chr8 | 10803127 | 10803463 | chr8 | 25710079 | 25710414 | Intra-translocation |
| Chr8 | 10883773 | 10884816 | chr4 | 6985704 | 6986762 | Inter-translocation |
| Chr8 | 11056746 | 11056162 | chr8 | 37756904 | 37757491 | Inversion |
| Chr8 | 11057101 | 11065336 | chr8 | 37760693 | 37768940 | Intra-translocation |
| Chr8 | 11091428 | 11090297 | chr8 | 40348306 | 40349442 | Inversion |
| Chr8 | 11125676 | 11126161 | chr8 | 10026766 | 10027253 | Intra-translocation |
| Chr8 | 11147045 | 11145752 | chr8 | 4166658 | 4167953 | Inversion |
| Chr8 | 11165503 | 11160988 | chr8 | 11495216 | 11499728 | Inversion |
| Chr8 | 11165855 | 11165506 | chr9 | 32238379 | 32238728 | Inversion |
| Chr8 | 11166315 | 11165855 | chr8 | 11494753 | 11495219 | Inversion |
| Chr8 | 11169887 | 11173213 | chr8 | 27820892 | 27824218 | Intra-translocation |
| Chr8 | 11177999 | 11175588 | chr8 | 11492352 | 11494761 | Inversion |
| Chr8 | 11179511 | 11179886 | chr8 | 12106014 | 12106388 | Intra-translocation |
| Chr8 | 11181249 | 11180448 | chr8 | 11491240 | 11492086 | Inversion |
| Chr8 | 11200563 | 11194456 | chr8 | 11485188 | 11491244 | Inversion |
| Chr8 | 11206154 | 11205830 | chr8 | 11479654 | 11479977 | Inversion |
| Chr8 | 11308143 | 11308529 | chr2 | 45912275 | 45912661 | Inter-translocation |
| Chr8 | 11443065 | 11442769 | chr4 | 10227257 | 10227554 | Inversion |
| Chr8 | 11445019 | 11450584 | chr9 | 33097559 | 33103137 | Inter-translocation |
| Chr8 | 11455495 | 11457918 | chr9 | 17539117 | 17541538 | Inter-translocation |
| Chr8 | 11573320 | 11574637 | chr6 | 1544485 | 1545802 | Inter-translocation |
| Chr8 | 11779463 | 11772553 | chr4 | 10190548 | 10197497 | Inversion |
| Chr8 | 11848743 | 11849091 | chr7 | 33489954 | 33490303 | Inter-translocation |
| Chr8 | 11850729 | 11851070 | chr2 | 41400971 | 41401315 | Inter-translocation |
| Chr8 | 11879073 | 11879416 | chr7 | 25802979 | 25803322 | Inter-translocation |
| Chr8 | 11928643 | 11928278 | chr5 | 15505120 | 15505484 | Inversion |
| Chr8 | 11973735 | 11973506 | chr4 | 35231286 | 35231515 | Inversion |
| Chr8 | 11980265 | 11984677 | chr8 | 9987323 | 9991748 | Intra-translocation |
| Chr8 | 11984677 | 11986456 | chr8 | 12189178 | 12190979 | Intra-translocation |
| Chr8 | 11986512 | 11987803 | chr8 | 12190949 | 12192242 | Intra-translocation |
| Chr8 | 12001096 | 11996080 | chr3 | 49477336 | 49482333 | Inversion |
| Chr8 | 12006655 | 11997788 | chr4 | 12590196 | 12599089 | Inversion |
| Chr8 | 12079927 | 12080040 | chr8 | 5425192 | 5425303 | Intra-translocation |
| Chr8 | 12092677 | 12085302 | chr2 | 18408592 | 18415995 | Inversion |
| Chr8 | 12249749 | 12247052 | chr8 | 22390404 | 22393105 | Inversion |
| Chr8 | 12250821 | 12255073 | chr8 | 22799649 | 22803888 | Intra-translocation |
| Chr8 | 12255075 | 12255362 | chr8 | 25510638 | 25510923 | Intra-translocation |
| Chr8 | 12272600 | 12272911 | chr8 | 12463616 | 12463927 | Intra-translocation |
| Chr8 | 12307818 | 12305895 | chr5 | 14266699 | 14268627 | Inversion |
| Chr8 | 12432481 | 12431851 | chr8 | 12737868 | 12738485 | Inversion |
| Chr8 | 12440006 | 12440609 | chr8 | 37679220 | 37679826 | Intra-translocation |
| Chr8 | 12441400 | 12440084 | chr8 | 27647657 | 27648972 | Inversion |
| Chr8 | 12445263 | 12445520 | chr8 | 29799178 | 29799443 | Intra-translocation |
| Chr8 | 12447197 | 12447003 | chr9 | 40660371 | 40660565 | Inversion |
| Chr8 | 12449240 | 12449619 | chr3 | 24446295 | 24446671 | Inter-translocation |
| Chr8 | 12452018 | 12453996 | chr8 | 12837159 | 12839139 | Intra-translocation |
| Chr8 | 12534275 | 12533925 | chr6 | 989023 | 989373 | Inversion |
| Chr8 | 12536387 | 12545149 | chr8 | 9104748 | 9113517 | Intra-translocation |
| Chr8 | 12584548 | 12587312 | chr1 | 25189160 | 25191948 | Inter-translocation |
| Chr8 | 12619250 | 12618236 | chr9 | 27838882 | 27839909 | Inversion |
| Chr8 | 12631402 | 12622769 | chr8 | 29699329 | 29707984 | Inversion |
| Chr8 | 12631565 | 12623142 | chr4 | 7171303 | 7179733 | Inversion |
| Chr8 | 12857431 | 12857179 | chr2 | 41521959 | 41522213 | Inversion |
| Chr8 | 12857432 | 12861088 | chr8 | 6844657 | 6848283 | Intra-translocation |
| Chr8 | 12964293 | 12962912 | chr3 | 23969033 | 23970424 | Inversion |
| Chr8 | 13010239 | 13010586 | chr6 | 33349196 | 33349543 | Inter-translocation |
| Chr8 | 13123942 | 13119320 | chr8 | 13247989 | 13252622 | Inversion |
| Chr8 | 13127178 | 13124477 | chr8 | 13244515 | 13247233 | Inversion |
| Chr8 | 13127412 | 13127287 | chr8 | 13218942 | 13219068 | Inversion |
| Chr8 | 13131427 | 13131146 | chr8 | 6877768 | 6878047 | Inversion |
| Chr8 | 13167319 | 13167523 | chr4 | 7072881 | 7073085 | Inter-translocation |
| Chr8 | 13167322 | 13167559 | chr9 | 25281972 | 25282212 | Inter-translocation |
| Chr8 | 13208224 | 13210533 | chr8 | 8722994 | 8725300 | Intra-translocation |
| Chr8 | 13280647 | 13275400 | chr9 | 28857753 | 28862951 | Inversion |
| Chr8 | 13389771 | 13390059 | chr8 | 8259008 | 8259301 | Intra-translocation |
| Chr8 | 13397578 | 13398049 | chr5 | 15154950 | 15155427 | Inter-translocation |
| Chr8 | 13415390 | 13415858 | chr9 | 29196915 | 29197380 | Inter-translocation |
| Chr8 | 13603644 | 13602240 | chr6 | 6628 | 8032 | Inversion |
| Chr8 | 13809444 | 13809161 | chr9 | 44564360 | 44564644 | Inversion |
| Chr8 | 13888661 | 13889370 | chr9 | 26516044 | 26516753 | Inter-translocation |
| Chr8 | 13974337 | 13974605 | chr8 | 25319680 | 25319947 | Intra-translocation |
| Chr8 | 14842381 | 14849815 | chr8 | 21946897 | 21954339 | Intra-translocation |
| Chr8 | 15042492 | 15043171 | chr9 | 34261753 | 34262432 | Inter-translocation |
| Chr8 | 15368193 | 15365569 | chr4 | 24881939 | 24884563 | Inversion |
| Chr8 | 15537245 | 15535436 | chr9 | 24260208 | 24262016 | Inversion |
| Chr8 | 15538004 | 15537506 | chr8 | 16495840 | 16496347 | Inversion |
| Chr8 | 16008890 | 16002345 | chr4 | 7042889 | 7049414 | Inversion |
| Chr8 | 16816152 | 16816516 | chr4 | 2242348 | 2242712 | Inter-translocation |
| Chr8 | 17313871 | 17270835 | chr8 | 18241090 | 18284191 | Inversion |
| Chr8 | 17344663 | 17312431 | chr8 | 18206805 | 18239030 | Inversion |
| Chr8 | 17414031 | 17352553 | chr8 | 18152008 | 18213472 | Inversion |
| Chr8 | 17427403 | 17414027 | chr1 | 25672292 | 25685653 | Inversion |
| Chr8 | 17459811 | 17427400 | chr8 | 18119623 | 18152012 | Inversion |
| Chr8 | 17485518 | 17460102 | chr8 | 18094203 | 18119622 | Inversion |
| Chr8 | 17486051 | 17485465 | chr8 | 18093621 | 18094207 | Inversion |
| Chr8 | 17507200 | 17509079 | chr8 | 20468102 | 20469982 | Intra-translocation |
| Chr8 | 17508588 | 17487601 | chr8 | 18072664 | 18093613 | Inversion |
| Chr8 | 17565893 | 17511316 | chr8 | 18019489 | 18074053 | Inversion |
| Chr8 | 17570031 | 17565887 | chr8 | 18008231 | 18012375 | Inversion |
| Chr8 | 17582669 | 17578205 | chr8 | 18003769 | 18008235 | Inversion |
| Chr8 | 17585372 | 17587250 | chr8 | 11066800 | 11068669 | Intra-translocation |
| Chr8 | 17587250 | 17589532 | chr4 | 35376223 | 35378496 | Inter-translocation |
| Chr8 | 17626373 | 17589908 | chr8 | 17967308 | 18003770 | Inversion |
| Chr8 | 17658728 | 17626368 | chr8 | 17928152 | 17960533 | Inversion |
| Chr8 | 17682715 | 17659628 | chr8 | 17904933 | 17928019 | Inversion |
| Chr8 | 17689907 | 17688079 | chr8 | 17904474 | 17906312 | Inversion |
| Chr8 | 17696111 | 17689929 | chr8 | 17896214 | 17902397 | Inversion |
| Chr8 | 17714147 | 17696128 | chr8 | 17877970 | 17895969 | Inversion |
| Chr8 | 17721077 | 17711426 | chr8 | 17862303 | 17871995 | Inversion |
| Chr8 | 17726519 | 17721397 | chr8 | 17857210 | 17862302 | Inversion |
| Chr8 | 17739476 | 17726512 | chr8 | 17835975 | 17849050 | Inversion |
| Chr8 | 17750330 | 17747538 | chr8 | 17833169 | 17835980 | Inversion |
| Chr8 | 17758124 | 17749580 | chr8 | 17803064 | 17811612 | Inversion |
| Chr8 | 17776376 | 17770292 | chr8 | 17795299 | 17801414 | Inversion |
| Chr8 | 17779808 | 17776586 | chr8 | 17802270 | 17805488 | Inversion |
| Chr8 | 17780658 | 17779995 | chr8 | 17801606 | 17802272 | Inversion |
| Chr8 | 17818411 | 17815545 | chr1 | 25889931 | 25892794 | Inversion |
| Chr8 | 17858583 | 17857589 | chr8 | 17792102 | 17793096 | Inversion |
| Chr8 | 17858977 | 17858804 | chr8 | 17790899 | 17791072 | Inversion |
| Chr8 | 17896502 | 17898270 | chr8 | 19257042 | 19258812 | Intra-translocation |
| Chr8 | 17950137 | 17948173 | chr3 | 22454399 | 22456356 | Inversion |
| Chr8 | 18000620 | 17999220 | chr8 | 25675088 | 25676487 | Inversion |
| Chr8 | 18019664 | 18018426 | chr8 | 5560738 | 5561974 | Inversion |
| Chr8 | 18041907 | 18041451 | chr8 | 22161927 | 22162383 | Inversion |
| Chr8 | 18156424 | 18157440 | chr9 | 21670981 | 21671996 | Inter-translocation |
| Chr8 | 18216549 | 18217192 | chr1 | 10503427 | 10504074 | Inter-translocation |
| Chr8 | 18231822 | 18231164 | chr9 | 29138478 | 29139136 | Inversion |
| Chr8 | 18243859 | 18233314 | chr8 | 18574994 | 18585504 | Inversion |
| Chr8 | 18253294 | 18244298 | chr8 | 18565887 | 18574844 | Inversion |
| Chr8 | 18255922 | 18253294 | chr8 | 18562303 | 18564932 | Inversion |
| Chr8 | 18267426 | 18261776 | chr8 | 18556656 | 18562307 | Inversion |
| Chr8 | 18271163 | 18267463 | chr8 | 18552463 | 18556164 | Inversion |
| Chr8 | 18272448 | 18271150 | chr8 | 18543061 | 18544357 | Inversion |
| Chr8 | 18272902 | 18272447 | chr8 | 18541631 | 18542086 | Inversion |
| Chr8 | 18274064 | 18272900 | chr8 | 18538828 | 18539991 | Inversion |
| Chr8 | 18275105 | 18272903 | chr8 | 13205322 | 13207529 | Inversion |
| Chr8 | 18285898 | 18274600 | chr8 | 18527544 | 18538830 | Inversion |
| Chr8 | 18287918 | 18288102 | chr4 | 4249562 | 4249745 | Inter-translocation |
| Chr8 | 18314076 | 18314410 | chr8 | 20082970 | 20083305 | Intra-translocation |
| Chr8 | 18417063 | 18418440 | chr9 | 31098035 | 31099412 | Inter-translocation |
| Chr8 | 18423805 | 18430185 | chr8 | 18691670 | 18698080 | Intra-translocation |
| Chr8 | 18584306 | 18582911 | chr3 | 42266426 | 42267821 | Inversion |
| Chr8 | 18655876 | 18658727 | chr5 | 14768968 | 14771845 | Inter-translocation |
| Chr8 | 18658730 | 18665509 | chr1 | 10186611 | 10193388 | Inter-translocation |
| Chr8 | 18730849 | 18731889 | chr9 | 28172743 | 28173794 | Inter-translocation |
| Chr8 | 18737064 | 18736829 | chr8 | 18760979 | 18761212 | Inversion |
| Chr8 | 18880060 | 18878863 | chr6 | 34444361 | 34445560 | Inversion |
| Chr8 | 19002875 | 19007555 | chr8 | 22557790 | 22562469 | Intra-translocation |
| Chr8 | 19042030 | 19050156 | chr8 | 18730973 | 18739104 | Intra-translocation |
| Chr8 | 19116196 | 19110636 | chr8 | 19403548 | 19409095 | Inversion |
| Chr8 | 19140883 | 19138352 | chr8 | 20486695 | 20489206 | Inversion |
| Chr8 | 19183002 | 19181838 | chr9 | 30898549 | 30899708 | Inversion |
| Chr8 | 19291484 | 19275483 | chr8 | 20974397 | 20990398 | Inversion |
| Chr8 | 19295211 | 19291653 | chr8 | 20970809 | 20974397 | Inversion |
| Chr8 | 19302227 | 19295605 | chr8 | 20964182 | 20970803 | Inversion |
| Chr8 | 19313125 | 19302223 | chr8 | 20952397 | 20963268 | Inversion |
| Chr8 | 19320310 | 19313126 | chr8 | 20937106 | 20944289 | Inversion |
| Chr8 | 19323593 | 19320320 | chr8 | 20933730 | 20937005 | Inversion |
| Chr8 | 19353376 | 19323589 | chr8 | 20902712 | 20932441 | Inversion |
| Chr8 | 19366436 | 19353328 | chr8 | 20889599 | 20902701 | Inversion |
| Chr8 | 19382918 | 19366995 | chr8 | 20873642 | 20889600 | Inversion |
| Chr8 | 19394671 | 19382913 | chr8 | 20858776 | 20870482 | Inversion |
| Chr8 | 19407565 | 19394663 | chr8 | 20845789 | 20858744 | Inversion |
| Chr8 | 19425705 | 19407565 | chr8 | 20827479 | 20845673 | Inversion |
| Chr8 | 19427235 | 19425825 | chr8 | 20826087 | 20827479 | Inversion |
| Chr8 | 19427901 | 19427232 | chr8 | 20825072 | 20825741 | Inversion |
| Chr8 | 19435156 | 19427897 | chr8 | 20817494 | 20824722 | Inversion |
| Chr8 | 19467916 | 19459045 | chr8 | 20809822 | 20818689 | Inversion |
| Chr8 | 19474485 | 19468046 | chr8 | 20790550 | 20796987 | Inversion |
| Chr8 | 19475221 | 19474481 | chr8 | 20776622 | 20777382 | Inversion |
| Chr8 | 19489667 | 19480751 | chr8 | 20768077 | 20776991 | Inversion |
| Chr8 | 19490982 | 19490023 | chr8 | 20767120 | 20768079 | Inversion |
| Chr8 | 19493118 | 19490979 | chr8 | 20764715 | 20766855 | Inversion |
| Chr8 | 19508731 | 19493126 | chr8 | 20748845 | 20764439 | Inversion |
| Chr8 | 19523068 | 19508723 | chr8 | 20732746 | 20747092 | Inversion |
| Chr8 | 19542439 | 19531189 | chr8 | 20721546 | 20732750 | Inversion |
| Chr8 | 19578046 | 19557338 | chr8 | 20692479 | 20713187 | Inversion |
| Chr8 | 19608075 | 19578041 | chr8 | 20649155 | 20679150 | Inversion |
| Chr8 | 19608681 | 19608076 | chr8 | 17831855 | 17832458 | Inversion |
| Chr8 | 19622095 | 19608680 | chr8 | 20635746 | 20649161 | Inversion |
| Chr8 | 19624780 | 19622419 | chr8 | 20633457 | 20635744 | Inversion |
| Chr8 | 19642295 | 19631907 | chr8 | 20623097 | 20633458 | Inversion |
| Chr8 | 19651538 | 19642290 | chr8 | 20613775 | 20623067 | Inversion |
| Chr8 | 19652847 | 19651597 | chr8 | 20611748 | 20612996 | Inversion |
| Chr8 | 19657970 | 19652845 | chr8 | 20593576 | 20598651 | Inversion |
| Chr8 | 19673955 | 19664157 | chr8 | 20583430 | 20593229 | Inversion |
| Chr8 | 19688685 | 19673953 | chr8 | 20567311 | 20582048 | Inversion |
| Chr8 | 19691506 | 19688893 | chr8 | 20564701 | 20567314 | Inversion |
| Chr8 | 19712900 | 19693753 | chr8 | 20545580 | 20564705 | Inversion |
| Chr8 | 19719675 | 19714109 | chr1 | 16658736 | 16664303 | Inversion |
| Chr8 | 19733904 | 19719676 | chr8 | 20531370 | 20545585 | Inversion |
| Chr8 | 19737081 | 19733840 | chr8 | 20528127 | 20531369 | Inversion |
| Chr8 | 19739793 | 19737854 | chr8 | 20523701 | 20525639 | Inversion |
| Chr8 | 19760105 | 19739788 | chr8 | 20501968 | 20522309 | Inversion |
| Chr8 | 19769968 | 19760235 | chr8 | 20492098 | 20501840 | Inversion |
| Chr8 | 19771656 | 19770892 | chr8 | 20486201 | 20486956 | Inversion |
| Chr8 | 19786502 | 19780740 | chr8 | 20480465 | 20486205 | Inversion |
| Chr8 | 19840838 | 19815729 | chr8 | 20443022 | 20468101 | Inversion |
| Chr8 | 19854173 | 19861059 | chr6 | 11239929 | 11246791 | Inter-translocation |
| Chr8 | 19860568 | 19851808 | chr8 | 20426994 | 20435763 | Inversion |
| Chr8 | 19862317 | 19860927 | chr8 | 10248805 | 10250196 | Inversion |
| Chr8 | 19879947 | 19861294 | chr8 | 20408350 | 20426991 | Inversion |
| Chr8 | 19885302 | 19879941 | chr8 | 20356661 | 20362022 | Inversion |
| Chr8 | 19898487 | 19887241 | chr8 | 20347341 | 20358584 | Inversion |
| Chr8 | 19906768 | 19898494 | chr8 | 20338876 | 20347104 | Inversion |
| Chr8 | 19929014 | 19915181 | chr8 | 20324149 | 20338002 | Inversion |
| Chr8 | 19937172 | 19929618 | chr8 | 20316529 | 20324153 | Inversion |
| Chr8 | 19943951 | 19943349 | chr8 | 18423259 | 18423861 | Inversion |
| Chr8 | 19968918 | 19943952 | chr8 | 20291601 | 20316535 | Inversion |
| Chr8 | 19990650 | 19968916 | chr8 | 20256750 | 20278487 | Inversion |
| Chr8 | 19991396 | 19990607 | chr8 | 20255966 | 20256753 | Inversion |
| Chr8 | 19999891 | 19991606 | chr8 | 20247589 | 20255865 | Inversion |
| Chr8 | 20015527 | 20016048 | chr3 | 7089581 | 7090102 | Inter-translocation |
| Chr8 | 20017447 | 20017276 | chr2 | 43174254 | 43174425 | Inversion |
| Chr8 | 20021720 | 20022340 | chr8 | 20401106 | 20401726 | Intra-translocation |
| Chr8 | 20046849 | 20048229 | chr9 | 32740008 | 32741408 | Inter-translocation |
| Chr8 | 20056270 | 20056017 | chr3 | 13884455 | 13884708 | Inversion |
| Chr8 | 20061388 | 20061249 | chr6 | 35945076 | 35945215 | Inversion |
| Chr8 | 20079740 | 20072239 | chr8 | 15054151 | 15061630 | Inversion |
| Chr8 | 20128884 | 20127692 | chr9 | 31183959 | 31185160 | Inversion |
| Chr8 | 20135539 | 20132285 | chr9 | 21797227 | 21800482 | Inversion |
| Chr8 | 20138857 | 20144503 | chr2 | 12652572 | 12658223 | Inter-translocation |
| Chr8 | 20151853 | 20154085 | chr9 | 26663272 | 26665504 | Inter-translocation |
| Chr8 | 20180574 | 20183226 | chr9 | 41134514 | 41137172 | Inter-translocation |
| Chr8 | 20204631 | 20207129 | chr5 | 16042129 | 16044560 | Inter-translocation |
| Chr8 | 20231380 | 20231724 | chr3 | 13373981 | 13374323 | Inter-translocation |
| Chr8 | 20236544 | 20236753 | chr3 | 13375125 | 13375333 | Inter-translocation |
| Chr8 | 20249921 | 20249179 | chr5 | 25701948 | 25702686 | Inversion |
| Chr8 | 20253158 | 20249922 | chr5 | 25096253 | 25099462 | Inversion |
| Chr8 | 20255876 | 20253717 | chr5 | 25696834 | 25698983 | Inversion |
| Chr8 | 20261937 | 20259052 | chr5 | 25693984 | 25696839 | Inversion |
| Chr8 | 20273602 | 20268841 | chr8 | 20242833 | 20247588 | Inversion |
| Chr8 | 20286116 | 20273534 | chr8 | 20230247 | 20242832 | Inversion |
| Chr8 | 20288100 | 20286112 | chr8 | 20217321 | 20219309 | Inversion |
| Chr8 | 20310523 | 20310651 | chr3 | 6231905 | 6232033 | Inter-translocation |
| Chr8 | 20314214 | 20311176 | chr8 | 4782813 | 4785866 | Inversion |
| Chr8 | 20348274 | 20351489 | chr5 | 14215820 | 14219048 | Inter-translocation |
| Chr8 | 20409672 | 20428424 | chr8 | 19944219 | 19963072 | Intra-translocation |
| Chr8 | 20431127 | 20444309 | chr8 | 19980794 | 19993972 | Intra-translocation |
| Chr8 | 20467452 | 20485885 | chr8 | 20002133 | 20020622 | Intra-translocation |
| Chr8 | 20503633 | 20499839 | chr8 | 20335081 | 20338876 | Inversion |
| Chr8 | 20577529 | 20565320 | chr8 | 19932002 | 19944222 | Inversion |
| Chr8 | 20598497 | 20597803 | chr8 | 19931199 | 19931894 | Inversion |
| Chr8 | 20598498 | 20604944 | chr8 | 19849999 | 19856440 | Intra-translocation |
| Chr8 | 20654282 | 20608862 | chr8 | 19885794 | 19931205 | Inversion |
| Chr8 | 20658965 | 20654278 | chr8 | 19880109 | 19884777 | Inversion |
| Chr8 | 20668548 | 20662957 | chr8 | 19862439 | 19868048 | Inversion |
| Chr8 | 20670396 | 20668883 | chr8 | 19860825 | 19862335 | Inversion |
| Chr8 | 20673638 | 20672888 | chr8 | 19860074 | 19860823 | Inversion |
| Chr8 | 20682071 | 20673630 | chr8 | 19841591 | 19850000 | Inversion |
| Chr8 | 20684076 | 20682054 | chr8 | 19839045 | 19841064 | Inversion |
| Chr8 | 20685684 | 20684178 | chr8 | 19837445 | 19838944 | Inversion |
| Chr8 | 20691064 | 20685737 | chr8 | 19831510 | 19836821 | Inversion |
| Chr8 | 20695292 | 20691060 | chr8 | 19824369 | 19828603 | Inversion |
| Chr8 | 20696205 | 20695287 | chr8 | 19822053 | 19822983 | Inversion |
| Chr8 | 20708604 | 20696204 | chr8 | 19809702 | 19822114 | Inversion |
| Chr8 | 20711307 | 20708569 | chr8 | 19806962 | 19809700 | Inversion |
| Chr8 | 20717435 | 20711315 | chr8 | 19800217 | 19806340 | Inversion |
| Chr8 | 20722737 | 20717458 | chr8 | 19794835 | 19800114 | Inversion |
| Chr8 | 20728329 | 20723068 | chr8 | 19789550 | 19794810 | Inversion |
| Chr8 | 20733019 | 20728325 | chr8 | 19779131 | 19783824 | Inversion |
| Chr8 | 20735911 | 20733014 | chr8 | 19755961 | 19758871 | Inversion |
| Chr8 | 20744021 | 20735912 | chr9 | 22639115 | 22647240 | Inversion |
| Chr8 | 20749848 | 20744022 | chr8 | 19750145 | 19755965 | Inversion |
| Chr8 | 20757468 | 20750060 | chr8 | 19735752 | 19743165 | Inversion |
| Chr8 | 20760307 | 20757824 | chr8 | 19733277 | 19735754 | Inversion |
| Chr8 | 20761643 | 20760344 | chr8 | 19730126 | 19731429 | Inversion |
| Chr8 | 20770353 | 20762100 | chr8 | 19721846 | 19730120 | Inversion |
| Chr8 | 20789174 | 20770406 | chr8 | 19703080 | 19721853 | Inversion |
| Chr8 | 20792742 | 20789164 | chr8 | 19696118 | 19699698 | Inversion |
| Chr8 | 20795833 | 20792748 | chr8 | 19690853 | 19693938 | Inversion |
| Chr8 | 20830083 | 20803918 | chr8 | 19664642 | 19690859 | Inversion |
| Chr8 | 20831618 | 20830533 | chr8 | 19663559 | 19664644 | Inversion |
| Chr8 | 20833260 | 20831615 | chr8 | 19659859 | 19661507 | Inversion |
| Chr8 | 20837920 | 20833511 | chr8 | 19655445 | 19659853 | Inversion |
| Chr8 | 20846825 | 20840232 | chr8 | 19647683 | 19654268 | Inversion |
| Chr8 | 21059144 | 21056929 | chr8 | 21189596 | 21191810 | Inversion |
| Chr8 | 21098678 | 21097492 | chr6 | 34563518 | 34564705 | Inversion |
| Chr8 | 21350432 | 21344191 | chr6 | 22281545 | 22287787 | Inversion |
| Chr8 | 21369884 | 21373853 | chr8 | 19439611 | 19443580 | Intra-translocation |
| Chr8 | 21624279 | 21627822 | chr9 | 30851661 | 30855213 | Inter-translocation |
| Chr8 | 21943491 | 21944887 | chr8 | 16135231 | 16136633 | Intra-translocation |
| Chr8 | 22007516 | 22002009 | chr9 | 30462626 | 30468143 | Inversion |
| Chr8 | 22055867 | 22053389 | chr9 | 30524474 | 30526951 | Inversion |
| Chr8 | 22086647 | 22084963 | chr9 | 28589509 | 28591185 | Inversion |
| Chr8 | 22089744 | 22092838 | chr8 | 11399908 | 11403002 | Intra-translocation |
| Chr8 | 22108436 | 22108088 | chr9 | 41410808 | 41411153 | Inversion |
| Chr8 | 22117161 | 22119204 | chr8 | 18743740 | 18745736 | Intra-translocation |
| Chr8 | 22300686 | 22301290 | chr5 | 21283101 | 21283702 | Inter-translocation |
| Chr8 | 22386457 | 22386800 | chr8 | 6973198 | 6973541 | Intra-translocation |
| Chr8 | 22409276 | 22414204 | chr8 | 21082832 | 21087757 | Intra-translocation |
| Chr8 | 22442443 | 22442988 | chr8 | 20362250 | 20362795 | Intra-translocation |
| Chr8 | 22447676 | 22450346 | chr5 | 14209209 | 14211883 | Inter-translocation |
| Chr8 | 22467000 | 22464439 | chr5 | 25099398 | 25101970 | Inversion |
| Chr8 | 22501739 | 22501472 | chr8 | 21294628 | 21294899 | Inversion |
| Chr8 | 22504991 | 22505622 | chr8 | 13935898 | 13936528 | Intra-translocation |
| Chr8 | 22582749 | 22579927 | chr5 | 15332487 | 15335322 | Inversion |
| Chr8 | 22582885 | 22582744 | chr5 | 15324178 | 15324319 | Inversion |
| Chr8 | 22624486 | 22625095 | chr9 | 26740863 | 26741484 | Inter-translocation |
| Chr8 | 22663685 | 22663884 | chr2 | 28194362 | 28194560 | Inter-translocation |
| Chr8 | 22713900 | 22713667 | chr8 | 13009401 | 13009626 | Inversion |
| Chr8 | 22714245 | 22714479 | chr3 | 8377941 | 8378174 | Inter-translocation |
| Chr8 | 22733365 | 22733693 | chr8 | 7334758 | 7335087 | Intra-translocation |
| Chr8 | 22766831 | 22765117 | chr8 | 38780301 | 38782015 | Inversion |
| Chr8 | 22808683 | 22809813 | chr8 | 17784698 | 17785831 | Intra-translocation |
| Chr8 | 22911321 | 22909959 | chr5 | 16280994 | 16282357 | Inversion |
| Chr8 | 23157987 | 23157713 | chr3 | 12436598 | 12436873 | Inversion |
| Chr8 | 23212340 | 23198992 | chr8 | 10131630 | 10144976 | Inversion |
| Chr8 | 23256578 | 23258612 | chr8 | 8623242 | 8625274 | Intra-translocation |
| Chr8 | 23283719 | 23282543 | chr3 | 7113670 | 7114854 | Inversion |
| Chr8 | 23406872 | 23404914 | chr8 | 8937340 | 8939298 | Inversion |
| Chr8 | 23415823 | 23415993 | chr8 | 9966334 | 9966504 | Intra-translocation |
| Chr8 | 23536544 | 23535483 | chr5 | 16305209 | 16306269 | Inversion |
| Chr8 | 23621632 | 23620399 | chr8 | 9907398 | 9908603 | Inversion |
| Chr8 | 23624672 | 23624566 | chr3 | 49550029 | 49550135 | Inversion |
| Chr8 | 23628196 | 23628411 | chr3 | 23269337 | 23269552 | Inter-translocation |
| Chr8 | 23645526 | 23650439 | chr8 | 5332993 | 5337903 | Intra-translocation |
| Chr8 | 23705443 | 23705651 | chr3 | 8297559 | 8297767 | Inter-translocation |
| Chr8 | 24265898 | 24266279 | chr8 | 25386669 | 25387050 | Intra-translocation |
| Chr8 | 25414375 | 25415134 | chr8 | 25375140 | 25375878 | Intra-translocation |
| Chr8 | 25426745 | 25432593 | chr8 | 25380892 | 25386668 | Intra-translocation |
| Chr8 | 25437941 | 25434802 | chr9 | 29930522 | 29933655 | Inversion |
| Chr8 | 25444871 | 25439433 | chr3 | 44566974 | 44572410 | Inversion |
| Chr8 | 25452877 | 25462760 | chr8 | 25394499 | 25404426 | Intra-translocation |
| Chr8 | 25472306 | 25472044 | chr8 | 38718192 | 38718454 | Inversion |
| Chr8 | 25472315 | 25472491 | chr8 | 25414684 | 25414860 | Intra-translocation |
| Chr8 | 25472482 | 25472844 | chr8 | 25418044 | 25418406 | Intra-translocation |
| Chr8 | 25478209 | 25474679 | chr6 | 30620554 | 30624046 | Inversion |
| Chr8 | 25509770 | 25512222 | chr8 | 25332279 | 25334740 | Intra-translocation |
| Chr8 | 25512318 | 25513652 | chr8 | 25334740 | 25336075 | Intra-translocation |
| Chr8 | 25515463 | 25515242 | chr8 | 25371819 | 25372040 | Inversion |
| Chr8 | 25521262 | 25522087 | chr8 | 25340202 | 25341027 | Intra-translocation |
| Chr8 | 25526244 | 25525103 | chr8 | 25370695 | 25371817 | Inversion |
| Chr8 | 25527884 | 25525946 | chr8 | 25368207 | 25370128 | Inversion |
| Chr8 | 25543374 | 25543723 | chr9 | 543930 | 544278 | Inter-translocation |
| Chr8 | 25553635 | 25550871 | chr8 | 25319949 | 25322696 | Inversion |
| Chr8 | 25563446 | 25553633 | chr8 | 25272857 | 25282646 | Inversion |
| Chr8 | 25565015 | 25563452 | chr8 | 25368204 | 25369764 | Inversion |
| Chr8 | 25585697 | 25585192 | chr8 | 25339982 | 25340486 | Inversion |
| Chr8 | 25595262 | 25594408 | chr8 | 25339113 | 25339966 | Inversion |
| Chr8 | 25595561 | 25595408 | chr8 | 25338280 | 25338432 | Inversion |
| Chr8 | 25599232 | 25595658 | chr8 | 25341025 | 25344592 | Inversion |
| Chr8 | 25612108 | 25611731 | chr8 | 38985052 | 38985428 | Inversion |
| Chr8 | 25614580 | 25612113 | chr8 | 38982376 | 38984770 | Inversion |
| Chr8 | 25621308 | 25619712 | chr8 | 25365668 | 25367261 | Inversion |
| Chr8 | 25625054 | 25622859 | chr8 | 25363481 | 25365676 | Inversion |
| Chr8 | 25647235 | 25649199 | chr3 | 4361828 | 4363789 | Inter-translocation |
| Chr8 | 25664780 | 25669687 | chr8 | 22794735 | 22799604 | Intra-translocation |
| Chr8 | 25669916 | 25670992 | chr3 | 13320524 | 13321618 | Inter-translocation |
| Chr8 | 25710263 | 25709796 | chr8 | 25330898 | 25331365 | Inversion |
| Chr8 | 25731488 | 25731653 | chr8 | 25355567 | 25355723 | Intra-translocation |
| Chr8 | 25807950 | 25805339 | chr9 | 31319614 | 31322231 | Inversion |
| Chr8 | 25835328 | 25836001 | chr5 | 16286342 | 16287014 | Inter-translocation |
| Chr8 | 25835338 | 25832243 | chr2 | 12668591 | 12671671 | Inversion |
| Chr8 | 25837080 | 25836569 | chr8 | 6071783 | 6072294 | Inversion |
| Chr8 | 25838845 | 25837261 | chr8 | 39612798 | 39614380 | Inversion |
| Chr8 | 25871348 | 25871617 | chr8 | 25488656 | 25488916 | Intra-translocation |
| Chr8 | 25871616 | 25872190 | chr4 | 2369694 | 2370264 | Inter-translocation |
| Chr8 | 25872459 | 25873136 | chr8 | 25488914 | 25489589 | Intra-translocation |
| Chr8 | 25873216 | 25873964 | chr8 | 25489872 | 25490643 | Intra-translocation |
| Chr8 | 25876210 | 25877597 | chr8 | 25495476 | 25496867 | Intra-translocation |
| Chr8 | 25884893 | 25879805 | chr8 | 3165214 | 3170297 | Inversion |
| Chr8 | 25947339 | 25947124 | chr3 | 49519676 | 49519892 | Inversion |
| Chr8 | 26067147 | 26066904 | chr5 | 24591618 | 24591862 | Inversion |
| Chr8 | 26252700 | 26252951 | chr8 | 25904971 | 25905220 | Intra-translocation |
| Chr8 | 26291085 | 26293088 | chr9 | 21382347 | 21384349 | Inter-translocation |
| Chr8 | 26293165 | 26294397 | chr9 | 21384477 | 21385709 | Inter-translocation |
| Chr8 | 26322863 | 26322514 | chr4 | 4244896 | 4245245 | Inversion |
| Chr8 | 26430918 | 26431764 | chr8 | 22697402 | 22698249 | Intra-translocation |
| Chr8 | 26474883 | 26478818 | chr5 | 15134401 | 15138336 | Inter-translocation |
| Chr8 | 26516579 | 26517656 | chr8 | 29711460 | 29712540 | Intra-translocation |
| Chr8 | 26517777 | 26523214 | chr8 | 27748811 | 27754272 | Intra-translocation |
| Chr8 | 26610369 | 26609396 | chr8 | 18803922 | 18804895 | Inversion |
| Chr8 | 26673779 | 26673950 | chr6 | 1030396 | 1030567 | Inter-translocation |
| Chr8 | 26724097 | 26732882 | chr9 | 27532100 | 27540873 | Inter-translocation |
| Chr8 | 26738988 | 26739586 | chr5 | 15726424 | 15727001 | Inter-translocation |
| Chr8 | 26878119 | 26880360 | chr8 | 26368010 | 26370251 | Intra-translocation |
| Chr8 | 26955145 | 26955480 | chr8 | 12598315 | 12598639 | Intra-translocation |
| Chr8 | 27032322 | 27029908 | chr8 | 27645262 | 27647657 | Inversion |
| Chr8 | 27068231 | 27065748 | chr8 | 18292359 | 18294844 | Inversion |
| Chr8 | 27068297 | 27066676 | chr8 | 4493863 | 4495479 | Inversion |
| Chr8 | 27068310 | 27069812 | chr8 | 17770340 | 17771842 | Intra-translocation |
| Chr8 | 27071214 | 27074045 | chr8 | 17771837 | 17774664 | Intra-translocation |
| Chr8 | 27098431 | 27101925 | chr8 | 26725384 | 26728852 | Intra-translocation |
| Chr8 | 27240220 | 27241477 | chr8 | 7916551 | 7917808 | Intra-translocation |
| Chr8 | 27244196 | 27240354 | chr3 | 34742897 | 34746738 | Inversion |
| Chr8 | 27245555 | 27244657 | chr8 | 3004090 | 3004989 | Inversion |
| Chr8 | 27726254 | 27722276 | chr1 | 2684827 | 2688804 | Inversion |
| Chr8 | 27737316 | 27737544 | chr1 | 2676606 | 2676834 | Inter-translocation |
| Chr8 | 28001094 | 28004262 | chr8 | 12441781 | 12444947 | Intra-translocation |
| Chr8 | 28005102 | 28004541 | chr3 | 42334696 | 42335259 | Inversion |
| Chr8 | 28005263 | 28005412 | chr8 | 10892703 | 10892853 | Intra-translocation |
| Chr8 | 28059641 | 28059536 | chr8 | 5103602 | 5103707 | Inversion |
| Chr8 | 28280233 | 28280957 | chr8 | 9150395 | 9151123 | Intra-translocation |
| Chr8 | 28311248 | 28312998 | chr6 | 1544496 | 1546261 | Inter-translocation |
| Chr8 | 28311423 | 28313446 | chr9 | 27807349 | 27809441 | Inter-translocation |
| Chr8 | 28313186 | 28313503 | chr6 | 1546316 | 1546627 | Inter-translocation |
| Chr8 | 28317550 | 28317675 | chr5 | 14262272 | 14262397 | Inter-translocation |
| Chr8 | 28337344 | 28336078 | chr6 | 2492879 | 2494096 | Inversion |
| Chr8 | 29100170 | 29096397 | chr8 | 9617665 | 9621466 | Inversion |
| Chr8 | 29245603 | 29245695 | chr8 | 9838654 | 9838746 | Intra-translocation |
| Chr8 | 29245614 | 29245457 | chr8 | 3551628 | 3551785 | Inversion |
| Chr8 | 29658304 | 29658649 | chr3 | 14096244 | 14096589 | Inter-translocation |
| Chr8 | 29695030 | 29695199 | chr5 | 23570179 | 23570348 | Inter-translocation |
| Chr8 | 29696389 | 29696616 | chr8 | 11271063 | 11271291 | Intra-translocation |
| Chr8 | 29739283 | 29739079 | chr8 | 36934767 | 36934971 | Inversion |
| Chr8 | 29757501 | 29759804 | chr8 | 29242228 | 29244528 | Intra-translocation |
| Chr8 | 29759801 | 29762659 | chr8 | 29245356 | 29248205 | Intra-translocation |
| Chr8 | 29789149 | 29793480 | chr8 | 29171856 | 29176191 | Intra-translocation |
| Chr8 | 29793485 | 29797263 | chr8 | 29176316 | 29180023 | Intra-translocation |
| Chr8 | 29797257 | 29799216 | chr8 | 29180443 | 29182438 | Intra-translocation |
| Chr8 | 29812983 | 29815310 | chr8 | 29182433 | 29184766 | Intra-translocation |
| Chr8 | 29815425 | 29817094 | chr8 | 29184764 | 29186429 | Intra-translocation |
| Chr8 | 29870829 | 29869963 | chr8 | 30095925 | 30096792 | Inversion |
| Chr8 | 29986376 | 29985124 | chr8 | 29413903 | 29415158 | Inversion |
| Chr8 | 29987115 | 29986372 | chr8 | 29412804 | 29413556 | Inversion |
| Chr8 | 29990826 | 29987707 | chr8 | 29406636 | 29409792 | Inversion |
| Chr8 | 30022587 | 30020213 | chr4 | 6922619 | 6924993 | Inversion |
| Chr8 | 30041009 | 30034229 | chr9 | 27106654 | 27113440 | Inversion |
| Chr8 | 30116002 | 30117289 | chr8 | 29692513 | 29693824 | Intra-translocation |
| Chr8 | 30125540 | 30126348 | chr8 | 29786638 | 29787446 | Intra-translocation |
| Chr8 | 30185786 | 30186379 | chr5 | 22695880 | 22696473 | Inter-translocation |
| Chr8 | 30396030 | 30396755 | chr6 | 1333045 | 1333747 | Inter-translocation |
| Chr8 | 30429927 | 30430723 | chr9 | 33373732 | 33374500 | Inter-translocation |
| Chr8 | 30430685 | 30425153 | chr3 | 26423893 | 26429401 | Inversion |
| Chr8 | 30461268 | 30463140 | chr1 | 17369219 | 17371092 | Inter-translocation |
| Chr8 | 30462438 | 30463974 | chr8 | 11189738 | 11191272 | Intra-translocation |
| Chr8 | 30464848 | 30464398 | chr6 | 24103801 | 24104251 | Inversion |
| Chr8 | 30502778 | 30502967 | chr9 | 19370395 | 19370581 | Inter-translocation |
| Chr8 | 30505293 | 30506643 | chr8 | 25491380 | 25492730 | Intra-translocation |
| Chr8 | 30512170 | 30513396 | chr8 | 25494180 | 25495405 | Intra-translocation |
| Chr8 | 30531673 | 30532218 | chr9 | 30288610 | 30289142 | Inter-translocation |
| Chr8 | 30532225 | 30532408 | chr6 | 21432042 | 21432226 | Inter-translocation |
| Chr8 | 30532410 | 30532578 | chr6 | 21434709 | 21434880 | Inter-translocation |
| Chr8 | 30532723 | 30534095 | chr9 | 30289737 | 30291108 | Inter-translocation |
| Chr8 | 30553962 | 30553703 | chr5 | 24091576 | 24091835 | Inversion |
| Chr8 | 30579623 | 30579985 | chr8 | 13822967 | 13823329 | Intra-translocation |
| Chr8 | 30767595 | 30770512 | chr8 | 38532652 | 38535559 | Intra-translocation |
| Chr8 | 30770513 | 30773867 | chr8 | 20389503 | 20392863 | Intra-translocation |
| Chr8 | 30874972 | 30875116 | chr8 | 5085073 | 5085216 | Intra-translocation |
| Chr8 | 30896419 | 30897274 | chr9 | 31031414 | 31032290 | Inter-translocation |
| Chr8 | 30925826 | 30926135 | chr8 | 5887441 | 5887758 | Intra-translocation |
| Chr8 | 30957085 | 30956876 | chr9 | 19778963 | 19779170 | Inversion |
| Chr8 | 31000077 | 30998933 | chr7 | 30024760 | 30025899 | Inversion |
| Chr8 | 31115399 | 31120944 | chr8 | 30664672 | 30670217 | Intra-translocation |
| Chr8 | 31121010 | 31137857 | chr8 | 30670342 | 30687160 | Intra-translocation |
| Chr8 | 31887753 | 31890664 | chr8 | 31451119 | 31454044 | Intra-translocation |
| Chr8 | 31978365 | 31989001 | chr8 | 31531081 | 31541712 | Intra-translocation |
| Chr8 | 32004036 | 32007766 | chr7 | 30956010 | 30959748 | Inter-translocation |
| Chr8 | 32724224 | 32725161 | chr8 | 39713338 | 39714273 | Intra-translocation |
| Chr8 | 33694253 | 33693856 | chr9 | 40566171 | 40566572 | Inversion |
| Chr8 | 34051886 | 34051537 | chr8 | 30456814 | 30457165 | Inversion |
| Chr8 | 34138448 | 34139191 | chr8 | 25358395 | 25359096 | Intra-translocation |
| Chr8 | 34171715 | 34171312 | chr8 | 3402257 | 3402667 | Inversion |
| Chr8 | 34624244 | 34624747 | chr2 | 42488443 | 42488931 | Inter-translocation |
| Chr8 | 34625511 | 34624455 | chr1 | 10469614 | 10470670 | Inversion |
| Chr8 | 34630115 | 34625511 | chr1 | 10464897 | 10469500 | Inversion |
| Chr8 | 34640236 | 34630950 | chr1 | 10455609 | 10464896 | Inversion |
| Chr8 | 35135945 | 35135732 | chr8 | 34631715 | 34631928 | Inversion |
| Chr8 | 35682082 | 35681639 | chr8 | 38732742 | 38733183 | Inversion |
| Chr8 | 36072294 | 36072566 | chr8 | 37696994 | 37697265 | Intra-translocation |
| Chr8 | 36079407 | 36084053 | chr3 | 9141228 | 9145787 | Inter-translocation |
| Chr8 | 36162397 | 36177147 | chr8 | 13486747 | 13501498 | Intra-translocation |
| Chr8 | 36177272 | 36182203 | chr8 | 13501499 | 13506430 | Intra-translocation |
| Chr8 | 36183066 | 36182548 | chr2 | 7969903 | 7970421 | Inversion |
| Chr8 | 36371312 | 36371543 | chr8 | 8525951 | 8526181 | Intra-translocation |
| Chr8 | 36745254 | 36744853 | chr8 | 4362211 | 4362612 | Inversion |
| Chr8 | 37062464 | 37062233 | chr8 | 2673305 | 2673536 | Inversion |
| Chr8 | 37195645 | 37194593 | chr8 | 9547185 | 9548251 | Inversion |
| Chr8 | 37298074 | 37298457 | chr3 | 13896927 | 13897306 | Inter-translocation |
| Chr8 | 37328000 | 37327741 | chr7 | 23374962 | 23375221 | Inversion |
| Chr8 | 37347601 | 37347336 | chr8 | 20766855 | 20767120 | Inversion |
| Chr8 | 37537324 | 37538102 | chr9 | 31136533 | 31137310 | Inter-translocation |
| Chr8 | 37926361 | 37926239 | chr1 | 24641487 | 24641611 | Inversion |
| Chr8 | 37936980 | 37936613 | chr8 | 37618898 | 37619254 | Inversion |
| Chr8 | 37939872 | 37938792 | chr8 | 38780937 | 38782021 | Inversion |
| Chr8 | 37940240 | 37942591 | chr8 | 30541878 | 30544233 | Intra-translocation |
| Chr8 | 37978768 | 37976655 | chr7 | 26660184 | 26662297 | Inversion |
| Chr8 | 38017109 | 38020899 | chr8 | 30490480 | 30494290 | Intra-translocation |
| Chr8 | 38029100 | 38031257 | chr8 | 37610020 | 37612239 | Intra-translocation |
| Chr8 | 38031259 | 38029689 | chr2 | 3051667 | 3053241 | Inversion |
| Chr8 | 38033115 | 38033335 | chr8 | 37612245 | 37612465 | Intra-translocation |
| Chr8 | 38033823 | 38033368 | chr4 | 10244318 | 10244773 | Inversion |
| Chr8 | 38033986 | 38034628 | chr8 | 37612480 | 37613144 | Intra-translocation |
| Chr8 | 38036388 | 38034680 | chr8 | 34917500 | 34919244 | Inversion |
| Chr8 | 38037468 | 38037292 | chr8 | 37506351 | 37506523 | Inversion |
| Chr8 | 38088781 | 38087926 | chr1 | 41678635 | 41679488 | Inversion |
| Chr8 | 38118142 | 38118372 | chr9 | 23199345 | 23199571 | Inter-translocation |
| Chr8 | 38145733 | 38142545 | chr2 | 47959436 | 47962639 | Inversion |
| Chr8 | 38207711 | 38208016 | chr8 | 37797208 | 37797507 | Intra-translocation |
| Chr8 | 38207944 | 38208093 | chr8 | 37797549 | 37797704 | Intra-translocation |
| Chr8 | 38210021 | 38208957 | chr8 | 38109157 | 38110232 | Inversion |
| Chr8 | 38223887 | 38223973 | chr8 | 37774263 | 37774349 | Intra-translocation |
| Chr8 | 38236645 | 38235947 | chr8 | 38108145 | 38108836 | Inversion |
| Chr8 | 38239196 | 38239357 | chr2 | 43869213 | 43869374 | Inter-translocation |
| Chr8 | 38251047 | 38249421 | chr3 | 47139609 | 47141237 | Inversion |
| Chr8 | 38252852 | 38253075 | chr8 | 37696221 | 37696440 | Intra-translocation |
| Chr8 | 38258586 | 38258864 | chr8 | 37697843 | 37698114 | Intra-translocation |
| Chr8 | 38260532 | 38260076 | chr8 | 38110365 | 38110819 | Inversion |
| Chr8 | 38273382 | 38273745 | chr9 | 29533082 | 29533445 | Inter-translocation |
| Chr8 | 38350603 | 38350276 | chr5 | 16076656 | 16076980 | Inversion |
| Chr8 | 38372751 | 38372491 | chr8 | 5152599 | 5152860 | Inversion |
| Chr8 | 38411362 | 38411671 | chr2 | 41326571 | 41326880 | Inter-translocation |
| Chr8 | 38442616 | 38442178 | chr8 | 38054667 | 38055090 | Inversion |
| Chr8 | 38466638 | 38466823 | chr8 | 38092628 | 38092803 | Intra-translocation |
| Chr8 | 38467074 | 38467732 | chr8 | 38092995 | 38093649 | Intra-translocation |
| Chr8 | 38470794 | 38469108 | chr8 | 38049249 | 38050901 | Inversion |
| Chr8 | 38487541 | 38488182 | chr8 | 38123026 | 38123660 | Intra-translocation |
| Chr8 | 38556151 | 38555871 | chr1 | 26090268 | 26090548 | Inversion |
| Chr8 | 38559623 | 38560362 | chr8 | 29488256 | 29488991 | Intra-translocation |
| Chr8 | 38559681 | 38563449 | chr8 | 29530587 | 29534348 | Intra-translocation |
| Chr8 | 38645801 | 38644788 | chr8 | 38310067 | 38311096 | Inversion |
| Chr8 | 38649012 | 38645802 | chr8 | 38306611 | 38309722 | Inversion |
| Chr8 | 38656023 | 38647859 | chr8 | 38285101 | 38293300 | Inversion |
| Chr8 | 38776611 | 38776774 | chr8 | 29496269 | 29496434 | Intra-translocation |
| Chr8 | 38777754 | 38778042 | chr8 | 29508044 | 29508337 | Intra-translocation |
| Chr8 | 38806739 | 38806565 | chr8 | 38402669 | 38402843 | Inversion |
| Chr8 | 38884551 | 38884839 | chr8 | 36807914 | 36808202 | Intra-translocation |
| Chr8 | 38905695 | 38905338 | chr8 | 38605639 | 38605996 | Inversion |
| Chr8 | 38906307 | 38907344 | chr9 | 21591795 | 21592831 | Inter-translocation |
| Chr8 | 38906308 | 38905692 | chr8 | 38604749 | 38605365 | Inversion |
| Chr8 | 38940191 | 38940447 | chr8 | 10053796 | 10054056 | Intra-translocation |
| Chr8 | 38945752 | 38945920 | chr8 | 37661648 | 37661817 | Intra-translocation |
| Chr8 | 38951375 | 38952189 | chr3 | 13446652 | 13447455 | Inter-translocation |
| Chr8 | 38953445 | 38952197 | chr8 | 13046279 | 13047539 | Inversion |
| Chr8 | 39018737 | 39018362 | chr8 | 30269819 | 30270194 | Inversion |
| Chr8 | 39033440 | 39034237 | chr8 | 38960658 | 38961442 | Intra-translocation |
| Chr8 | 39034685 | 39039366 | chr8 | 38961571 | 38966197 | Intra-translocation |
| Chr8 | 39065002 | 39066389 | chr4 | 37635776 | 37637214 | Inter-translocation |
| Chr8 | 39065003 | 39063579 | chr8 | 30310001 | 30311423 | Inversion |
| Chr8 | 39069284 | 39069453 | chr8 | 38854769 | 38854943 | Intra-translocation |
| Chr8 | 39121946 | 39119711 | chr8 | 38828143 | 38830386 | Inversion |
| Chr8 | 39122808 | 39122640 | chr8 | 2499534 | 2499701 | Inversion |
| Chr8 | 39124775 | 39122806 | chr8 | 38918832 | 38920812 | Inversion |
| Chr8 | 39124937 | 39124782 | chr8 | 38826281 | 38826436 | Inversion |
| Chr8 | 39127845 | 39125842 | chr8 | 38830476 | 38832480 | Inversion |
| Chr8 | 39129089 | 39128992 | chr8 | 38830381 | 38830478 | Inversion |
| Chr8 | 39158830 | 39159045 | chr8 | 29434423 | 29434639 | Intra-translocation |
| Chr8 | 39161580 | 39161754 | chr8 | 29548967 | 29549140 | Intra-translocation |
| Chr8 | 39169415 | 39169075 | chr8 | 29549673 | 29550010 | Inversion |
| Chr8 | 39170398 | 39170671 | chr8 | 29553247 | 29553513 | Intra-translocation |
| Chr8 | 39170517 | 39170355 | chr8 | 38942892 | 38943047 | Inversion |
| Chr8 | 39183144 | 39184492 | chr8 | 38740602 | 38741948 | Intra-translocation |
| Chr8 | 39288934 | 39288649 | chr8 | 36806345 | 36806621 | Inversion |
| Chr8 | 39289062 | 39289199 | chr8 | 8524531 | 8524668 | Intra-translocation |
| Chr8 | 39389710 | 39389475 | chr3 | 13164105 | 13164340 | Inversion |
| Chr8 | 39391911 | 39392025 | chr7 | 35455645 | 35455758 | Inter-translocation |
| Chr8 | 39397170 | 39394852 | chr8 | 30410710 | 30413021 | Inversion |
| Chr8 | 39410724 | 39411393 | chr8 | 39234261 | 39234939 | Intra-translocation |
| Chr8 | 39411262 | 39411462 | chr8 | 39234939 | 39235139 | Intra-translocation |
| Chr8 | 39411599 | 39416930 | chr8 | 39235140 | 39240457 | Intra-translocation |
| Chr8 | 39417107 | 39427765 | chr8 | 39240635 | 39251294 | Intra-translocation |
| Chr8 | 39557436 | 39557163 | chr2 | 3828788 | 3829061 | Inversion |
| Chr8 | 39606813 | 39607810 | chr8 | 35744083 | 35745063 | Intra-translocation |
| Chr8 | 39670979 | 39670502 | chr8 | 2653574 | 2654024 | Inversion |
| Chr8 | 39818717 | 39819891 | chr8 | 14615720 | 14616909 | Intra-translocation |
| Chr8 | 39856133 | 39855893 | chr8 | 25420205 | 25420445 | Inversion |
| Chr8 | 39876777 | 39876712 | chr8 | 34945957 | 34946022 | Inversion |
| Chr8 | 39879998 | 39879892 | chr8 | 40251650 | 40251757 | Inversion |
| Chr8 | 39887483 | 39888871 | chr3 | 22862527 | 22863917 | Inter-translocation |
| Chr8 | 39898936 | 39898791 | chr8 | 29312237 | 29312382 | Inversion |
| Chr8 | 39905039 | 39904888 | chr2 | 5406639 | 5406790 | Inversion |
| Chr8 | 39905045 | 39905492 | chr8 | 30267422 | 30267866 | Intra-translocation |
| Chr8 | 39928191 | 39929760 | chr8 | 36809069 | 36810638 | Intra-translocation |
| Chr8 | 39932937 | 39931966 | chr8 | 2837475 | 2838436 | Inversion |
| Chr8 | 39937564 | 39932930 | chr8 | 2832485 | 2837128 | Inversion |
| Chr8 | 39960602 | 39961729 | chr8 | 2823812 | 2824953 | Intra-translocation |
| Chr8 | 39961727 | 39965596 | chr8 | 2825304 | 2829214 | Intra-translocation |
| Chr8 | 39965605 | 39965881 | chr8 | 2830557 | 2830839 | Intra-translocation |
| Chr8 | 40010503 | 40012380 | chr8 | 30052521 | 30054400 | Intra-translocation |
| Chr8 | 40044750 | 40044945 | chr8 | 36811549 | 36811744 | Intra-translocation |
| Chr8 | 40047735 | 40047296 | chr8 | 34951480 | 34951914 | Inversion |
| Chr8 | 40064426 | 40063414 | chr8 | 38012997 | 38014041 | Inversion |
| Chr8 | 40065519 | 40063496 | chr8 | 38001791 | 38003790 | Inversion |
| Chr8 | 40068861 | 40068287 | chr8 | 38001214 | 38001796 | Inversion |
| Chr8 | 40069025 | 40069753 | chr8 | 36808923 | 36809651 | Intra-translocation |
| Chr8 | 40108516 | 40108817 | chr8 | 25905742 | 25906042 | Intra-translocation |
| Chr8 | 40108812 | 40108276 | chr9 | 532855 | 533360 | Inversion |
| Chr8 | 40201628 | 40204201 | chr8 | 39826008 | 39828580 | Intra-translocation |
| Chr8 | 40221787 | 40223287 | chr9 | 34282591 | 34284014 | Inter-translocation |
| Chr8 | 40244735 | 40242508 | chr3 | 7116485 | 7118713 | Inversion |
| Chr8 | 40300053 | 40296020 | chr8 | 40155664 | 40159738 | Inversion |
| Chr8 | 40322540 | 40335841 | chr8 | 39904351 | 39917639 | Intra-translocation |
| Chr8 | 40335831 | 40337833 | chr8 | 39926712 | 39928713 | Intra-translocation |
| Chr8 | 40356808 | 40341169 | chr8 | 39989604 | 40005227 | Inversion |
| Chr8 | 40372058 | 40371077 | chr8 | 39975852 | 39976832 | Inversion |
| Chr8 | 40386473 | 40372054 | chr8 | 39948101 | 39962513 | Inversion |
| Chr8 | 40387514 | 40386560 | chr8 | 39946532 | 39947488 | Inversion |
| Chr8 | 40400858 | 40399169 | chr8 | 39938002 | 39939691 | Inversion |
| Chr8 | 40417170 | 40410740 | chr8 | 39895650 | 39902062 | Inversion |
| Chr8 | 40430384 | 40417167 | chr8 | 39882473 | 39895689 | Inversion |
| Chr8 | 40446906 | 40446186 | chr8 | 39901764 | 39902485 | Inversion |
| Chr8 | 40447992 | 40456141 | chr8 | 3044831 | 3052971 | Intra-translocation |
| Chr8 | 40475169 | 40479407 | chr4 | 25120885 | 25125117 | Inter-translocation |
| Chr8 | 40479947 | 40476875 | chr9 | 30876145 | 30879217 | Inversion |
| Chr8 | 40480620 | 40483468 | chr3 | 13316984 | 13319839 | Inter-translocation |
| Chr8 | 40484082 | 40480622 | chr8 | 22388438 | 22391899 | Inversion |
| Chr8 | 40487503 | 40488453 | chr8 | 11715177 | 11716116 | Intra-translocation |
| Chr8 | 40503901 | 40503281 | chr8 | 5399675 | 5400295 | Inversion |
| Chr8 | 40537172 | 40537289 | chr2 | 41521443 | 41521560 | Inter-translocation |
| Chr8 | 40588054 | 40587537 | chr8 | 12431539 | 12432057 | Inversion |
| Chr8 | 40748538 | 40748333 | chr8 | 36806849 | 36807054 | Inversion |
| Chr8 | 40852159 | 40852398 | chr3 | 44525780 | 44526020 | Inter-translocation |
| Chr8 | 40954317 | 40954121 | chr8 | 5765846 | 5766042 | Inversion |
| Chr8 | 41152726 | 41153241 | chr8 | 39218397 | 39218909 | Intra-translocation |
| Chr8 | 41161051 | 41160717 | chr8 | 38820902 | 38821233 | Inversion |
| Chr8 | 41161057 | 41161208 | chr8 | 39227156 | 39227307 | Intra-translocation |
| Chr8 | 41161400 | 41161657 | chr8 | 39227725 | 39227982 | Intra-translocation |
| Chr8 | 41167835 | 41168005 | chr5 | 15434979 | 15435149 | Inter-translocation |
| Chr8 | 41186308 | 41184839 | chr8 | 30412629 | 30414127 | Inversion |
| Chr9 | 247386 | 247225 | chr2 | 41055373 | 41055534 | Inversion |
| Chr9 | 401118 | 402364 | chr3 | 50209496 | 50210696 | Inter-translocation |
| Chr9 | 401344 | 402380 | chr3 | 50020244 | 50021269 | Inter-translocation |
| Chr9 | 402383 | 404614 | chr3 | 50021772 | 50023997 | Inter-translocation |
| Chr9 | 405063 | 407533 | chr3 | 50212921 | 50215373 | Inter-translocation |
| Chr9 | 548963 | 548880 | chr1 | 14625855 | 14625939 | Inversion |
| Chr9 | 569566 | 570251 | chr3 | 13271190 | 13271890 | Inter-translocation |
| Chr9 | 590199 | 590914 | chr9 | 599720 | 600431 | Intra-translocation |
| Chr9 | 591253 | 591063 | chr6 | 2466322 | 2466504 | Inversion |
| Chr9 | 607979 | 608155 | chr2 | 2570354 | 2570530 | Inter-translocation |
| Chr9 | 705523 | 705784 | chr6 | 35725887 | 35726146 | Inter-translocation |
| Chr9 | 706283 | 706420 | chr6 | 35726148 | 35726285 | Inter-translocation |
| Chr9 | 1988506 | 1988159 | chr7 | 27853065 | 27853412 | Inversion |
| Chr9 | 2045590 | 2045307 | chr3 | 24754125 | 24754408 | Inversion |
| Chr9 | 2048032 | 2045968 | chr9 | 50517280 | 50519344 | Inversion |
| Chr9 | 2057948 | 2057743 | chr3 | 40622180 | 40622385 | Inversion |
| Chr9 | 2063721 | 2063440 | chr2 | 45838037 | 45838318 | Inversion |
| Chr9 | 2317390 | 2317711 | chr3 | 6923239 | 6923560 | Inter-translocation |
| Chr9 | 2358892 | 2371434 | chr9 | 3589903 | 3602424 | Intra-translocation |
| Chr9 | 2835207 | 2828531 | chr9 | 2865074 | 2871749 | Inversion |
| Chr9 | 2868344 | 2835248 | chr9 | 2831518 | 2864613 | Inversion |
| Chr9 | 2881356 | 2868508 | chr9 | 2818451 | 2831286 | Inversion |
| Chr9 | 2906211 | 2889405 | chr9 | 2791643 | 2808450 | Inversion |
| Chr9 | 3211747 | 3213744 | chr9 | 3195172 | 3197169 | Intra-translocation |
| Chr9 | 3215731 | 3215523 | chr9 | 56964569 | 56964782 | Inversion |
| Chr9 | 3276173 | 3276446 | chr1 | 26479911 | 26480184 | Inter-translocation |
| Chr9 | 3791255 | 3793104 | chr9 | 3785024 | 3786874 | Intra-translocation |
| Chr9 | 5602247 | 5602510 | chr8 | 6887279 | 6887542 | Inter-translocation |
| Chr9 | 5621641 | 5620794 | chr9 | 12178894 | 12179741 | Inversion |
| Chr9 | 7189670 | 7190284 | chr8 | 12756036 | 12756650 | Inter-translocation |
| Chr9 | 7470967 | 7470624 | chr9 | 19008003 | 19008336 | Inversion |
| Chr9 | 8152361 | 8152613 | chr2 | 42234336 | 42234589 | Inter-translocation |
| Chr9 | 8693427 | 8693628 | chr9 | 3268134 | 3268335 | Intra-translocation |
| Chr9 | 8812205 | 8812348 | chr9 | 35728540 | 35728683 | Intra-translocation |
| Chr9 | 9684936 | 9684088 | chr6 | 34651361 | 34652216 | Inversion |
| Chr9 | 9921125 | 9920649 | chr2 | 20267982 | 20268461 | Inversion |
| Chr9 | 10753573 | 10753920 | chr5 | 25775219 | 25775566 | Inter-translocation |
| Chr9 | 10848600 | 10848320 | chr1 | 241990 | 242270 | Inversion |
| Chr9 | 10855136 | 10855484 | chr7 | 35043016 | 35043364 | Inter-translocation |
| Chr9 | 10855337 | 10855135 | chr9 | 52734508 | 52734710 | Inversion |
| Chr9 | 11272868 | 11267202 | chr2 | 9332359 | 9338020 | Inversion |
| Chr9 | 11635363 | 11638466 | chr8 | 25250156 | 25253259 | Inter-translocation |
| Chr9 | 11638467 | 11640114 | chr8 | 25253568 | 25255214 | Inter-translocation |
| Chr9 | 11972462 | 11972109 | chr9 | 22906452 | 22906805 | Inversion |
| Chr9 | 13121514 | 13134692 | chr9 | 8012514 | 8025687 | Intra-translocation |
| Chr9 | 13313716 | 13296015 | chr9 | 13282953 | 13300655 | Inversion |
| Chr9 | 13494588 | 13494852 | chr3 | 47699501 | 47699765 | Inter-translocation |
| Chr9 | 13976065 | 13976570 | chr4 | 5415670 | 5416174 | Inter-translocation |
| Chr9 | 14800365 | 14800546 | chr4 | 12083514 | 12083695 | Inter-translocation |
| Chr9 | 15539179 | 15534218 | chr9 | 23700442 | 23705395 | Inversion |
| Chr9 | 16222985 | 16222630 | chr7 | 26554088 | 26554443 | Inversion |
| Chr9 | 16273881 | 16273633 | chr5 | 15638278 | 15638525 | Inversion |
| Chr9 | 16546422 | 16546135 | chr8 | 8152483 | 8152770 | Inversion |
| Chr9 | 16588702 | 16589060 | chr2 | 46213706 | 46214064 | Inter-translocation |
| Chr9 | 16775561 | 16774978 | chr8 | 19532488 | 19533071 | Inversion |
| Chr9 | 17131893 | 17131774 | chr4 | 8930929 | 8931048 | Inversion |
| Chr9 | 17141420 | 17140208 | chr3 | 971698 | 972925 | Inversion |
| Chr9 | 17147141 | 17141986 | chr7 | 4467778 | 4472917 | Inversion |
| Chr9 | 17172245 | 17147453 | chr7 | 4442699 | 4467493 | Inversion |
| Chr9 | 17219430 | 17219235 | chr3 | 9582269 | 9582463 | Inversion |
| Chr9 | 17767227 | 17767590 | chr8 | 25587230 | 25587593 | Inter-translocation |
| Chr9 | 17984457 | 17984800 | chr8 | 38793935 | 38794277 | Inter-translocation |
| Chr9 | 18105471 | 18105360 | chr1 | 26101778 | 26101889 | Inversion |
| Chr9 | 18140820 | 18141520 | chr9 | 23256879 | 23257599 | Intra-translocation |
| Chr9 | 18141518 | 18140819 | chr6 | 35649979 | 35650677 | Inversion |
| Chr9 | 18156126 | 18158326 | chr8 | 13937281 | 13939481 | Inter-translocation |
| Chr9 | 18199905 | 18200643 | chr4 | 27804028 | 27804766 | Inter-translocation |
| Chr9 | 18288398 | 18287246 | chr9 | 16225219 | 16226375 | Inversion |
| Chr9 | 18497334 | 18496312 | chr3 | 47284383 | 47285401 | Inversion |
| Chr9 | 19014654 | 19015582 | chr9 | 52405329 | 52406258 | Intra-translocation |
| Chr9 | 19050633 | 19044920 | chr9 | 19100160 | 19105849 | Inversion |
| Chr9 | 19050938 | 19050679 | chr9 | 19094625 | 19094883 | Inversion |
| Chr9 | 19052082 | 19051107 | chr9 | 19093481 | 19094456 | Inversion |
| Chr9 | 19054514 | 19052152 | chr9 | 19090702 | 19093065 | Inversion |
| Chr9 | 19057952 | 19054857 | chr9 | 19087683 | 19090676 | Inversion |
| Chr9 | 19059467 | 19057937 | chr9 | 19085786 | 19087257 | Inversion |
| Chr9 | 19062428 | 19059463 | chr9 | 19082438 | 19085435 | Inversion |
| Chr9 | 19077048 | 19062702 | chr9 | 19068031 | 19082441 | Inversion |
| Chr9 | 19093314 | 19077051 | chr9 | 19047108 | 19063262 | Inversion |
| Chr9 | 19098427 | 19093660 | chr9 | 19042384 | 19047111 | Inversion |
| Chr9 | 19100388 | 19098427 | chr9 | 19040164 | 19042115 | Inversion |
| Chr9 | 19116557 | 19100798 | chr9 | 19024157 | 19039967 | Inversion |
| Chr9 | 19135562 | 19119944 | chr9 | 19008533 | 19024132 | Inversion |
| Chr9 | 19139486 | 19135560 | chr9 | 19004089 | 19008003 | Inversion |
| Chr9 | 19141793 | 19139760 | chr9 | 19002057 | 19004092 | Inversion |
| Chr9 | 19142114 | 19141789 | chr9 | 19001518 | 19001843 | Inversion |
| Chr9 | 19153352 | 19142389 | chr9 | 18990533 | 19001520 | Inversion |
| Chr9 | 19171217 | 19153442 | chr9 | 18972812 | 18990530 | Inversion |
| Chr9 | 19176033 | 19171278 | chr9 | 18967795 | 18972575 | Inversion |
| Chr9 | 19179494 | 19178229 | chr7 | 14632399 | 14633669 | Inversion |
| Chr9 | 19241561 | 19238528 | chr5 | 14958922 | 14961955 | Inversion |
| Chr9 | 19312283 | 19312443 | chr8 | 23162129 | 23162289 | Inter-translocation |
| Chr9 | 19590405 | 19586824 | chr9 | 22467613 | 22471207 | Inversion |
| Chr9 | 19595970 | 19590756 | chr9 | 22462097 | 22467309 | Inversion |
| Chr9 | 19596243 | 19596167 | chr9 | 22461883 | 22461959 | Inversion |
| Chr9 | 19596725 | 19596239 | chr9 | 22461124 | 22461610 | Inversion |
| Chr9 | 19647512 | 19596739 | chr9 | 22410186 | 22461023 | Inversion |
| Chr9 | 19665869 | 19647330 | chr9 | 22391886 | 22410368 | Inversion |
| Chr9 | 19700247 | 19665932 | chr9 | 22353762 | 22388030 | Inversion |
| Chr9 | 19731273 | 19702104 | chr9 | 22322357 | 22351522 | Inversion |
| Chr9 | 19745469 | 19731523 | chr9 | 22306157 | 22320106 | Inversion |
| Chr9 | 19777334 | 19746371 | chr9 | 22275043 | 22306054 | Inversion |
| Chr9 | 19818392 | 19777986 | chr9 | 22234630 | 22275043 | Inversion |
| Chr9 | 19818749 | 19818393 | chr9 | 56893267 | 56893623 | Inversion |
| Chr9 | 19819055 | 19818747 | chr9 | 22234325 | 22234633 | Inversion |
| Chr9 | 19841924 | 19819051 | chr9 | 22210560 | 22233413 | Inversion |
| Chr9 | 19846425 | 19842272 | chr9 | 22206368 | 22210526 | Inversion |
| Chr9 | 19858270 | 19852016 | chr9 | 22195306 | 22201580 | Inversion |
| Chr9 | 19860203 | 19858265 | chr9 | 22193185 | 22195044 | Inversion |
| Chr9 | 19861414 | 19860666 | chr9 | 22192404 | 22193179 | Inversion |
| Chr9 | 19867902 | 19861412 | chr9 | 22185592 | 22192054 | Inversion |
| Chr9 | 19868056 | 19867901 | chr9 | 22185090 | 22185245 | Inversion |
| Chr9 | 19883188 | 19868053 | chr9 | 22169218 | 22184294 | Inversion |
| Chr9 | 19887039 | 19883190 | chr9 | 22165280 | 22169128 | Inversion |
| Chr9 | 19889270 | 19887034 | chr9 | 22161880 | 22164117 | Inversion |
| Chr9 | 19908209 | 19906173 | chr8 | 13867493 | 13869531 | Inversion |
| Chr9 | 19913124 | 19909390 | chr9 | 22158148 | 22161885 | Inversion |
| Chr9 | 19919956 | 19913435 | chr9 | 22151615 | 22158148 | Inversion |
| Chr9 | 19926449 | 19919950 | chr9 | 22131342 | 22137795 | Inversion |
| Chr9 | 19955614 | 19926581 | chr9 | 22101800 | 22130928 | Inversion |
| Chr9 | 19956516 | 19955613 | chr9 | 22100849 | 22101762 | Inversion |
| Chr9 | 19977352 | 19956512 | chr9 | 22079791 | 22100698 | Inversion |
| Chr9 | 19978882 | 19977342 | chr9 | 22078216 | 22079749 | Inversion |
| Chr9 | 19979610 | 19978878 | chr9 | 22072499 | 22073215 | Inversion |
| Chr9 | 19979655 | 19979528 | chr9 | 22070892 | 22071015 | Inversion |
| Chr9 | 19980867 | 19979654 | chr4 | 26937858 | 26939066 | Inversion |
| Chr9 | 19981309 | 19984469 | chr8 | 4269233 | 4272410 | Inter-translocation |
| Chr9 | 19985556 | 19989090 | chr8 | 29632455 | 29635973 | Inter-translocation |
| Chr9 | 19987323 | 19982361 | chr8 | 39708104 | 39713079 | Inversion |
| Chr9 | 19993285 | 19993473 | chr8 | 5390531 | 5390719 | Inter-translocation |
| Chr9 | 19998215 | 19993472 | chr9 | 22066046 | 22070895 | Inversion |
| Chr9 | 20000238 | 19998337 | chr9 | 22064155 | 22066061 | Inversion |
| Chr9 | 20005974 | 20001423 | chr9 | 22059633 | 22064159 | Inversion |
| Chr9 | 20010563 | 20005974 | chr9 | 22055205 | 22059795 | Inversion |
| Chr9 | 20013551 | 20010872 | chr9 | 22052221 | 22054897 | Inversion |
| Chr9 | 20013951 | 20013549 | chr9 | 22051524 | 22051926 | Inversion |
| Chr9 | 20019232 | 20014776 | chr9 | 22047084 | 22051536 | Inversion |
| Chr9 | 20040836 | 20038014 | chr9 | 22044027 | 22046848 | Inversion |
| Chr9 | 20088297 | 20071546 | chr9 | 22025118 | 22041832 | Inversion |
| Chr9 | 20091214 | 20088298 | chr9 | 22022300 | 22025216 | Inversion |
| Chr9 | 20115375 | 20091576 | chr9 | 21998483 | 22022302 | Inversion |
| Chr9 | 20123093 | 20119603 | chr9 | 21989221 | 21992707 | Inversion |
| Chr9 | 20135698 | 20123369 | chr9 | 21976841 | 21989225 | Inversion |
| Chr9 | 20139790 | 20137028 | chr9 | 21974078 | 21976837 | Inversion |
| Chr9 | 20142165 | 20139786 | chr9 | 21963672 | 21966047 | Inversion |
| Chr9 | 20145114 | 20142326 | chr9 | 21960907 | 21963672 | Inversion |
| Chr9 | 20146281 | 20145126 | chr9 | 21959613 | 21960768 | Inversion |
| Chr9 | 20146920 | 20146371 | chr9 | 21959062 | 21959612 | Inversion |
| Chr9 | 20150910 | 20147092 | chr9 | 21955229 | 21959062 | Inversion |
| Chr9 | 20151563 | 20150953 | chr9 | 21954260 | 21954882 | Inversion |
| Chr9 | 20155302 | 20151563 | chr9 | 21950391 | 21954155 | Inversion |
| Chr9 | 20156151 | 20160689 | chr9 | 23188999 | 23193532 | Intra-translocation |
| Chr9 | 20182509 | 20180246 | chr9 | 21948149 | 21950387 | Inversion |
| Chr9 | 20182543 | 20182264 | chr9 | 21947881 | 21948148 | Inversion |
| Chr9 | 20184981 | 20184559 | chr9 | 21947464 | 21947885 | Inversion |
| Chr9 | 20187428 | 20184979 | chr9 | 21944527 | 21946979 | Inversion |
| Chr9 | 20190677 | 20187699 | chr9 | 21941569 | 21944522 | Inversion |
| Chr9 | 20219529 | 20201629 | chr9 | 21911837 | 21929754 | Inversion |
| Chr9 | 20243431 | 20219529 | chr9 | 21888704 | 21912585 | Inversion |
| Chr9 | 20249179 | 20243448 | chr9 | 21882771 | 21888500 | Inversion |
| Chr9 | 20250760 | 20249911 | chr9 | 21881913 | 21882762 | Inversion |
| Chr9 | 20257431 | 20251107 | chr9 | 21875613 | 21881911 | Inversion |
| Chr9 | 20266732 | 20258558 | chr9 | 21867447 | 21875525 | Inversion |
| Chr9 | 20267478 | 20267757 | chr2 | 22699509 | 22699788 | Inter-translocation |
| Chr9 | 20267480 | 20266730 | chr9 | 21866373 | 21867125 | Inversion |
| Chr9 | 20281457 | 20267755 | chr9 | 21852759 | 21866375 | Inversion |
| Chr9 | 20288704 | 20281572 | chr9 | 21845652 | 21852759 | Inversion |
| Chr9 | 20289356 | 20289221 | chr9 | 21845518 | 21845653 | Inversion |
| Chr9 | 20290653 | 20290914 | chr9 | 31878902 | 31879167 | Intra-translocation |
| Chr9 | 20290659 | 20289368 | chr9 | 21843658 | 21845001 | Inversion |
| Chr9 | 20293793 | 20290910 | chr9 | 21840796 | 21843664 | Inversion |
| Chr9 | 20294148 | 20293791 | chr9 | 21840163 | 21840520 | Inversion |
| Chr9 | 20320862 | 20294146 | chr9 | 21812688 | 21839539 | Inversion |
| Chr9 | 20321091 | 20320998 | chr9 | 21812575 | 21812668 | Inversion |
| Chr9 | 20333223 | 20321135 | chr9 | 21800412 | 21812576 | Inversion |
| Chr9 | 20335188 | 20335485 | chr8 | 18305251 | 18305548 | Inter-translocation |
| Chr9 | 20350094 | 20347670 | chr9 | 21796311 | 21798743 | Inversion |
| Chr9 | 20355579 | 20350097 | chr9 | 21765345 | 21770811 | Inversion |
| Chr9 | 20357753 | 20355926 | chr9 | 21763519 | 21765346 | Inversion |
| Chr9 | 20362192 | 20357743 | chr9 | 21758758 | 21763171 | Inversion |
| Chr9 | 20364855 | 20366266 | chr1 | 25946178 | 25947586 | Inter-translocation |
| Chr9 | 20366939 | 20366263 | chr9 | 21758101 | 21758759 | Inversion |
| Chr9 | 20374978 | 20366996 | chr9 | 21748654 | 21756722 | Inversion |
| Chr9 | 20396935 | 20374970 | chr9 | 21725935 | 21747890 | Inversion |
| Chr9 | 20412336 | 20397194 | chr9 | 21710342 | 21725487 | Inversion |
| Chr9 | 20415155 | 20412540 | chr9 | 21707698 | 21710312 | Inversion |
| Chr9 | 20417185 | 20416215 | chr9 | 21706732 | 21707703 | Inversion |
| Chr9 | 20419179 | 20417183 | chr9 | 21704411 | 21706363 | Inversion |
| Chr9 | 20420725 | 20419462 | chr9 | 21703226 | 21704408 | Inversion |
| Chr9 | 20423914 | 20423632 | chr9 | 21702838 | 21703122 | Inversion |
| Chr9 | 20441459 | 20423911 | chr9 | 21685292 | 21702564 | Inversion |
| Chr9 | 20463697 | 20462748 | chr9 | 21684318 | 21685295 | Inversion |
| Chr9 | 20490150 | 20478225 | chr9 | 21672431 | 21684322 | Inversion |
| Chr9 | 20491384 | 20489724 | chr9 | 21665790 | 21667441 | Inversion |
| Chr9 | 20494622 | 20491384 | chr9 | 21662374 | 21665625 | Inversion |
| Chr9 | 20494728 | 20494632 | chr9 | 21662176 | 21662272 | Inversion |
| Chr9 | 20514741 | 20494976 | chr9 | 21642331 | 21662133 | Inversion |
| Chr9 | 20515168 | 20515015 | chr9 | 21642181 | 21642334 | Inversion |
| Chr9 | 20530669 | 20528968 | chr9 | 21631273 | 21632968 | Inversion |
| Chr9 | 20547351 | 20531930 | chr9 | 21605309 | 21620715 | Inversion |
| Chr9 | 20547846 | 20547343 | chr9 | 21600394 | 21600897 | Inversion |
| Chr9 | 20552725 | 20548079 | chr9 | 21595751 | 21600397 | Inversion |
| Chr9 | 20575351 | 20552722 | chr9 | 21569134 | 21591796 | Inversion |
| Chr9 | 20577161 | 20575351 | chr9 | 21567183 | 21569003 | Inversion |
| Chr9 | 20578635 | 20577425 | chr9 | 21565998 | 21567187 | Inversion |
| Chr9 | 20589120 | 20578630 | chr9 | 21555556 | 21565966 | Inversion |
| Chr9 | 20596239 | 20592269 | chr9 | 21551583 | 21555564 | Inversion |
| Chr9 | 20603024 | 20596401 | chr9 | 21544809 | 21551434 | Inversion |
| Chr9 | 20607926 | 20603022 | chr9 | 21539368 | 21544257 | Inversion |
| Chr9 | 20608570 | 20607933 | chr9 | 21537876 | 21538515 | Inversion |
| Chr9 | 20608828 | 20608677 | chr9 | 21537626 | 21537777 | Inversion |
| Chr9 | 20615262 | 20612069 | chr9 | 21534439 | 21537636 | Inversion |
| Chr9 | 20617618 | 20615259 | chr9 | 21528740 | 21531119 | Inversion |
| Chr9 | 20630666 | 20617616 | chr9 | 21515435 | 21528393 | Inversion |
| Chr9 | 20637862 | 20631051 | chr9 | 21508642 | 21515438 | Inversion |
| Chr9 | 20655923 | 20641084 | chr9 | 21491794 | 21506699 | Inversion |
| Chr9 | 20658896 | 20657047 | chr9 | 21489948 | 21491803 | Inversion |
| Chr9 | 20668457 | 20664122 | chr9 | 21485618 | 21489952 | Inversion |
| Chr9 | 20679901 | 20668455 | chr9 | 21473769 | 21485272 | Inversion |
| Chr9 | 20682815 | 20680875 | chr9 | 21471790 | 21473718 | Inversion |
| Chr9 | 20688313 | 20682812 | chr9 | 21466269 | 21471746 | Inversion |
| Chr9 | 20693599 | 20688949 | chr9 | 21461680 | 21466271 | Inversion |
| Chr9 | 20697855 | 20693594 | chr9 | 21444346 | 21448619 | Inversion |
| Chr9 | 20705861 | 20704857 | chr9 | 21443347 | 21444351 | Inversion |
| Chr9 | 20737491 | 20705863 | chr9 | 21411386 | 21443119 | Inversion |
| Chr9 | 20739099 | 20737485 | chr9 | 21409430 | 21411041 | Inversion |
| Chr9 | 20740030 | 20739374 | chr9 | 21408770 | 21409411 | Inversion |
| Chr9 | 20741592 | 20740041 | chr9 | 21407001 | 21408554 | Inversion |
| Chr9 | 20746457 | 20744855 | chr9 | 21405373 | 21406963 | Inversion |
| Chr9 | 20758208 | 20746568 | chr9 | 21393583 | 21405373 | Inversion |
| Chr9 | 20764326 | 20758552 | chr9 | 21387760 | 21393586 | Inversion |
| Chr9 | 20783027 | 20782902 | chr9 | 29911323 | 29911448 | Inversion |
| Chr9 | 20787806 | 20785827 | chr9 | 21385706 | 21387765 | Inversion |
| Chr9 | 20789430 | 20787800 | chr9 | 21380721 | 21382355 | Inversion |
| Chr9 | 20790803 | 20789428 | chr9 | 21378987 | 21380362 | Inversion |
| Chr9 | 20791420 | 20790804 | chr9 | 32772909 | 32773525 | Inversion |
| Chr9 | 20805890 | 20791821 | chr9 | 21364915 | 21378990 | Inversion |
| Chr9 | 20834318 | 20806006 | chr9 | 21336113 | 21364426 | Inversion |
| Chr9 | 20874064 | 20834514 | chr9 | 21295705 | 21335295 | Inversion |
| Chr9 | 20939396 | 20874127 | chr9 | 21230099 | 21295373 | Inversion |
| Chr9 | 20944000 | 20939410 | chr9 | 21225536 | 21230126 | Inversion |
| Chr9 | 20956855 | 20943989 | chr9 | 21211809 | 21224677 | Inversion |
| Chr9 | 20984933 | 20968351 | chr9 | 21195203 | 21211816 | Inversion |
| Chr9 | 20987038 | 20985585 | chr9 | 21193624 | 21195082 | Inversion |
| Chr9 | 21060989 | 20987844 | chr9 | 21118436 | 21191628 | Inversion |
| Chr9 | 21090272 | 21061246 | chr9 | 21088619 | 21117720 | Inversion |
| Chr9 | 21092338 | 21090267 | chr2 | 47920249 | 47922320 | Inversion |
| Chr9 | 21095106 | 21092636 | chr2 | 47917678 | 47920148 | Inversion |
| Chr9 | 21099143 | 21095104 | chr9 | 21084583 | 21088622 | Inversion |
| Chr9 | 21117975 | 21099390 | chr9 | 21065143 | 21083726 | Inversion |
| Chr9 | 21124107 | 21118207 | chr9 | 21058987 | 21064885 | Inversion |
| Chr9 | 21136424 | 21124861 | chr9 | 21046492 | 21058055 | Inversion |
| Chr9 | 21148948 | 21136851 | chr9 | 21034259 | 21046355 | Inversion |
| Chr9 | 21247750 | 21149023 | chr9 | 20933451 | 21032200 | Inversion |
| Chr9 | 21251140 | 21249277 | chr9 | 20931595 | 20933458 | Inversion |
| Chr9 | 21251159 | 21251487 | chr3 | 46553902 | 46554229 | Inter-translocation |
| Chr9 | 21252983 | 21251487 | chr9 | 20930102 | 20931598 | Inversion |
| Chr9 | 21283636 | 21253330 | chr9 | 20899740 | 20930104 | Inversion |
| Chr9 | 21283702 | 21283638 | chr9 | 20899299 | 20899363 | Inversion |
| Chr9 | 21287220 | 21284052 | chr9 | 20896134 | 20899301 | Inversion |
| Chr9 | 21349405 | 21287494 | chr9 | 20834230 | 20896137 | Inversion |
| Chr9 | 21350160 | 21351308 | chr1 | 25269160 | 25270307 | Inter-translocation |
| Chr9 | 21358894 | 21350759 | chr9 | 20825798 | 20833930 | Inversion |
| Chr9 | 21505333 | 21359079 | chr9 | 20679190 | 20825481 | Inversion |
| Chr9 | 21516495 | 21505425 | chr9 | 20667760 | 20678830 | Inversion |
| Chr9 | 21576168 | 21517635 | chr9 | 20612629 | 20671192 | Inversion |
| Chr9 | 21676473 | 21576224 | chr9 | 20512154 | 20612516 | Inversion |
| Chr9 | 21681160 | 21676470 | chr9 | 20506560 | 20511241 | Inversion |
| Chr9 | 21706815 | 21683498 | chr9 | 20483235 | 20506560 | Inversion |
| Chr9 | 21716252 | 21716604 | chr2 | 48030293 | 48030645 | Inter-translocation |
| Chr9 | 21716256 | 21706802 | chr9 | 20473731 | 20483183 | Inversion |
| Chr9 | 21722785 | 21716601 | chr9 | 20467602 | 20473734 | Inversion |
| Chr9 | 21723816 | 21723514 | chr2 | 3921009 | 3921311 | Inversion |
| Chr9 | 21747337 | 21724646 | chr9 | 20445346 | 20468039 | Inversion |
| Chr9 | 21759541 | 21747699 | chr9 | 20433669 | 20445349 | Inversion |
| Chr9 | 21766248 | 21759882 | chr9 | 20427263 | 20433672 | Inversion |
| Chr9 | 21776081 | 21766500 | chr9 | 20417403 | 20426927 | Inversion |
| Chr9 | 21776723 | 21776081 | chr9 | 20416053 | 20416696 | Inversion |
| Chr9 | 21784977 | 21776785 | chr9 | 20407555 | 20415756 | Inversion |
| Chr9 | 21806975 | 21785291 | chr9 | 20385859 | 20407557 | Inversion |
| Chr9 | 21817128 | 21806694 | chr9 | 20375233 | 20385654 | Inversion |
| Chr9 | 21843056 | 21817126 | chr9 | 20330002 | 20355919 | Inversion |
| Chr9 | 21846015 | 21844231 | chr9 | 20328223 | 20330007 | Inversion |
| Chr9 | 21859988 | 21846012 | chr9 | 20313441 | 20327560 | Inversion |
| Chr9 | 21877398 | 21873135 | chr9 | 20309031 | 20313445 | Inversion |
| Chr9 | 21878698 | 21877397 | chr9 | 20307796 | 20309083 | Inversion |
| Chr9 | 21879035 | 21878870 | chr9 | 20307618 | 20307783 | Inversion |
| Chr9 | 21888563 | 21880735 | chr9 | 20299567 | 20307515 | Inversion |
| Chr9 | 21892476 | 21888685 | chr9 | 20295794 | 20299567 | Inversion |
| Chr9 | 21902010 | 21892474 | chr9 | 20286193 | 20295674 | Inversion |
| Chr9 | 21903498 | 21902331 | chr9 | 20285026 | 20286193 | Inversion |
| Chr9 | 21907772 | 21903496 | chr9 | 20280672 | 20284905 | Inversion |
| Chr9 | 21908919 | 21907782 | chr9 | 20279286 | 20280425 | Inversion |
| Chr9 | 21942551 | 21909100 | chr9 | 20245814 | 20279300 | Inversion |
| Chr9 | 21948001 | 21944588 | chr9 | 20242394 | 20245812 | Inversion |
| Chr9 | 21948014 | 21948082 | chr8 | 8685914 | 8685983 | Inter-translocation |
| Chr9 | 21953950 | 21948597 | chr9 | 20237065 | 20242399 | Inversion |
| Chr9 | 21954756 | 21953947 | chr9 | 20235888 | 20236697 | Inversion |
| Chr9 | 21955254 | 21955051 | chr9 | 20235692 | 20235895 | Inversion |
| Chr9 | 21955762 | 21955246 | chr9 | 20225132 | 20225646 | Inversion |
| Chr9 | 21959169 | 21955784 | chr9 | 20221354 | 20224737 | Inversion |
| Chr9 | 21964722 | 21959177 | chr9 | 20215662 | 20221219 | Inversion |
| Chr9 | 21967719 | 21964716 | chr9 | 20211636 | 20214607 | Inversion |
| Chr9 | 21985013 | 21967702 | chr9 | 20191647 | 20208969 | Inversion |
| Chr9 | 22001669 | 21993176 | chr9 | 20183160 | 20191652 | Inversion |
| Chr9 | 22008639 | 22001688 | chr9 | 20176056 | 20183006 | Inversion |
| Chr9 | 22014947 | 22008636 | chr9 | 20167592 | 20173903 | Inversion |
| Chr9 | 22032314 | 22015062 | chr9 | 20150426 | 20167587 | Inversion |
| Chr9 | 22033996 | 22032336 | chr9 | 20148607 | 20150289 | Inversion |
| Chr9 | 22035327 | 22033984 | chr9 | 20147214 | 20148554 | Inversion |
| Chr9 | 22036897 | 22035304 | chr9 | 20145560 | 20147129 | Inversion |
| Chr9 | 22081576 | 22037246 | chr9 | 20101288 | 20145560 | Inversion |
| Chr9 | 22086527 | 22081635 | chr9 | 20090271 | 20095180 | Inversion |
| Chr9 | 22092940 | 22086488 | chr9 | 20083173 | 20089558 | Inversion |
| Chr9 | 22094454 | 22092938 | chr9 | 20081438 | 20082955 | Inversion |
| Chr9 | 22101240 | 22100170 | chr9 | 20080203 | 20081274 | Inversion |
| Chr9 | 22157029 | 22101307 | chr9 | 20024256 | 20080243 | Inversion |
| Chr9 | 22168639 | 22169507 | chr8 | 13448207 | 13449122 | Inter-translocation |
| Chr9 | 22168641 | 22157130 | chr9 | 20012731 | 20024254 | Inversion |
| Chr9 | 22172435 | 22169503 | chr9 | 20009775 | 20012734 | Inversion |
| Chr9 | 22175686 | 22172954 | chr9 | 20006716 | 20009431 | Inversion |
| Chr9 | 22178082 | 22176784 | chr9 | 20004483 | 20005751 | Inversion |
| Chr9 | 22187467 | 22178428 | chr9 | 19995494 | 20004486 | Inversion |
| Chr9 | 22187793 | 22187466 | chr9 | 19995001 | 19995326 | Inversion |
| Chr9 | 22203239 | 22188162 | chr9 | 19979239 | 19994282 | Inversion |
| Chr9 | 22232090 | 22203240 | chr9 | 19950487 | 19979333 | Inversion |
| Chr9 | 22234830 | 22232085 | chr9 | 19943578 | 19946342 | Inversion |
| Chr9 | 22256206 | 22234874 | chr9 | 19922348 | 19943597 | Inversion |
| Chr9 | 22264573 | 22258919 | chr9 | 19916694 | 19922353 | Inversion |
| Chr9 | 22270461 | 22264573 | chr9 | 19910452 | 19916402 | Inversion |
| Chr9 | 22273643 | 22270462 | chr9 | 34128352 | 34131526 | Inversion |
| Chr9 | 22277606 | 22273643 | chr9 | 19906420 | 19910457 | Inversion |
| Chr9 | 22278324 | 22277606 | chr9 | 19905411 | 19906128 | Inversion |
| Chr9 | 22285198 | 22281248 | chr9 | 19900812 | 19904771 | Inversion |
| Chr9 | 22306697 | 22285195 | chr9 | 19878964 | 19900511 | Inversion |
| Chr9 | 22336074 | 22319964 | chr9 | 19862904 | 19878968 | Inversion |
| Chr9 | 22352072 | 22336594 | chr9 | 19847408 | 19862908 | Inversion |
| Chr9 | 22354322 | 22352507 | chr9 | 19845453 | 19847267 | Inversion |
| Chr9 | 22355755 | 22354322 | chr9 | 19844089 | 19845522 | Inversion |
| Chr9 | 22356718 | 22355750 | chr9 | 19831288 | 19832257 | Inversion |
| Chr9 | 22383855 | 22356770 | chr9 | 19804036 | 19831116 | Inversion |
| Chr9 | 22388387 | 22389579 | chr1 | 14496389 | 14497604 | Inter-translocation |
| Chr9 | 22394906 | 22391649 | chr9 | 27698692 | 27701949 | Inversion |
| Chr9 | 22395761 | 22395142 | chr9 | 19803421 | 19804039 | Inversion |
| Chr9 | 22423310 | 22399457 | chr9 | 19779570 | 19803428 | Inversion |
| Chr9 | 22439665 | 22423307 | chr9 | 19762403 | 19778731 | Inversion |
| Chr9 | 22454862 | 22453028 | chr9 | 19760574 | 19762408 | Inversion |
| Chr9 | 22491759 | 22454858 | chr9 | 19718011 | 19754947 | Inversion |
| Chr9 | 22505693 | 22491764 | chr9 | 19703546 | 19717488 | Inversion |
| Chr9 | 22522022 | 22505794 | chr9 | 19686124 | 19702424 | Inversion |
| Chr9 | 22542300 | 22522156 | chr9 | 19665944 | 19686121 | Inversion |
| Chr9 | 22552795 | 22542396 | chr9 | 19655569 | 19665938 | Inversion |
| Chr9 | 22572640 | 22553622 | chr9 | 19636442 | 19655468 | Inversion |
| Chr9 | 22573617 | 22572646 | chr9 | 19632834 | 19633784 | Inversion |
| Chr9 | 22577736 | 22573620 | chr9 | 19622354 | 19626474 | Inversion |
| Chr9 | 22582214 | 22577731 | chr9 | 19617522 | 19622016 | Inversion |
| Chr9 | 22583704 | 22582209 | chr9 | 19604274 | 19605702 | Inversion |
| Chr9 | 22584797 | 22583982 | chr9 | 19603446 | 19604263 | Inversion |
| Chr9 | 22589370 | 22585143 | chr9 | 19599223 | 19603449 | Inversion |
| Chr9 | 22597726 | 22589307 | chr9 | 19590822 | 19599222 | Inversion |
| Chr9 | 22610209 | 22597776 | chr9 | 19578400 | 19590824 | Inversion |
| Chr9 | 22625416 | 22610211 | chr9 | 19560197 | 19575386 | Inversion |
| Chr9 | 22628338 | 22626475 | chr9 | 19558238 | 19560095 | Inversion |
| Chr9 | 22663361 | 22628455 | chr9 | 19523288 | 19558137 | Inversion |
| Chr9 | 22673721 | 22663512 | chr9 | 19513082 | 19523289 | Inversion |
| Chr9 | 23019605 | 23018928 | chr5 | 8015922 | 8016600 | Inversion |
| Chr9 | 23107665 | 23109008 | chr8 | 19770598 | 19771926 | Inter-translocation |
| Chr9 | 23330915 | 23333558 | chr1 | 23926588 | 23929236 | Inter-translocation |
| Chr9 | 23477276 | 23477001 | chr6 | 3054885 | 3055161 | Inversion |
| Chr9 | 23755826 | 23756082 | chr5 | 8731021 | 8731277 | Inter-translocation |
| Chr9 | 23767436 | 23766915 | chr9 | 18699821 | 18700343 | Inversion |
| Chr9 | 23778858 | 23781202 | chr9 | 23745237 | 23747594 | Intra-translocation |
| Chr9 | 23781198 | 23782073 | chr9 | 23747875 | 23748752 | Intra-translocation |
| Chr9 | 23785590 | 23785398 | chr9 | 57818580 | 57818772 | Inversion |
| Chr9 | 24144472 | 24146787 | chr9 | 24257148 | 24259469 | Intra-translocation |
| Chr9 | 24361365 | 24366944 | chr8 | 18819215 | 18824796 | Inter-translocation |
| Chr9 | 24443627 | 24446127 | chr9 | 24363430 | 24365926 | Intra-translocation |
| Chr9 | 24446139 | 24446553 | chr9 | 24366199 | 24366612 | Intra-translocation |
| Chr9 | 24498383 | 24497158 | chr9 | 28463321 | 28464552 | Inversion |
| Chr9 | 24499720 | 24498424 | chr9 | 28105488 | 28106784 | Inversion |
| Chr9 | 24598982 | 24600821 | chr9 | 27758728 | 27760560 | Intra-translocation |
| Chr9 | 24730080 | 24730237 | chr8 | 11186720 | 11186877 | Inter-translocation |
| Chr9 | 24730082 | 24730347 | chr8 | 11364931 | 11365197 | Inter-translocation |
| Chr9 | 24742698 | 24742346 | chr7 | 26129597 | 26129949 | Inversion |
| Chr9 | 24875775 | 24876115 | chr4 | 32988035 | 32988375 | Inter-translocation |
| Chr9 | 24945106 | 24950403 | chr9 | 26266505 | 26271792 | Intra-translocation |
| Chr9 | 25017018 | 25018845 | chr9 | 33224004 | 33225831 | Intra-translocation |
| Chr9 | 25043778 | 25036122 | chr9 | 30241103 | 30248774 | Inversion |
| Chr9 | 25065292 | 25045981 | chr9 | 24997053 | 25016363 | Inversion |
| Chr9 | 25082406 | 25069103 | chr9 | 24983760 | 24997058 | Inversion |
| Chr9 | 25085171 | 25082450 | chr9 | 24981042 | 24983763 | Inversion |
| Chr9 | 25249137 | 25247755 | chr9 | 28995703 | 28997084 | Inversion |
| Chr9 | 25382690 | 25382941 | chr1 | 24676348 | 24676599 | Inter-translocation |
| Chr9 | 25553673 | 25557195 | chr9 | 30025430 | 30028952 | Intra-translocation |
| Chr9 | 25726814 | 25726594 | chr9 | 25680437 | 25680657 | Inversion |
| Chr9 | 26047643 | 26050943 | chr5 | 8301738 | 8305057 | Inter-translocation |
| Chr9 | 26196199 | 26191232 | chr8 | 8620294 | 8625272 | Inversion |
| Chr9 | 26245225 | 26238452 | chr8 | 19743306 | 19750078 | Inversion |
| Chr9 | 26664242 | 26665441 | chr8 | 18417108 | 18418304 | Inter-translocation |
| Chr9 | 26677147 | 26674400 | chr9 | 27551199 | 27553956 | Inversion |
| Chr9 | 26681641 | 26677150 | chr9 | 32484220 | 32488702 | Inversion |
| Chr9 | 26681655 | 26682374 | chr9 | 26379446 | 26380170 | Intra-translocation |
| Chr9 | 26726342 | 26722701 | chr9 | 25320238 | 25323890 | Inversion |
| Chr9 | 26739119 | 26740521 | chr1 | 26837690 | 26839092 | Inter-translocation |
| Chr9 | 26927851 | 26928908 | chr8 | 19999412 | 20000469 | Inter-translocation |
| Chr9 | 26935712 | 26936248 | chr9 | 26893991 | 26894528 | Intra-translocation |
| Chr9 | 27017068 | 27015795 | chr8 | 19536523 | 19537796 | Inversion |
| Chr9 | 27043774 | 27045818 | chr9 | 33477380 | 33479408 | Intra-translocation |
| Chr9 | 27052693 | 27053382 | chr5 | 25470286 | 25470973 | Inter-translocation |
| Chr9 | 27229662 | 27222865 | chr1 | 18609231 | 18616030 | Inversion |
| Chr9 | 27245762 | 27248332 | chr9 | 42245268 | 42247837 | Intra-translocation |
| Chr9 | 27252434 | 27253025 | chr4 | 35380339 | 35380929 | Inter-translocation |
| Chr9 | 27287293 | 27286680 | chr9 | 26662658 | 26663270 | Inversion |
| Chr9 | 27289712 | 27293157 | chr5 | 15952929 | 15956403 | Inter-translocation |
| Chr9 | 27506031 | 27504632 | chr5 | 8008367 | 8009758 | Inversion |
| Chr9 | 27541380 | 27541599 | chr9 | 32954895 | 32955114 | Intra-translocation |
| Chr9 | 27639943 | 27641836 | chr9 | 27914038 | 27915999 | Intra-translocation |
| Chr9 | 27716126 | 27717519 | chr8 | 8285747 | 8287140 | Inter-translocation |
| Chr9 | 27774684 | 27774914 | chr7 | 30549392 | 30549621 | Inter-translocation |
| Chr9 | 27780854 | 27786316 | chr9 | 31214561 | 31220044 | Intra-translocation |
| Chr9 | 27811813 | 27813603 | chr5 | 16293122 | 16294910 | Inter-translocation |
| Chr9 | 27813349 | 27814178 | chr9 | 19904768 | 19905597 | Intra-translocation |
| Chr9 | 28061830 | 28061472 | chr8 | 12081317 | 12081675 | Inversion |
| Chr9 | 28104898 | 28105473 | chr5 | 25146118 | 25146696 | Inter-translocation |
| Chr9 | 28115121 | 28112402 | chr9 | 24363439 | 24366149 | Inversion |
| Chr9 | 28358932 | 28359025 | chr2 | 28194106 | 28194199 | Inter-translocation |
| Chr9 | 28396629 | 28396989 | chr7 | 26162176 | 26162537 | Inter-translocation |
| Chr9 | 28430249 | 28429978 | chr3 | 13368313 | 13368584 | Inversion |
| Chr9 | 28627416 | 28620610 | chr8 | 19453349 | 19460161 | Inversion |
| Chr9 | 28630322 | 28628956 | chr9 | 33801228 | 33802606 | Inversion |
| Chr9 | 28731976 | 28710456 | chr9 | 30724737 | 30746269 | Inversion |
| Chr9 | 28740770 | 28731974 | chr9 | 30715971 | 30724795 | Inversion |
| Chr9 | 28742427 | 28738535 | chr9 | 30714974 | 30718815 | Inversion |
| Chr9 | 28744653 | 28741387 | chr9 | 30712076 | 30715346 | Inversion |
| Chr9 | 28769866 | 28744807 | chr9 | 30687011 | 30712076 | Inversion |
| Chr9 | 28770581 | 28770471 | chr9 | 30686906 | 30687016 | Inversion |
| Chr9 | 28784025 | 28781243 | chr9 | 30684125 | 30686910 | Inversion |
| Chr9 | 28791552 | 28784178 | chr9 | 30676752 | 30684127 | Inversion |
| Chr9 | 28808657 | 28791551 | chr9 | 30659191 | 30676333 | Inversion |
| Chr9 | 28815059 | 28809100 | chr9 | 30626483 | 30632448 | Inversion |
| Chr9 | 28835485 | 28836632 | chr8 | 8727862 | 8729008 | Inter-translocation |
| Chr9 | 28835687 | 28816127 | chr9 | 30606815 | 30626344 | Inversion |
| Chr9 | 28842671 | 28835722 | chr9 | 30598569 | 30605491 | Inversion |
| Chr9 | 28849474 | 28842673 | chr9 | 30591665 | 30598469 | Inversion |
| Chr9 | 28891583 | 28849528 | chr9 | 30543893 | 30585852 | Inversion |
| Chr9 | 28918597 | 28891579 | chr9 | 30495337 | 30522371 | Inversion |
| Chr9 | 28931913 | 28918697 | chr9 | 30482122 | 30495333 | Inversion |
| Chr9 | 28944503 | 28942582 | chr9 | 30480206 | 30482127 | Inversion |
| Chr9 | 28950044 | 28944506 | chr9 | 30474510 | 30480041 | Inversion |
| Chr9 | 28954253 | 28950045 | chr9 | 30469351 | 30473579 | Inversion |
| Chr9 | 28966595 | 28954249 | chr9 | 30450263 | 30462623 | Inversion |
| Chr9 | 28973182 | 28971760 | chr9 | 30450220 | 30451641 | Inversion |
| Chr9 | 28980075 | 28973175 | chr9 | 30437476 | 30444383 | Inversion |
| Chr9 | 28992546 | 28987690 | chr9 | 30432609 | 30437481 | Inversion |
| Chr9 | 29032507 | 29008847 | chr9 | 30408929 | 30432614 | Inversion |
| Chr9 | 29121708 | 29090567 | chr9 | 30371000 | 30402150 | Inversion |
| Chr9 | 29146545 | 29121703 | chr9 | 30333028 | 30357884 | Inversion |
| Chr9 | 29151172 | 29149230 | chr2 | 4010973 | 4012932 | Inversion |
| Chr9 | 29170090 | 29174230 | chr8 | 3064551 | 3068715 | Inter-translocation |
| Chr9 | 29184218 | 29178907 | chr9 | 30585928 | 30591236 | Inversion |
| Chr9 | 29188570 | 29187165 | chr8 | 21394058 | 21395461 | Inversion |
| Chr9 | 29203671 | 29202334 | chr9 | 30331691 | 30333027 | Inversion |
| Chr9 | 29229319 | 29203064 | chr9 | 30299908 | 30326129 | Inversion |
| Chr9 | 29233605 | 29233372 | chr9 | 30299680 | 30299913 | Inversion |
| Chr9 | 29242291 | 29233726 | chr9 | 30291108 | 30299680 | Inversion |
| Chr9 | 29257765 | 29242288 | chr9 | 30268976 | 30284451 | Inversion |
| Chr9 | 29265082 | 29261157 | chr9 | 30265047 | 30268976 | Inversion |
| Chr9 | 29270044 | 29265077 | chr9 | 30251955 | 30256924 | Inversion |
| Chr9 | 29274390 | 29270040 | chr9 | 30232898 | 30237235 | Inversion |
| Chr9 | 29295375 | 29274383 | chr9 | 30203754 | 30224752 | Inversion |
| Chr9 | 29299266 | 29295457 | chr9 | 29998109 | 30001967 | Inversion |
| Chr9 | 29300502 | 29299262 | chr9 | 29980638 | 29981878 | Inversion |
| Chr9 | 29313878 | 29300498 | chr9 | 29955567 | 29969004 | Inversion |
| Chr9 | 29325434 | 29314098 | chr9 | 29943905 | 29955278 | Inversion |
| Chr9 | 29339870 | 29328523 | chr9 | 29931630 | 29942986 | Inversion |
| Chr9 | 29343708 | 29339223 | chr9 | 29939145 | 29943630 | Inversion |
| Chr9 | 29345928 | 29342484 | chr9 | 29927086 | 29930531 | Inversion |
| Chr9 | 29354846 | 29345925 | chr9 | 29917808 | 29926740 | Inversion |
| Chr9 | 29360442 | 29354853 | chr9 | 29911703 | 29917305 | Inversion |
| Chr9 | 29369365 | 29360445 | chr9 | 29901785 | 29910703 | Inversion |
| Chr9 | 29371129 | 29369652 | chr9 | 29900317 | 29901793 | Inversion |
| Chr9 | 29375768 | 29371125 | chr9 | 29894310 | 29898972 | Inversion |
| Chr9 | 29384969 | 29376157 | chr9 | 29885470 | 29894312 | Inversion |
| Chr9 | 29387667 | 29384967 | chr9 | 29882003 | 29884702 | Inversion |
| Chr9 | 29387668 | 29397925 | chr9 | 29817925 | 29828217 | Intra-translocation |
| Chr9 | 29397535 | 29398584 | chr8 | 29154497 | 29155548 | Inter-translocation |
| Chr9 | 29398639 | 29401538 | chr9 | 29828209 | 29831108 | Intra-translocation |
| Chr9 | 29402023 | 29423629 | chr9 | 29831113 | 29852676 | Intra-translocation |
| Chr9 | 29419802 | 29424173 | chr9 | 30232898 | 30237236 | Intra-translocation |
| Chr9 | 29423780 | 29425725 | chr9 | 29852673 | 29854618 | Intra-translocation |
| Chr9 | 29425847 | 29437040 | chr9 | 29854618 | 29865846 | Intra-translocation |
| Chr9 | 29437037 | 29439089 | chr9 | 29865882 | 29867890 | Intra-translocation |
| Chr9 | 29440853 | 29451942 | chr9 | 29867888 | 29879057 | Intra-translocation |
| Chr9 | 29452602 | 29454452 | chr8 | 38075820 | 38077633 | Inter-translocation |
| Chr9 | 29457147 | 29460106 | chr9 | 29879054 | 29882005 | Intra-translocation |
| Chr9 | 29520561 | 29460107 | chr9 | 29757299 | 29817925 | Inversion |
| Chr9 | 29534799 | 29520456 | chr9 | 29743041 | 29757301 | Inversion |
| Chr9 | 29551807 | 29541463 | chr9 | 29730814 | 29741041 | Inversion |
| Chr9 | 29552480 | 29551979 | chr9 | 29720900 | 29721401 | Inversion |
| Chr9 | 29565959 | 29560627 | chr9 | 29715562 | 29720906 | Inversion |
| Chr9 | 29573168 | 29566143 | chr9 | 29708544 | 29715559 | Inversion |
| Chr9 | 29582994 | 29573164 | chr9 | 29690565 | 29700395 | Inversion |
| Chr9 | 29585256 | 29583594 | chr9 | 29688911 | 29690573 | Inversion |
| Chr9 | 29594905 | 29590208 | chr5 | 8878339 | 8882988 | Inversion |
| Chr9 | 29616022 | 29615808 | chr9 | 29688693 | 29688907 | Inversion |
| Chr9 | 29622357 | 29616168 | chr9 | 29682516 | 29688696 | Inversion |
| Chr9 | 29714554 | 29714831 | chr5 | 9321935 | 9322212 | Inter-translocation |
| Chr9 | 29716808 | 29719136 | chr1 | 24229665 | 24231984 | Inter-translocation |
| Chr9 | 29719138 | 29717502 | chr9 | 27843926 | 27845567 | Inversion |
| Chr9 | 29723195 | 29721675 | chr8 | 37778477 | 37780004 | Inversion |
| Chr9 | 29761023 | 29759444 | chr5 | 15118756 | 15120341 | Inversion |
| Chr9 | 29770948 | 29779370 | chr8 | 5349842 | 5358255 | Inter-translocation |
| Chr9 | 29787044 | 29786561 | chr3 | 47086871 | 47087353 | Inversion |
| Chr9 | 29865809 | 29869299 | chr8 | 7616499 | 7619991 | Inter-translocation |
| Chr9 | 29879164 | 29876399 | chr8 | 7607295 | 7610061 | Inversion |
| Chr9 | 29879165 | 29881609 | chr9 | 27278642 | 27281089 | Intra-translocation |
| Chr9 | 29923947 | 29917582 | chr9 | 29535127 | 29541504 | Inversion |
| Chr9 | 29928401 | 29926047 | chr4 | 6296076 | 6298400 | Inversion |
| Chr9 | 29929808 | 29928398 | chr4 | 6294243 | 6295653 | Inversion |
| Chr9 | 29931521 | 29929808 | chr9 | 29533445 | 29535131 | Inversion |
| Chr9 | 29940695 | 29931518 | chr9 | 29523886 | 29533083 | Inversion |
| Chr9 | 29946357 | 29940686 | chr9 | 29518090 | 29523762 | Inversion |
| Chr9 | 29962779 | 29957609 | chr9 | 29512921 | 29518094 | Inversion |
| Chr9 | 29969899 | 29962778 | chr9 | 29505940 | 29513014 | Inversion |
| Chr9 | 29977576 | 29970777 | chr9 | 29492910 | 29499722 | Inversion |
| Chr9 | 29986704 | 29978038 | chr9 | 29483152 | 29491798 | Inversion |
| Chr9 | 29990871 | 29988853 | chr9 | 29473062 | 29475079 | Inversion |
| Chr9 | 30001877 | 29990879 | chr9 | 29461898 | 29472892 | Inversion |
| Chr9 | 30010717 | 30001872 | chr9 | 29446317 | 29455130 | Inversion |
| Chr9 | 30029975 | 30010717 | chr9 | 29426941 | 29446227 | Inversion |
| Chr9 | 30053203 | 30029966 | chr9 | 29403650 | 29426875 | Inversion |
| Chr9 | 30054038 | 30053205 | chr9 | 29401433 | 29402266 | Inversion |
| Chr9 | 30067552 | 30054931 | chr9 | 29388765 | 29401435 | Inversion |
| Chr9 | 30072599 | 30071529 | chr9 | 29387693 | 29388761 | Inversion |
| Chr9 | 30074708 | 30072595 | chr9 | 29377705 | 29379829 | Inversion |
| Chr9 | 30077518 | 30080763 | chr9 | 40679357 | 40682596 | Intra-translocation |
| Chr9 | 30081712 | 30081273 | chr9 | 29376917 | 29377356 | Inversion |
| Chr9 | 30086510 | 30081708 | chr9 | 29365409 | 29370183 | Inversion |
| Chr9 | 30097151 | 30094611 | chr9 | 29362849 | 29365384 | Inversion |
| Chr9 | 30112495 | 30097146 | chr9 | 29346112 | 29361446 | Inversion |
| Chr9 | 30122943 | 30120512 | chr9 | 27784656 | 27787083 | Inversion |
| Chr9 | 30126175 | 30122950 | chr9 | 27781085 | 27784310 | Inversion |
| Chr9 | 30127360 | 30126177 | chr9 | 27779799 | 27780983 | Inversion |
| Chr9 | 30140967 | 30127627 | chr9 | 27766329 | 27779656 | Inversion |
| Chr9 | 30145415 | 30140959 | chr9 | 27751740 | 27756187 | Inversion |
| Chr9 | 30145751 | 30145444 | chr9 | 27748754 | 27749055 | Inversion |
| Chr9 | 30145781 | 30145716 | chr5 | 25643107 | 25643172 | Inversion |
| Chr9 | 30154947 | 30150496 | chr9 | 27744316 | 27748791 | Inversion |
| Chr9 | 30156795 | 30155335 | chr9 | 27742466 | 27743930 | Inversion |
| Chr9 | 30175666 | 30159408 | chr9 | 27723689 | 27739903 | Inversion |
| Chr9 | 30181714 | 30178031 | chr4 | 5633546 | 5637242 | Inversion |
| Chr9 | 30187912 | 30184738 | chr9 | 27720604 | 27723691 | Inversion |
| Chr9 | 30191783 | 30190953 | chr9 | 27717613 | 27718445 | Inversion |
| Chr9 | 30201817 | 30192563 | chr9 | 27707600 | 27716811 | Inversion |
| Chr9 | 30203885 | 30201815 | chr9 | 27696423 | 27698500 | Inversion |
| Chr9 | 30231093 | 30203926 | chr9 | 27669184 | 27696429 | Inversion |
| Chr9 | 30241924 | 30231093 | chr9 | 27658204 | 27669062 | Inversion |
| Chr9 | 30405678 | 30402296 | chr9 | 31608212 | 31611591 | Inversion |
| Chr9 | 30419818 | 30427974 | chr8 | 40037741 | 40045909 | Inter-translocation |
| Chr9 | 30453680 | 30452497 | chr9 | 30077996 | 30079223 | Inversion |
| Chr9 | 30458943 | 30454651 | chr9 | 30072947 | 30077240 | Inversion |
| Chr9 | 30516629 | 30515838 | chr9 | 31416315 | 31417106 | Inversion |
| Chr9 | 30790938 | 30791142 | chr3 | 14122284 | 14122488 | Inter-translocation |
| Chr9 | 30804500 | 30805607 | chr8 | 20035349 | 20036455 | Inter-translocation |
| Chr9 | 31239335 | 31239145 | chr9 | 41706514 | 41706708 | Inversion |
| Chr9 | 31240635 | 31240494 | chr8 | 14438799 | 14438933 | Inversion |
| Chr9 | 31257040 | 31256677 | chr7 | 33367159 | 33367522 | Inversion |
| Chr9 | 31272644 | 31275815 | chr8 | 20178075 | 20181251 | Inter-translocation |
| Chr9 | 31369583 | 31367572 | chr8 | 10029379 | 10031370 | Inversion |
| Chr9 | 31374064 | 31373720 | chr9 | 55809618 | 55809962 | Inversion |
| Chr9 | 31449189 | 31453837 | chr9 | 33369019 | 33373667 | Intra-translocation |
| Chr9 | 31558850 | 31556124 | chr2 | 48042039 | 48044774 | Inversion |
| Chr9 | 31709807 | 31708201 | chr3 | 42276511 | 42278121 | Inversion |
| Chr9 | 31766362 | 31766708 | chr7 | 27674536 | 27674882 | Inter-translocation |
| Chr9 | 31850502 | 31849703 | chr8 | 23037203 | 23038002 | Inversion |
| Chr9 | 31988710 | 31992983 | chr8 | 25189391 | 25193664 | Inter-translocation |
| Chr9 | 32063583 | 32063279 | chr6 | 32745227 | 32745540 | Inversion |
| Chr9 | 32072278 | 32072529 | chr8 | 25499332 | 25499582 | Inter-translocation |
| Chr9 | 32617799 | 32616859 | chr7 | 25254563 | 25255503 | Inversion |
| Chr9 | 32686064 | 32687406 | chr5 | 14817478 | 14818816 | Inter-translocation |
| Chr9 | 32783012 | 32782039 | chr9 | 28792661 | 28793634 | Inversion |
| Chr9 | 32909222 | 32908940 | chr7 | 33365126 | 33365408 | Inversion |
| Chr9 | 32959836 | 32952322 | chr8 | 2594514 | 2602045 | Inversion |
| Chr9 | 33026938 | 33023780 | chr8 | 11632978 | 11636135 | Inversion |
| Chr9 | 33074059 | 33074402 | chr2 | 3893788 | 3894132 | Inter-translocation |
| Chr9 | 33118995 | 33117579 | chr8 | 8118468 | 8119878 | Inversion |
| Chr9 | 33130605 | 33140535 | chr9 | 30238143 | 30248092 | Intra-translocation |
| Chr9 | 33145474 | 33147892 | chr8 | 3802476 | 3804893 | Inter-translocation |
| Chr9 | 33146226 | 33144475 | chr3 | 7453784 | 7455534 | Inversion |
| Chr9 | 33162017 | 33161582 | chr9 | 31925380 | 31925818 | Inversion |
| Chr9 | 33239850 | 33240068 | chr7 | 22223369 | 22223587 | Inter-translocation |
| Chr9 | 33240056 | 33240204 | chr7 | 22223739 | 22223887 | Inter-translocation |
| Chr9 | 33245811 | 33245461 | chr3 | 47381695 | 47382045 | Inversion |
| Chr9 | 33254958 | 33255081 | chr4 | 7414887 | 7415009 | Inter-translocation |
| Chr9 | 33333501 | 33334650 | chr3 | 23559168 | 23560323 | Inter-translocation |
| Chr9 | 33413667 | 33413434 | chr6 | 34652088 | 34652319 | Inversion |
| Chr9 | 33455205 | 33459123 | chr6 | 3224187 | 3228107 | Inter-translocation |
| Chr9 | 33529079 | 33528878 | chr3 | 50159327 | 50159528 | Inversion |
| Chr9 | 33583570 | 33585371 | chr9 | 29370184 | 29371984 | Intra-translocation |
| Chr9 | 33641709 | 33641942 | chr8 | 9609522 | 9609755 | Inter-translocation |
| Chr9 | 33641966 | 33647420 | chr8 | 7528771 | 7534223 | Inter-translocation |
| Chr9 | 34258505 | 34258230 | chr8 | 23437714 | 23437989 | Inversion |
| Chr9 | 34259777 | 34264739 | chr8 | 26106282 | 26111324 | Inter-translocation |
| Chr9 | 34465947 | 34465591 | chr8 | 3484551 | 3484907 | Inversion |
| Chr9 | 34708986 | 34709147 | chr1 | 17453264 | 17453425 | Inter-translocation |
| Chr9 | 34795397 | 34793330 | chr9 | 39185261 | 39187323 | Inversion |
| Chr9 | 35018401 | 35013598 | chr7 | 26388975 | 26393792 | Inversion |
| Chr9 | 35266997 | 35270689 | chr9 | 57813562 | 57817255 | Intra-translocation |
| Chr9 | 35676937 | 35676581 | chr8 | 37325076 | 37325432 | Inversion |
| Chr9 | 35678597 | 35679350 | chr9 | 36902257 | 36903009 | Intra-translocation |
| Chr9 | 35888954 | 35889157 | chr9 | 19067825 | 19068028 | Intra-translocation |
| Chr9 | 36040869 | 36041016 | chr8 | 2895562 | 2895709 | Inter-translocation |
| Chr9 | 36079715 | 36085932 | chr9 | 36856879 | 36863097 | Intra-translocation |
| Chr9 | 36221562 | 36221209 | chr3 | 23882628 | 23882983 | Inversion |
| Chr9 | 36608969 | 36608781 | chr4 | 2259203 | 2259391 | Inversion |
| Chr9 | 36723439 | 36728702 | chr9 | 13918018 | 13923280 | Intra-translocation |
| Chr9 | 37257520 | 37260132 | chr9 | 38462919 | 38465535 | Intra-translocation |
| Chr9 | 37382394 | 37378941 | chr8 | 33742435 | 33745884 | Inversion |
| Chr9 | 37383656 | 37382627 | chr8 | 33741150 | 33742178 | Inversion |
| Chr9 | 37383725 | 37387891 | chr8 | 4429466 | 4433632 | Inter-translocation |
| Chr9 | 37485579 | 37485925 | chr3 | 22270107 | 22270453 | Inter-translocation |
| Chr9 | 37593446 | 37593165 | chr9 | 33634231 | 33634515 | Inversion |
| Chr9 | 37725587 | 37728174 | chr9 | 38998398 | 39000984 | Intra-translocation |
| Chr9 | 37923560 | 37923651 | chr3 | 22857195 | 22857286 | Inter-translocation |
| Chr9 | 37936078 | 37936571 | chr9 | 35911695 | 35912175 | Intra-translocation |
| Chr9 | 37936773 | 37937068 | chr9 | 35912674 | 35912972 | Intra-translocation |
| Chr9 | 38025097 | 38016935 | chr8 | 3056407 | 3064550 | Inversion |
| Chr9 | 38192600 | 38192873 | chr5 | 8432800 | 8433073 | Inter-translocation |
| Chr9 | 38299523 | 38299876 | chr1 | 23880500 | 23880853 | Inter-translocation |
| Chr9 | 38830748 | 38831306 | chr3 | 11191889 | 11192434 | Inter-translocation |
| Chr9 | 38862206 | 38862731 | chr9 | 17575587 | 17576102 | Intra-translocation |
| Chr9 | 38869875 | 38870095 | chr8 | 23437711 | 23437931 | Inter-translocation |
| Chr9 | 38869882 | 38870145 | chr4 | 3300617 | 3300880 | Inter-translocation |
| Chr9 | 38941401 | 38942749 | chr9 | 33953568 | 33954911 | Intra-translocation |
| Chr9 | 39459747 | 39459638 | chr4 | 7355364 | 7355473 | Inversion |
| Chr9 | 39576630 | 39576373 | chr2 | 41493864 | 41494135 | Inversion |
| Chr9 | 39777579 | 39779935 | chr8 | 6777114 | 6779501 | Inter-translocation |
| Chr9 | 39820635 | 39820370 | chr7 | 23789456 | 23789721 | Inversion |
| Chr9 | 39938217 | 39938343 | chr3 | 5991364 | 5991490 | Inter-translocation |
| Chr9 | 39964813 | 39966186 | chr1 | 17898363 | 17899737 | Inter-translocation |
| Chr9 | 39967494 | 39966187 | chr9 | 29567498 | 29568806 | Inversion |
| Chr9 | 40082424 | 40086404 | chr9 | 41232250 | 41236219 | Intra-translocation |
| Chr9 | 40167575 | 40167311 | chr8 | 4264785 | 4265049 | Inversion |
| Chr9 | 40247277 | 40244816 | chr3 | 50006172 | 50008631 | Inversion |
| Chr9 | 40740189 | 40739830 | chr9 | 48736568 | 48736927 | Inversion |
| Chr9 | 40741390 | 40741575 | chr6 | 30358929 | 30359114 | Inter-translocation |
| Chr9 | 41111842 | 41112200 | chr5 | 9354117 | 9354474 | Inter-translocation |
| Chr9 | 41328519 | 41331674 | chr8 | 34485952 | 34489104 | Inter-translocation |
| Chr9 | 41518274 | 41519572 | chr8 | 10143844 | 10145143 | Inter-translocation |
| Chr9 | 42666673 | 42666332 | chr5 | 15553914 | 15554258 | Inversion |
| Chr9 | 43219557 | 43220561 | chr3 | 39050343 | 39051360 | Inter-translocation |
| Chr9 | 43221868 | 43220310 | chr1 | 5064752 | 5066287 | Inversion |
| Chr9 | 43349054 | 43348656 | chr9 | 23112010 | 23112408 | Inversion |
| Chr9 | 44092553 | 44092198 | chr9 | 49012019 | 49012374 | Inversion |
| Chr9 | 44716659 | 44716858 | chr8 | 38981049 | 38981248 | Inter-translocation |
| Chr9 | 46250909 | 46251366 | chr1 | 41160298 | 41160752 | Inter-translocation |
| Chr9 | 46251293 | 46251916 | chr7 | 26516672 | 26517291 | Inter-translocation |
| Chr9 | 46875742 | 46867001 | chr4 | 37222243 | 37230984 | Inversion |
| Chr9 | 47553894 | 47554248 | chr2 | 47519149 | 47519503 | Inter-translocation |
| Chr9 | 47729938 | 47729594 | chr9 | 37542354 | 37542696 | Inversion |
| Chr9 | 48213019 | 48212656 | chr7 | 34813711 | 34814074 | Inversion |
| Chr9 | 48366586 | 48366861 | chr4 | 4796898 | 4797173 | Inter-translocation |
| Chr9 | 48551529 | 48551014 | chr9 | 51656117 | 51656626 | Inversion |
| Chr9 | 49184128 | 49184488 | chr4 | 3548544 | 3548904 | Inter-translocation |
| Chr9 | 49184491 | 49184326 | chr2 | 3120504 | 3120669 | Inversion |
| Chr9 | 49441453 | 49441103 | chr4 | 7549456 | 7549806 | Inversion |
| Chr9 | 50091399 | 50091124 | chr6 | 1491763 | 1492038 | Inversion |
| Chr9 | 50274798 | 50274292 | chr1 | 16664301 | 16664807 | Inversion |
| Chr9 | 50312711 | 50312428 | chr3 | 3243394 | 3243677 | Inversion |
| Chr9 | 50620711 | 50620991 | chr9 | 52806943 | 52807223 | Intra-translocation |
| Chr9 | 51525066 | 51524713 | chr4 | 4429887 | 4430241 | Inversion |
| Chr9 | 51982982 | 51983229 | chr8 | 12746619 | 12746863 | Inter-translocation |
| Chr9 | 52276532 | 52276180 | chr3 | 7292074 | 7292426 | Inversion |
| Chr9 | 52366869 | 52366516 | chr3 | 23286946 | 23287299 | Inversion |
| Chr9 | 52731858 | 52732140 | chr9 | 56475990 | 56476272 | Intra-translocation |
| Chr9 | 52786111 | 52785758 | chr6 | 17844582 | 17844939 | Inversion |
| Chr9 | 54484890 | 54484539 | chr7 | 26491834 | 26492185 | Inversion |
| Chr9 | 54528911 | 54528628 | chr4 | 38404184 | 38404467 | Inversion |
| Chr9 | 54560764 | 54560411 | chr1 | 26127631 | 26127983 | Inversion |
| Chr9 | 55019918 | 55020188 | chr9 | 54947272 | 54947542 | Intra-translocation |
| Chr9 | 55027334 | 55027258 | chr9 | 53256967 | 53257043 | Inversion |
| Chr9 | 55108722 | 55108936 | chr6 | 3198381 | 3198594 | Inter-translocation |
| Chr9 | 55271677 | 55271957 | chr1 | 41264402 | 41264682 | Inter-translocation |
| Chr9 | 55332668 | 55333025 | chr9 | 40541817 | 40542174 | Intra-translocation |
| Chr9 | 55795453 | 55795171 | chr1 | 25387048 | 25387330 | Inversion |
| Chr9 | 55921832 | 55920702 | chr9 | 27807338 | 27808484 | Inversion |
| Chr9 | 55924426 | 55924974 | chr9 | 27809581 | 27810131 | Intra-translocation |
| Chr9 | 55925330 | 55924966 | chr8 | 4605334 | 4605698 | Inversion |
| Chr9 | 56195264 | 56204169 | chr9 | 57946818 | 57955719 | Intra-translocation |
| Chr9 | 56222650 | 56222293 | chr4 | 4476228 | 4476585 | Inversion |
| Chr9 | 57136379 | 57136050 | chr9 | 5638240 | 5638565 | Inversion |
